# Metabolomics-based exploration the response mechanisms of *Saussurea involucrata* leaves under different levels of low temperature stress

**DOI:** 10.1186/s12864-023-09376-4

**Published:** 2023-06-01

**Authors:** Qi Sun, Lihua Ma, Xinxia Zhu

**Affiliations:** Key Laboratory of Xinjiang Phytomedicine Resource and Utilization of Ministry of Education, College of Life Sciences, Shihezi University, Shihezi, 832003 China

**Keywords:** Saussurea involucrata, Metabolism, Low temperature stress, Metabolic network

## Abstract

**Background:**

*Saussurea involucrata (Sik.)* is alpine plant that have developed special adaptive mechanisms to resist adverse environmental conditions such as low temperature chilling during long-term adaptation and evolution. Exploring the changes of its metabolites under different temperature stresses is helpful to gain insight into its cold stress tolerance.

**Methods:**

Ultra-performance liquid chromatography and tandem mass spectrometry were used to analyze the metabolites in the leaves of *Sik*. under low different temperature stress conditions. Results: A total of 753 metabolites were identified, and 360 different metabolites were identified according to the Kyoto Encyclopedia of Genes and Genomes (KEGG) involved in the biosynthesis of secondary metabolites and amino acids and sugars. Sucrose and trehalose synthesis, glycolysis, tricarboxylic acid cycle, pentose phosphate pathway, glutamic acid-mediated proline biosynthesis, purine metabolism, amino acid metabolism, phenylpropane synthesis pathway metabolites all respond to low temperature stress. Under cold stress conditions, carbohydrates in *Sik*. leaves accumulate first than under freezing conditions, and the lower the temperature under freezing conditions, the less amino acids accumulate, while the phenolic substances increase. The expression of various substances in LPE and LPC increased more than 10-fold after low temperature stress compared with the control, but the content of LPE and LPC substances decreased after cold adaptation. In addition, purines and phenolics decreased and amino acids accumulated significantly under freezing conditions. Conclusion: The metabolic network of *Sik*. leaves under different low temperature stress conditions was proposed, which provided a reference for further exploration of the metabolic mechanism related to low temperature stress tolerance of *Sik*.

**Supplementary Information:**

The online version contains supplementary material available at 10.1186/s12864-023-09376-4.

## Introduction

Low temperature stress is a common abiotic stress in the natural environment, which has an important impact on the growth and development of crops and their geographical distribution [1, 2]. When plants suffer from low-temperature stress, their external morphology and physiological functions will change, and the changes in external morphology are manifested as leaf wilting, withering, etc., and changes in physiological functions such as cell membrane dysfunction [3] and weakening of respiration [4]. However, many temperate crops, such as *Arabidopsis thaliana* [5], grapes [6], wheat [7] etc., have developed the ability to cope with freezing stress through cold adaptation. Cold adaptation is conditioned by first exposure to low, non-freezing temperatures, thereby developing coping capacity during subsequent cold stress [8]. Despite freezing conditions (< 0°), plants are cold domesticated to maintain overall health through metabolic networks [9]. Tibetan sheephead snowdrops were found to be semi-lethal after variable temperature incubation at 4 °C and 0 °C (day and night) from − 3.5 °C to -12 °C [10].

*Sik*. is not only an alpine herb of the Asteraceae family but also a traditional Chinese medicine. *Sik*. is rich in flavonoids [11], terpenes[12], alkaloids and other active substances, which have a certain preventive effect on relieving analgesia, anti-allergy and cancer hyperlipidemia [13], and have special medicinal value [14]. During the growth process, *Sik*. will experience sharp temperature changes similar to summer to winter within one day, and after long-term natural selection, it has formed a physiological and biochemical mechanism adapted to extreme environmental conditions, which has an important impact on the research on plant cold resistance and drought resistance. Tomatoes exhibit higher cold tolerance and photosynthetic efficiency after switching to the *Sik*. cold response factor *SiFBA5* [15]. In addition, the *SiDhn2* [16], *SiDHN* genes [17], *SiICE1* and *SiICE2* transcriptional regulators [18] in *Sik*. have been studied to potentially improve plant tolerance to abiotic stress. Transcriptomics of *Sik*. in terms of temperature stress [19] and adaptation to high altitude [20] has been studied, but the metabolite changes of *Sik*. at low temperatures remain unclear.

Metabolomics can reflect the adaptability of plants to the environment based on changes in the type and number of metabolic species identified. With the continuous advancement of bioinformatics, metabolomics has been frequently used to analyze plant stress [21]. When faced with low temperature stress, plant leaves usually undergo wilting, yellowing, curling and other changes, and plants will have growth and development inhibition [22], and some metabolites closely related to these reactions have also changed [23]. Quinoa seedlings were found to accumulate a large amount of soluble sugar under low temperature conditions and change the metabolic network under frost damage [24], and straw mushrooms are involved amino acid metabolism, carbohydrate metabolism, TCA cycle, energy metabolism, etc. at low temperatures [25]. Flavonoid biosynthesis, carbohydrate metabolism, amino acid metabolism, lipid metabolism and signaling pathways in Liriope spicata are significantly enriched in response to low temperature stress [26]. Differential expression of P-coumarinamide, D-proline, betaine, and chlorogenic acid metabolites in wheat under low temperature conditions, and seven upregulated metabolites and eight upregulated key enzymes were also closely involved in the biosynthetic pathway of sucrose and amino acids [27]. In addition, evergreen overwintering plants were found to gradually cool down in winter, anthocyanins, glucose, fructose accumulate in large quantities, chlorophyll degradation, and the closure of photosystem II reaction centers, and these changes help improve cold tolerance [28].

In summary, metabolomics is an effective method to explain the growth and adaptation mechanism of plants in harsh environments and has been widely used to study plant responses to abiotic stresses. However, as far as we know, there are currently few studies on the changes in *Sik*. metabolites under low temperature stress. Four low temperature treatment groups were set according to the habitat conditions experienced by *Sik.* during the day and the different response mechanisms of plants to chilly, cold, freezing and cold adaptation [29], they were divided into CK group 20° (control group), T1 group 20 °→ 4 °( 24 h), T3 group was 20 °→ -4 °( 12 h), T2 group 20 ° → -13 ° ( 3 h), T4 group 20 °→ 4 °( 12 h)→ 0 °( 6 h)→ -4 °( 3 h)→ -13 °( 3 h), by ultra-performance liquid chromatography and tandem mass spectrometry ( UPLC-MS / MS ), the results reported in this paper will help to better understand the response of metabolites of *Sik*. to low temperature stress.

## Results

### Low temperature stress of Sik

**Fig. 1:**
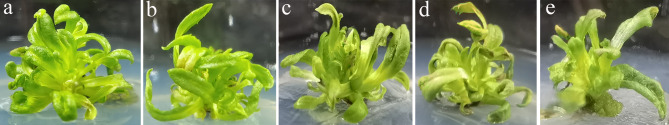
The phenotypes of (**a**) CK group; (**b**) T1 group; (**c**) T3 group; (**d**) T2 group; (**e**) T4 group

The most visual effect of low temperature stress on the plants was morphological changes. Significant differences in phenotype were observed in 2-month-old *Sik.* subjected to low-temperature stress. *Sik.* leaves were full, robust and brightly colored under ambient conditions (Fig. 1a), and there were no significant changes in leaf appearance after 20 °→ 4 °( 24 h) treatment (Fig. 1b). However, the leaves began to show slight chlorosis and yellowing after 20 °→ -4 °( 12 h) treatment (Fig. 1c), and the leaf color became darker, and the leaf color further darkened at 20 °→ -13 °( 3 h) (Fig. 1d). The leaves appeared water-stained and yellowed seriously, showing obvious signs of cold damage. After cold adaptation petiole softened and downward bending, leaf wilting, inward curling serious, but the yellowing phenomenon is alleviated (Fig. 1e). Sub-zero temperature will make *Sik.* leaves water deficit wilting condition, in -13 ° freezing environment will cause local tissue necrosis of *Sik.* leaves, but after cold taming by cold damage to alleviate the impact.

### Qualitative and quantitative metabolites

**Fig. 2:**
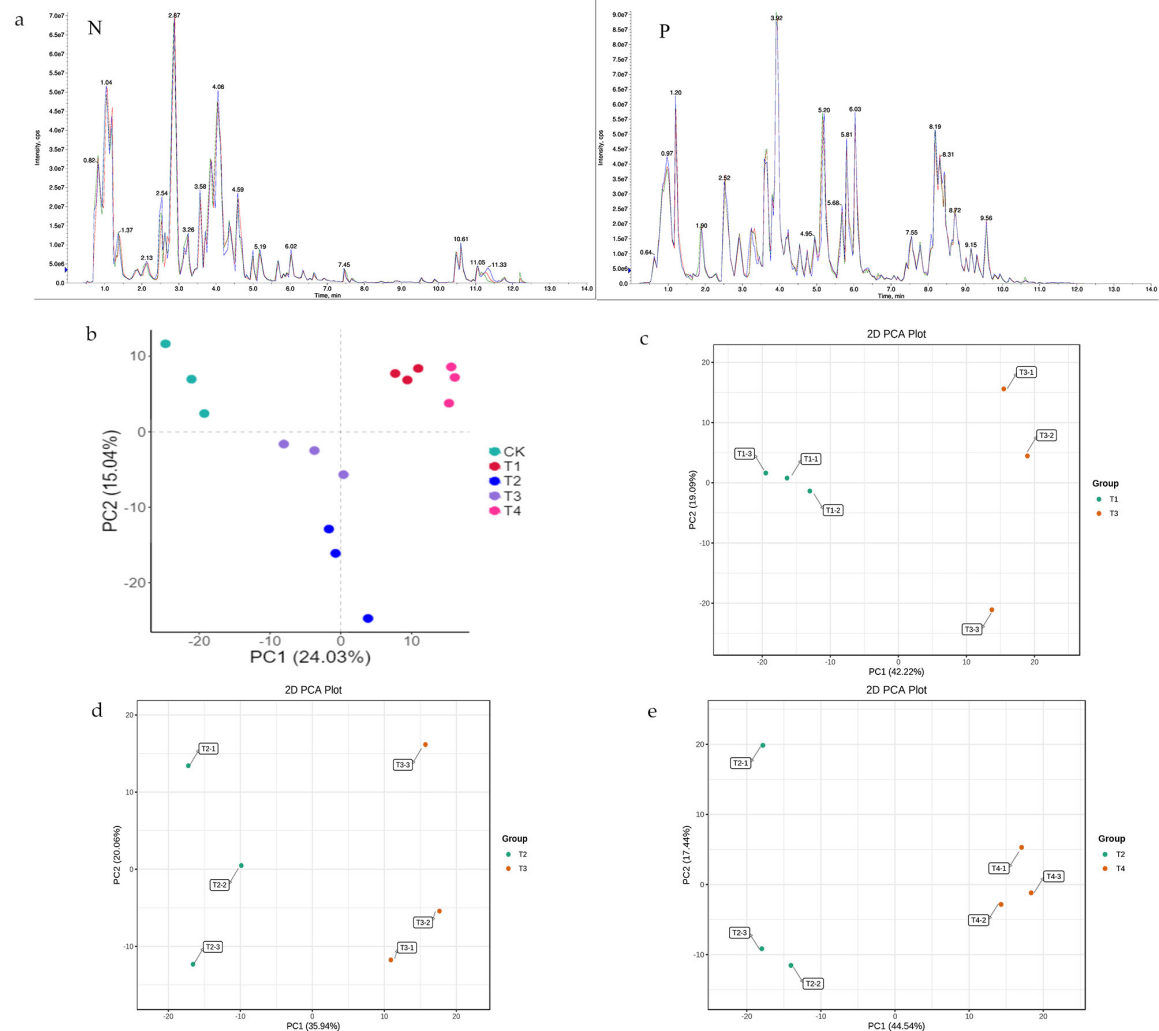
(**a**) Superimposed total ion flow diagram (TIC) of QC-like essential spectrum detection. Where N represents negative ion mode and P represents positive ion mode. Principal component analysis (PCA) between different low temperature treatment. (**b**) CK vs. T1/T2/T3/T4; (**c**) T1 vs. T3; (**d**) T2 vs. T3; (**e**) T2 vs. T4

A total ion current (TIC) plot and multimodal detection plot for a quality control (QC) sample are shown in Fig. 2a. The TIC plot represents a continuous description of the intensity and continuity of all ions in the mass spectrum at different time points. The curves for the total ion flow overlap greatly, and the retention time and peak intensity are the same, indicating better signal stability for the same sample when detecting mass spectrometry at different times.

The multimodal detection plot of metabolites in multiple reaction monitoring (MRM) mode shows that our sample contains multiple substances. Each mass-spectrometric peak of a different color represents the detected metabolite. Based on the local metabolite database, qualitative and quantitative mass spectrometry analysis of the metabolites in the samples was performed. A total of 752 metabolites were identified, including 155 phenolic acids, 119 flavonoids, 38 sugars and alcohols, 57 lipids, 66 amino acids and derivatives, 51 organic acids and derivatives, 36 nucleotides and derivatives, 33 lignin and coumarin, 2 quinones, 50 alkaloids, 19 terpenes, 15 vitamins and derivatives and 15 other metabolites. Details of all identified metabolites are shown in Table S1.

### PCA and PCC Analysis

In order to compare the metabolite composition of *Sik*. under four different cold stresses, the principal component analysis (PCA) was performed. PCA was performed on samples, and PCA provides insight into overall metabolic differences between samples and within-group variation [30]. The PCA plot shows that the *Sik*. under five conditions is clearly separated and the three biological replicates of each condition are clustered together (Fig. 2b),, indicating that the experiment is reproducible and reliable. The plants were significantly separated from the control CK group in the four low-temperature treatments, and there were also different degrees of separation between the four groups, indicating that there were significant differences in metabolite content between each group, which in turn indicated that the accumulation of different metabolites occurred after being subjected to different conditions of low-temperature stress. The T1/T3 group and the medium PCA map also showed obvious separation (Fig. 2c), indicating that the reaction mechanism of low temperature and freezing temperature were different, and the T2/T4 group and the medium PCA map also showed obvious separation indicating that the metabolites accumulated after cold adaptation were also different (Fig. 2d), and the obvious separation between the T2/T3 group and the medium PCA map indicated that the metabolites also changed under freezing and extreme freezing conditions (Fig. 2e).

The Pearson correlation coefficient (PCC) can be used to observe biological replicates between samples within groups by analysis of the correlation between samples. At the same time, the higher the correlation coefficient of the samples in the group relative to the samples between the groups, the more reliable the different metabolites obtained. Generally speaking, when 0.8≤|r|≤1, it can be considered that the two variables are highly correlated, 0.5≤|r|≤0.8 moderately correlated, 0.3≤|r|≤0.5 low correlation, the experimental data shows that the Pearson correlation coefficient in each group is 0.8≤|r|≤1, and the samples between groups are also at 0.5≤|r| indicating that the biological reproducibility in each group is high and the differential metabolites are reliable (Figure S1). These results showed that the experimental data were reliable and that different low temperature conditions strongly affected the metabolite profile of snow lotus.

### Orthogonal partial least squares-discriminant analysis

**Fig. 3:**
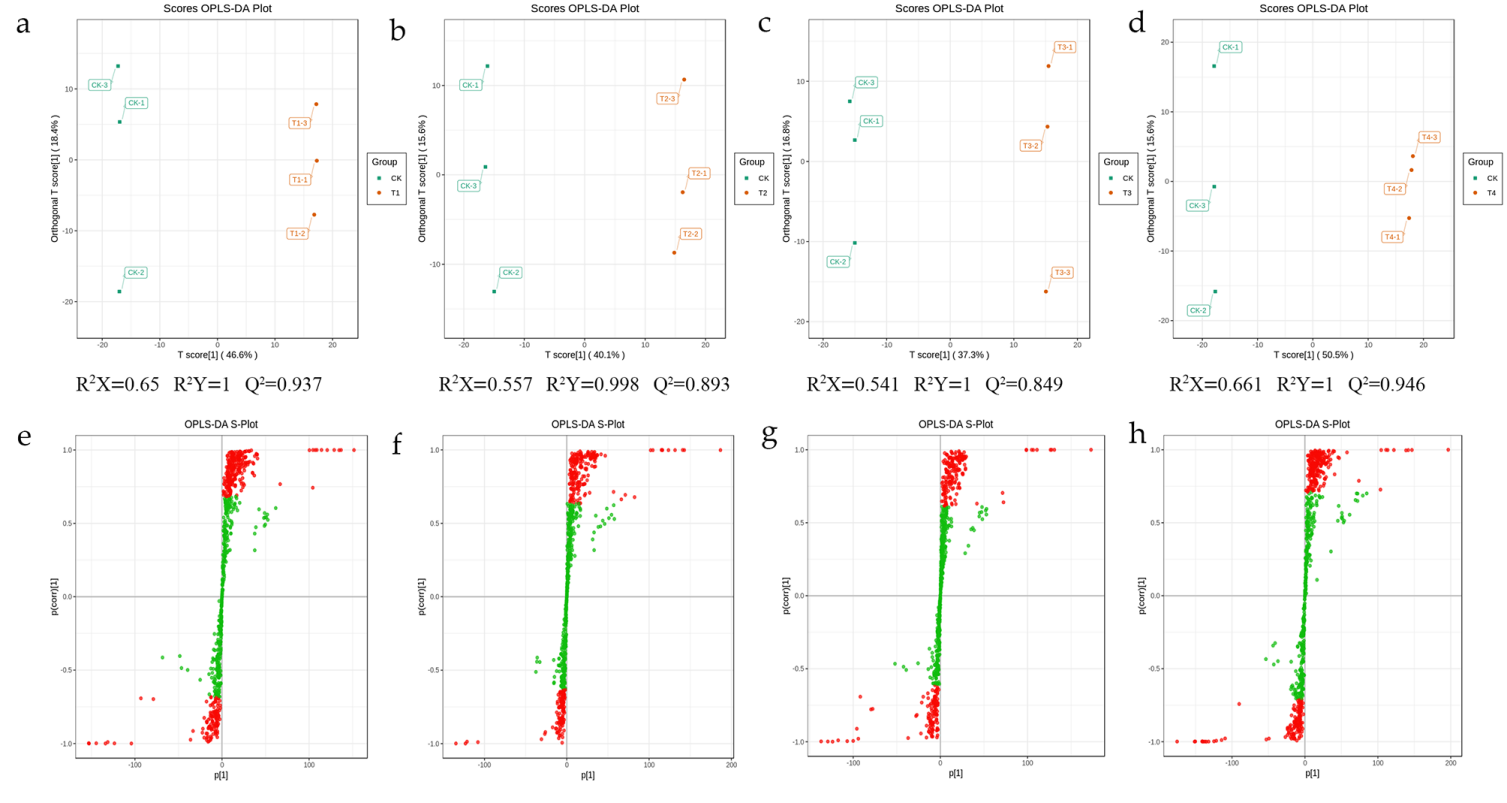
Orthogonal partial least squares-discriminant analysis (OPLS–DA) scores. Scores of the OPLS–DA model with (**a**)CK vs. T1; (**b**)CK vs. T2; (**c**)CK vs. T3; (**d**)CK vs. T4; OPLS-DA S-plot model with: (**e**)CK vs. T1; (**f**)CK vs. T2; (**g**)CK vs. T3; (**h**)CK vs. T4. R^2^Yscores and Q^2^ values represent the interpretation rate of the model to the Y matrix and the prediction ability of the model,respectively. When Q^2^ > 0.5, the model can be considered an effective model, and Q^2^ > 0.9 is an excellent model.mode

To find differential metabolites, orthogonal partial least squares discriminant analysis (OPLS-DA) was used to extract components in the independent variable X and the dependent variable Y, and then correlations between components were calculated. OPLS-DA combines orthogonal signal correction (OSC) and PLS-DA methods to decompose the X matrix information into two types of correlated and uncorrelated differences with Y. The differential variables were then screened by removing the uncorrelated differences. The results showed that all R^2^X were higher than 0.54. All R^2^Y scores were higher than 0.99 and all Q^2^ values were greater than 0.84 at CK vs. T1/T2/T3/T4, respectively (Fig. 3a-d), confirming that the differential metabolites responded to low temperature stress treatment. The S-plot map of OPLS-DA is shown in Fig. 3e-h. The OPLS-DA model was also validated by 200 random alignment and combinatorial alignment tests.

### Differential Metabolite Screening

**Fig. 4:**
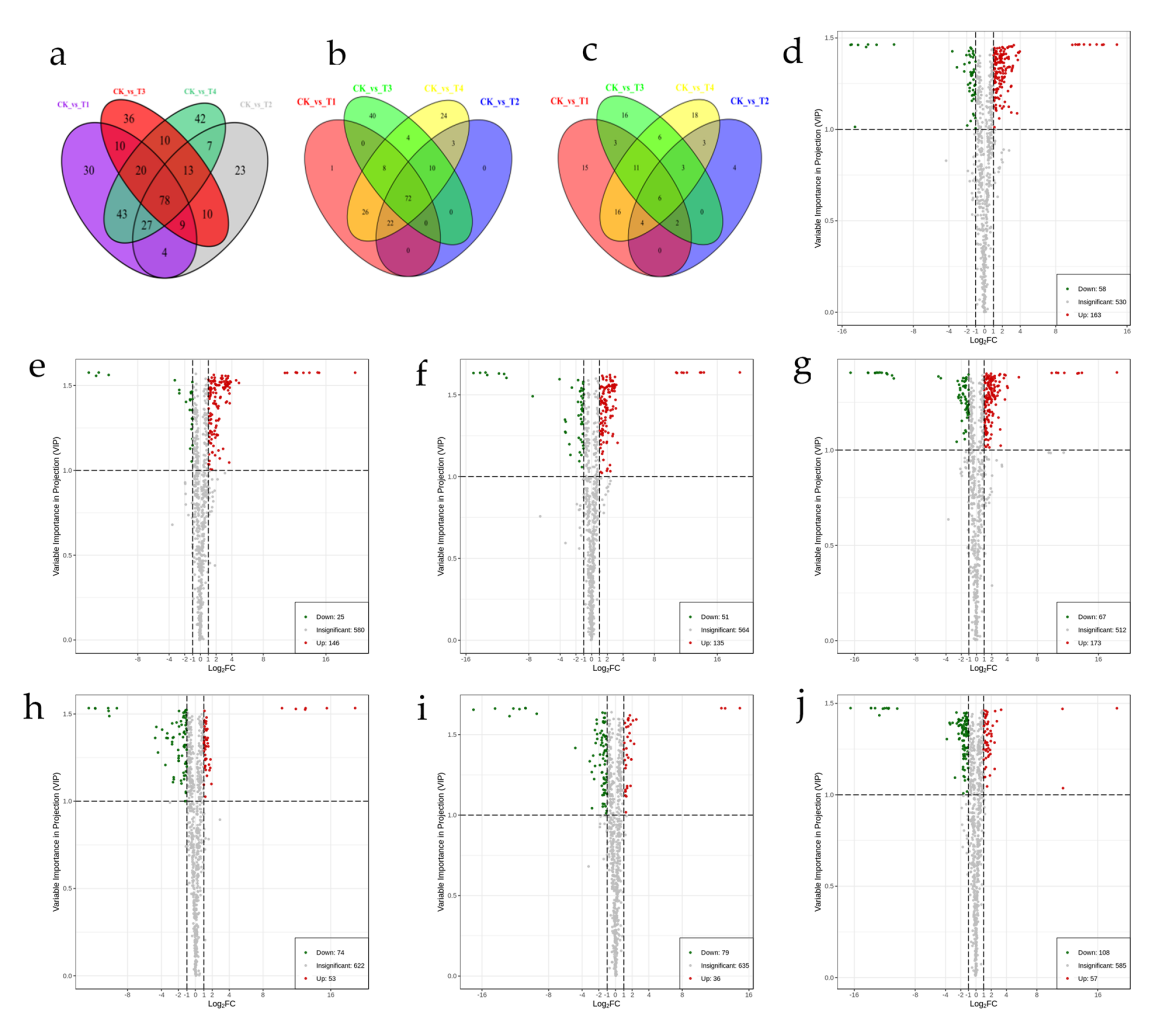
Differential metabolite analysis of Saussurea involucrata under low temperature stress. (**a**) Venn diagram of DEGs. (**b**) Venn diagram of up-regulated DEGs. (**c**) Venn diagram of down-regulated DEGs. Volcano maps of differential metabolites in different pairwise comparisons: (**d**) CK vs. T1; (**e**) CK vs. T2; (**f**) CK vs. T3; (**g**) CKvsT4; (**h**) T1vsT3; (**i**) T2vsT3; (**j**) T2vsT4.

Based on fold change ≥ 2 or ≤ 0.5 and VIP ≥ 1, a total of 362 differential metabolites (DEMs) were detected in the CK vs. T1/T2/T3/T4 group (Table S2). Venn plots show common and unique differences between cold treatment and control metabolomes(Fig. 4a-c). The results showed that in the overlapping regions of the Venn diagram, 72 upregulated differential genes and 6 downregulated differential genes were obtained, respectively. Volcano maps of differential metabolites in different pairwise comparisons are shown in Fig. 4d-j. In the CK vs. T4 group, there were more metabolites involved in cold regulation. There were 221 significantly different metabolites (163 up-regulated, 58 down-regulated) between CK vs. T1, 171 significantly different metabolites (146 up-regulated, 25 down-regulated) between CKvsT2, 186 markedly different metabolites (135 up-regulated, 51 down-regulated), and 240 significantly different metabolites (173 up-regulated, 67 down-regulated) between CK vs. T4. Interestingly, all upregulated differential metabolites were detected in the T2 cryogroup overlapping with the other groups, and only 6 upregulated differential metabolites were unique. There were 127 significantly different metabolites (53 up-regulated, 74 down-regulated) between T1vsT3 groups, 165 markedly different metabolites (67 up-regulated, 98 down-regulated) between T2 vs. T4, and 115 significantly different metabolites (36 up-regulated, 79 down-regulated) between T2 vs. T3. These metabolites are divided into 10 groups: amino acids and their derivatives, phenolic acids, lipids, organic acids, nucleotides and their derivatives, lignans and coumarins, flavonoids, alkaloids, terpenes and others. Metabolite changes were concentrated in Lipids, Phenolic acids, Flavonoids, Nucleotides and derivatives, Amino acids and derivatives (Table 1). In particular, more than half of these differential metabolites are secondary metabolites, including phenolic acids, flavonoids, flavonols, dihydroflavones, isoflavones, coumarins, and alkaloids.

### Functional annotation and KEGG Enrichment

**Fig. 5:**
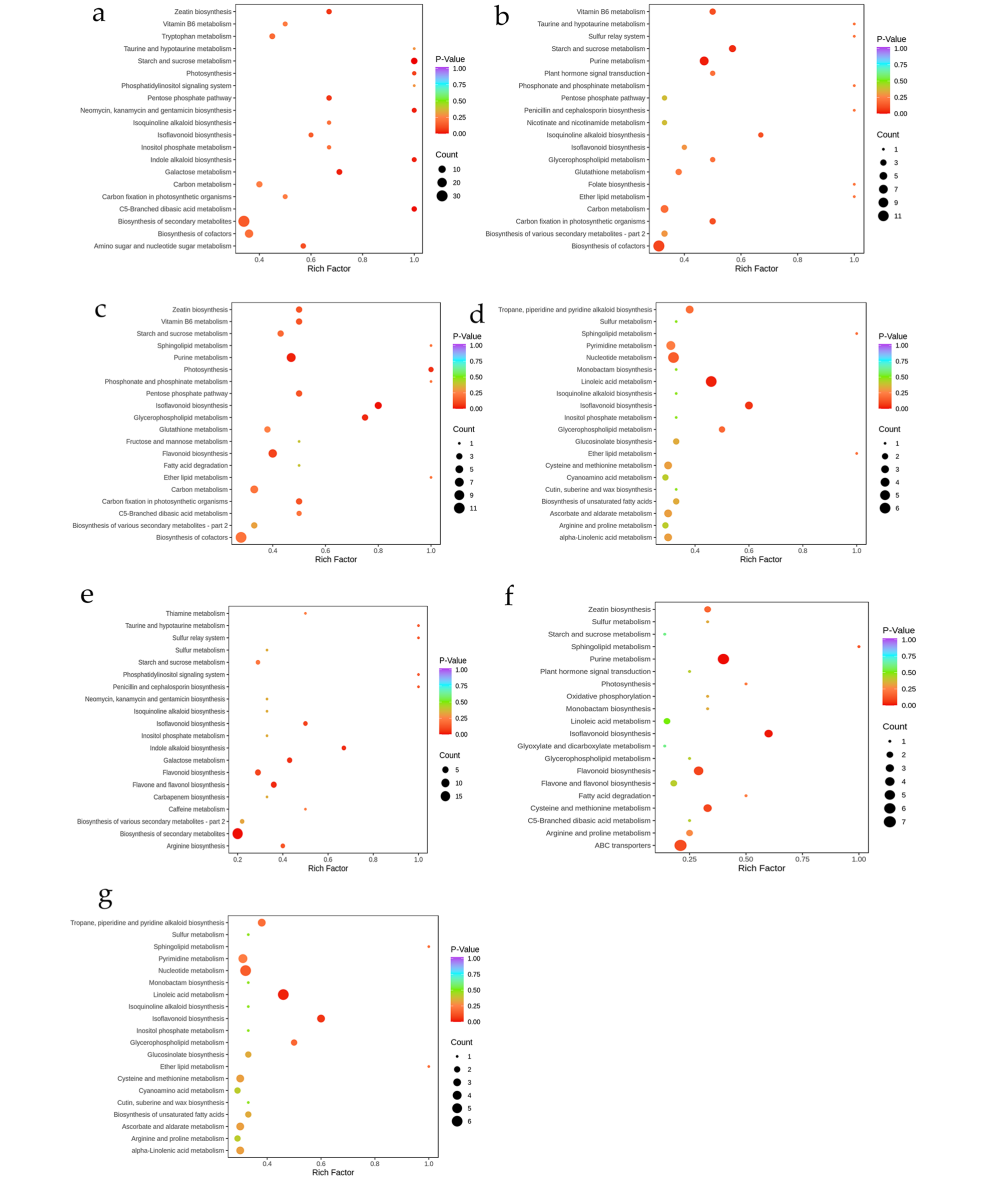
Pathway analysis of differential metabolites for seven comparison groups. (**a**) CK vs. T1; (**b**) CK vs. T2; (**c**) CK vs. T3; (**d**) CK vs. T4; (**e**) T1 vs. T3; (**f**) T2 vs. T4; (**g**) T2 vs. T3. Each bubble in the plot represents a metabolic pathway whose abscissa and bubble size jointly indicate the magnitude of the impact factors of the pathway. A larger bubble size indicates a larger impact factor. The bubble colors represent the p-values of the enrichment analysis, with darker colors showing a higher degree of enrichment

The annotated results of the significantly different metabolites KEGG were classified by pathway type in KEGG, and the differential metabolites in the CK vs. T1/T2/T3/T4 group involved metabolic pathways, secondary metabolite biosynthesis, cofactor biosynthesis, amino acid biosynthesis, and purine metabolism. In addition to the above, T1vsT3 is also involved in the biosynthesis of flavonoids, T2 vs. T4 is involved in nucleotide metabolism, linolenic acid metabolism, and T2 vs. T3 is involved in flavonoid metabolism. Interestingly, the proportion of metabolites in the ATP binding cassette (ABC) transport pathway increased significantly in all three comparison groups (Figure S2). Based on the results of differential metabolites, the KEGG pathway of DEMs was performed on four different conditions of *Sik.*. The main metabolic pathway changed in the CK vs. T1 group are starch and sucrose metabolism, C5-branched dibasic acid metabolism, galactose metabolism, etc.; when the temperature drops to -13°, the more obvious channels are purine metabolism, starch and sucrose metabolism, and biosynthesis of cofactors; CK vs. T3 from which it can be seen that compared with the CK vs. T1 group, the pathway change is significantly reduced, but in the comparison of.

sub-zero low temperature, we find isoflavonoid biosynthesis, purine metabolism, glycerophospholipid metabolism, photosynthesis, etc. After cold adaptation, and the significantly changed pathways are Starch and sucrose metabolism, vitamin B6 metabolism, glycerophospholipid metabolism, C5-Branched dibasic acid metabolism, and metabolism of glycine, serine and threonine. The differential metabolic pathways under cold and freezing temperature (T1 vs. T3) conditions were mainly focused on indoleic acid biosynthesis, isoflavone and flavonoid biosynthesis pathways. The differences between the freezing and cold-tamed groups (T2 vs. T4) mainly showed significant differences in linoleic acid metabolism, isoflavone biosynthesis and nucleotide metabolic pathways, and the differential metabolic pathways in the different freezing temperature groups (T2 vs. T3) were mainly purine metabolism, isoflavone biosynthesis and nucleotide metabolic pathways (Fig. 5a-g).

### Differential Metabolites Analysis

#### Low-Temperature-Induced lipid changes

A total of 133 lipids, 59 free fatty acids, 25 lysophosphatidylethanolamine (LPE) and 27 lysophosphatidylcholine (LPC) were detected at low temperatures. Compared with the control group, the expression of multiple substances in LPE and LPC was increased by more than 10 times in the T1/2/3/4 group (Figure S3), and the detected LPE and LPC were increased or did not change significantly, except for the decrease in the content of LysoPC 20:4 in CK vs. T3/4 group. The only detected glycophosphate choline (GPC) was upregulated in all four groups. However, the lipid content of T2vsT4 (Table S5) and T2vsT3 (Table S4) decreased significantly. No significant lipid changes in T1 vs. T3 group (Table S3).

#### Low-temperature-induced changes in phenylpropanoid pathway

Phenolic acids are a secondary metabolite containing phenolic rings. In this study, we identified 11 phenolic acid compounds with significant differences in all four groups compared with CK under four low temperature conditions in *Sik*. leaves, including 4-nitrophenol, caffeic acid, salicylacetic acid, sinapinaldehyde, chlorogenic acid methyl ester, caffeoylferuloylquinic acid, 3,4-O-dicaffeoylquinic acid methyl ester, 1,4-O-dicaffeoyl-3-O-succinoyl-quinic acid, 1,5-O-dicaffeoyl-3-O-succinoyl-quinic acid, dicaffeoylquinic acid-O-glucoside, 3,5-di-O-caffeoyl-1-O-(2,4-disuccinoyl)-quinic acid, except for 4-nitrophenol and dicaffeoylquinic acid-O-glucoside, are all upward. Most of these metabolites are involved in the phenolic acid branch of phenylpropanoid metabolism and are further involved in flavonoid biosynthesis. Three coumarins 7-hydroxycoumarin-O-rhamnoside, 4-hydroxy-7-methoxycoumarin-β-rhamnoside, scopoletin-7-O-glucuronide, which showed significant changes; 10 flavonoids, namely pinocembrin, 4’,5-Dihydroxy-3’, 6,7-trimethoxyflavone, tricetin, isohyperoside, quercetin-3-O-galactoside, quercetin-7-O-glucoside, quercetin-3-O-glucuronide, Isorhamnetin-3,7-O-diglucoside, formononetin, ononin, sesquiterpenoids, reynosin, and 5α-hydroxycostic acid all showed an upward trend. Four substances were adjusted downward, among which pinocembrin, 4’,5-dihydroxy-3’,6,7-trimethoxyflavone belonged to dihydroflavones, and formononetin, ononin belonged to isoflavones, while the rest of flavonoids and flavonols were adjusted upward. The massive accumulation of these flavonoids in snowdrop leaves may act as endogenous antioxidants in the plant defense mechanism under low temperature stress. Twenty metabolites of this pathway were identified, and in addition to some of the above metabolites there wereidentified, including quinic acid, protocatechuic acid, shikimic acid, phenylalanine, 4-hydroxybenzoic acid, salicylic acid, cinnamic acid, coniferyl alcohol, pinoresinol, prunetin, esculetin, coumarin, apigenin, salicylacetic acid, protocatechuic acid, dihydrochrysin. These metabolites were upregulated except for Prunetin, which was downregulated in four groups, Phenylalanine, which was downregulated in CK vs. T1 and CK vs. T2, and Pinoresinol, which was downregulated in CK vs. T2, CK vs. T3, and CK vs. T4. In the comparison group of T1 vs. T3, T2 vs. T3 and T2 vs. T4, these substances were in an upward trend as a whole. Phenylalanine and sinapinaldehyde declined in all three comparison groups. It indicates that more kinds of phenolics can be produced under freezing conditions at -13 °C than at -4 °C, and more phenolics can be produced after cold adaptation.

**Table 1:** The amount of differential metabolites in *Sik*. leaves under low temperature stress

Group Class	CK/T1		CK/T2		CK/T3		CK/T4		T1/T3		T2/T4		T2/T3	
	Up	Down	Up	Down	Up	Down	Up	Down	Up	Down	Up	Down	Up	Down
Amino acids and derivatives	7	5	5	5	5	4	15	5	2	4	13	0	4	2
Phenolic acids	32	9	25	5	13	9	30	12	8	22	9	6	8	13
Nucleotides and derivatives	12	3	12	0	11	3	9	2	3	2	10	1	2	7
Flavonoids	31	16	11	8	22	11	33	14	8	15	13	11	9	8
Lignans and Coumarins	6	2	7	1	5	3	6	1	1	5	2	1	2	3
Alkaloids	4	11	5	3	5	4	3	9	2	3	3	6	3	5
Terpenoids	4	0	4	0	4	0	4	2	0	1	0	1	1	2
Organic acids	8	6	4	0	4	3	8	4	2	3	4	2	1	5
Lipids	37	5	58	2	54	11	47	10	26	6	9	63	6	26
Others	22	1	15	1	12	3	18	8	1	13	4	7	0	8
Total	163	58	146	25	135	51	173	67	53	74	67	98	36	79

#### Low-Temperature-Induced Changes in Purine Metabolism Pathway

Nucleotides are nucleic acid hydrolysates, and in this study, nucleotides and their derivatives were identified in the four comparison groups: guanine 5-aminoimidazole ribonucleotide, 2’-deoxyinosine-5’-monophosphate, guanosine 3’,5’-cyclic monophosphate, and uridine 5’-diphosphate both are up-regulated. These metabolites are all involved in the purine degradation pathway. We identified 9 nucleotides and their derivatives with significant low-temperature-induced accumulation in *Sik*. leaves, including Guanosine, Guanine, Hypoxanthine, Xanthine, Adenosine, Guanosine 3’,5’-cyclic monophosphate, Adenosine 5’-diphosphate, Cyclic 3’,5’-Adenylic acid、2’-Deoxyadenosine.

Guanosine, guanine, and hypoxanthine showed a significant up-regulation trend under low-temperature stress. Compared with the CK group guanine significantly increased in all four groups of low temperature stress, while Guanosine, Hypoxanthine showed a significant increase in the T2 group. Xanthine, adenosine showed an overall down-regulation trend, adenosine significantly decreased in the T3 group. Interestingly, xanthine in T3 group was upregulated and adenosine was upregulated in the T4 group. The intermediate adenosomes guanosine 3’,5’-cyclic monophosphate, adenosine 5’-diphosphate showed significant accumulation in all four comparison groups, cyclic 3’,5’-adenylic acid, 2’-deoxyadenosine showed an overall tendency to decrease. Although the accumulation of intermediate glands was obvious but the end products xanthine and adenosine were significantly reduced, only guanine showed a significant accumulation. The identified nucleotides and their derivatives were upregulated in all three comparison groups except adenosine 5’-diphosphate which was downregulated in T1 vs. T3, T2 vs. T3, T2 vs. T4 groups, guanine and xanthine which were downregulated in T1 vs. T3 and T2 vs. T3 and hypoxanthine which was downregulated in T1 vs. T3. Significant accumulation was observed in the T2 vs. T4 group except for adenosine 5’-diphosphate.

#### Low-Temperature-Induced glycolysis and TCA metabolic pathway

We identified six metabolites associated with glycolysis and TCA metabolic pathways, including D-glucose, D-glucose-6-phosphate, D-fructose-6-phosphate, succinic acid, oxaloacetate, and isocitric acid. Compared with the CK group, except for oxaloacetate in T2 and T3, the other metabolites were on the rise. In the pentose phosphate pathway, ribulose, ribulose-5-phosphate and erythritrose-4-phosphate were significantly accumulated in the four groups. In the synthesis pathway of sucrose and trehalose, the intermediate products D-lactose-6-phosphate and trehalose-6-phosphate were significantly accumulated, and trehalose was in an upward regulation trend except for the T3 group, and there was no significant change in sucrose. In the T1vsT3 group all nine substances were upregulated but in the T2 vs. T4 group all nine substances were downregulated. In the T2 vs. T3 group, only trehalose, trehalose-6-phosphate, fructose and glucose contents were increased, while the rest of substances were down-regulated or changed insignificantly. It indicates that soluble sugars can be accumulated more after low temperature conditions and cold adaptation.

#### Low-temperature-induced changes in amino acids and their derivatives

Under low temperature stress, only one amino acid analog, N-acetyl-L-aspartic Acid, was detected to be significantly up-regulated in all four groups compared to the control group.In addition, 12 amino acids and their derivatives were identified. L-cysteine, L-tryptophan and L-asparagine were in a downward adjustment trend in the four groups, while L-tryosine, L-phenylalanine, L-isoleucine and L-leucine decreased in the first two groups and showed an upward trend in the latter two groups. L-serine was down-regulated in the T2 group and up-regulated in the rest of the group. L-methionine was upregulated except for T1, L-ornothine was significantly downregulated in T4, and the rest were upregulated. The remaining amino acids L-lysine, L-proline, and L-grotamine were all upregulated in the four groups. The amino acids detected in the T1 vs. T3, T2 vs. T3, and T2 vs. T4 groups were in a downward trend as a whole, only ornithine increased in the three comparison groups, asparagine, cysteine accumulate in group T2 vs. T4, and proline was upregulated in T1 vs. T3, indicating that more amino acids could be accumulated after cold adaptation.

### Comprehensive metabolic network analysis under low temperature stress

**Fig. 6:**
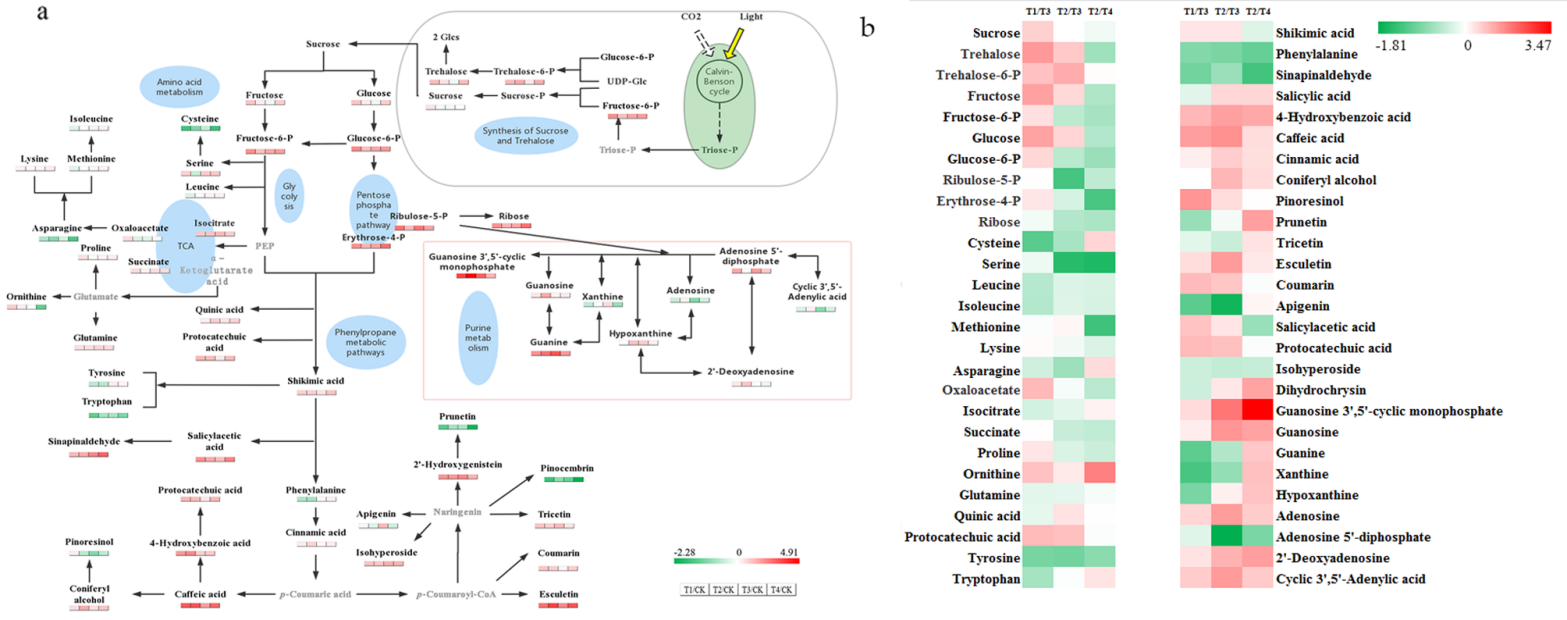
Metabolic network and metabolite analysis of *Sik*. leaves under low temperature stress. (**a**) CK vs. T1/ T2/ T3/T4 metabolic networks; (**b**) T1 vs. T3, T2 vs. T4, T2 vs. T3 changes in metabolites. Proposed metabolic pathways were based on the literature and web-based database of metabolic pathways. Metabolites that were not detected in this study but are important in the metabolic pathway are written in gray. The differential metabolite changes were represented by the log_2_ ratio. Green represents a decrease in content and red represents an increase in content

In order to get a comprehensive understanding of the changes in metabolites under different low temperature stress treatments, we proposed a metabolic pathway based on the literature on metabolic pathways and web databases. The main known pathways include sucrose and alginose synthesis, glycolysis, pentose phosphate pathway, tricarboxylic acid (TCA) cycle, amino acid metabolism, purine metabolism, and phenol metabolism (Fig. 6a-b).

## Discussion

The cytoplasmic membrane can act as a barrier against extracellular ice transmission, and the first physical change in plants at low temperatures is that of the cell membrane [31]. When subjected to cold stress, the fluidity and structure of membrane proteins change, leading to ion imbalance, metabolic disorders, and even death [3].Phosphatidylcholine (PC) is a class of lipids of biological importance and is the main structural component of cell membranes. Hydrolysis of one of PC’s fatty acid esters produces lysophosphatidylcholine (LPC), a key biomarker during cold damage to plants [32, 33]. Sweet pepper was found to suffer severe disruption of the cell membrane at 4 °C, and it was also confirmed that the PLD pathway is activated earlier and plays a dominant role during cold stress. In addition, the SFR2 pathway may mediate the remodeling of the chloroplast envelope under low temperature stress [34]. A large number of reactive oxygen species free radicals (ROS) are produced in the cells of maize seedlings under low temperature stress, and the increase of free radicals first attacks the membrane system, and the unsaturated bonds of membrane lipid fatty acids are peroxidized, resulting in membrane lipid peroxidation, resulting in an increase in malondialdehyde (MDA) content, resulting in damage to the cell membrane. But after cold adaptation, the newly accumulated peroxides in turn trigger a mechanism that increases the activity of several enzymes (Ascorbate peroxidase or Catalase), giving plants a high level of adaptation [35]. In this study, a large number of hemolytic ethanolamine components were detected in the four groups compared with the CK group, indicating that low temperature damaged the integrity of the membrane, while the significant decrease in T2 vs. T4 content indicated that the damage of the membrane after cold adaptation was reduced and the ability to resist adversity was increased, which was consistent with previous studies [35]. The decrease in the T2 vs. T3 group indicates that the degree of membrane damage is related to temperature, and the lower the temperature, the higher the degree of membrane damage. Stress disrupts the balance of light energy absorption and utilization, leading to the accumulation of reactive oxygen species, which causes oxidative damage to cells. Activating the plant antioxidant system can scavenge reactive oxygen species, reduce damage caused by lipid peroxidation of cell membranes, and maintain cell membrane stability [36]. In the results of our study, glutamine accumulated significantly in all four groups compared to CK. The accumulation of proline in the T1, T3 and T4 groups is obviously consistent with previous studies [25], while there is almost no change in the T2 group, indicating that the plant proline pathway can be stimulated to resist adversity stress under low temperature conditions, but in the freezing stress T2 group (-13°) plant cells may be severely damaged, leading to paralysis of metabolic pathways, but after cold adaptation, proline still accumulates, indicating that the cold tolerance of *Sik*. is enhanced after cold adaptation and is likely to improve the cold tolerance of plants through proline accumulation. Phenylalanine is a precursor to many key secondary metabolic pathways, such as flavonoids and anthocyanins, which influence cell osmotic regulation and improve cold tolerance in plants. The most abundant “L-phenylalanine catabolic process” in the category “Biological processes” in rice under low temperature stress conditions [37]. We investigated the accumulation of phenylalanine in snowdrop leaves induced by low-temperature stress. Phenylalanine content decreased in CK vs. T1 and CK vs. T2 but phenylpropanoids showed an increasing trend in all groups. Fewer phenylalanines were accumulated in the cold adaptation group compared to the T2 group, indicating enhanced degradation of phenylalanines after cold adaptation. This is consistent with previous studies [37]. In T1 vs. T3, T2 vs. T3, and T2 vs. T4 were in an overall decreasing trend in amino acid accumulation, indicating that amino acids were preferentially accumulated under freezing conditions to cope with external changes, while the ability to accumulate amino acids was enhanced after cold adaptation. These indicate that there are different patterns of metabolite accumulation under cold temperature, freezing, and cold adaptation conditions. This is the same as the classical data known decades ago.[38]. Soluble sugars have been shown to play an important role in cold acclimation [39]. At low temperatures, trehalose can be used as a carbon storage source and energy supply substance in organisms [40], and in some organisms, trehalose’s more important function is as a stress metabolite, and many biological species that show strong tolerance in adverse environments such as dry dehydration, high temperature, freezing, hyperosmolarity, etc., are directly related to the synthesis and accumulation of large amounts of trehalose in their bodies [41]. Some studies have concluded that trehalose is one of the most important permeates in fungal stress resistance [42]. Habibur studies have shown that trehalose 6-phosphatase (TPP) and trehalose are briefly highly induced under low temperature and cold stress in rice, suggesting that transient activation of trehalose biosynthesis is involved in early cryogenic stress responses in rice [43]. A study on the effect of trehalose on *Catharanthus roseus* under low temperature stress, and found that β-phenylalanine, quercetin, apigenin, chryxin and genistein were significantly accumulated under low temperature stress, while the accumulation decreased after exogenous trehalose. These findings reveal a positive regulatory effect of trehalose on the metabolism of C. roseus alkaloids [44]. In this study, trehalose, sucrose, glucose, fructose have accumulated, in the glycolytic pathway, compared with the control group, glucose-6-phosphate and fructose hexaphosphate are significantly upregulated, acetone phosphate is converted into acetyl-CoA and mengzu ester acetyl-CoA, respectively, into the tricarboxylic acid cycle, the metabolites participating in the TCA cycle are, oxaloacetate isocitric acid and succinic acid, wherein isocitric acid is significantly increased under low temperature stress, and the efficiency of glycolysis process is improved. More ATP can be generated as an energy source for other metabolic processes. And the content of these metabolites increased further after cold adaptation, which demonstrated the key role of soluble sugars in the resistance of *Sik*. to cryogenic stress. There was significant accumulation of erythritrose-4-phosphate, ribose-5-phosphate, and ribose in the pentose phosphate pathway. Pentose erythritrose-4-phosphate and phosphoenol pyruvate can synthesize shikimic acid, which is the predecessor of polyphenolic substances with antistress effects. In this experiment, the metabolites of phenylalanine metabolic pathway changed significantly in *Sik*. leaves after low temperature stress. Flavonoids are a major secondary metabolite in plants that perform a variety of functions in plant development and in response to biotic and abiotic stresses [45]. Zeaxanthin, sugar, and phenols in leaves of *Prunus avium* flowers increased under refrigerated conditions of 4.7° and 2.2°, especially zeaxanthin [46]. Expression of flavonol biosynthesis-related genes and flavonoid content in Tetrastigma hemsleyanum Diels et Gilg increased under low temperature stress [47]. In addition, flavonoids have accumulated in a variety of plants under cold stress, such as *Capsicum* [48], *Citrus sinensis* [49], *Medicago sativa* L. [50], *Brassica carinata* [51], etc. In this study, most of the substances in the phenylpropane synthesis pathway changed, for example, most substances showed significant accumulation such as caffeic acid, salicylacetic acid, esculetin, isohyperoside, but these phenolic substances declined after cold adaptation.

Changes in purine substances at low temperatures have been less studied, but in this study significant changes in purine metabolism were observed in snowdrop leaves at low temperatures. Although the accumulation of intermediate adenosine is high, the three final products hypoxanthine, xanthine and adenosine are significantly reduced, and only guanine has obvious accumulation, which means that the additional degradation of hypoxanthine, xanthine and adenosine may be a strategy for *Sik*. to resist low temperature.

## Materials and methods

### Plant material

*Saussurea involucrata* seeds are preserved by Key Laboratory of Xinjiang Phytomedicine Resources and Utilization, Ministry of Education, College of Life Sciences. *Sik*. seeds were cultured at room temperature conditions (20 °, 4800 lx, humidity 50%, day 16/night 8) in sterile medium, and after the seedlings grew to two months, good growth and consistent size of *Sik.* were selected for low temperature stress treatment, the treatment methods were as follows: 4 ° (24 h) in T1 group, -13 ° (3 h) in T2 group, -4 ° (12 h) in T3 group, 20 ° → 4 ° (12 h)→ 0 ° (6 h)→ -4 ° (3 h) → -13 ° (3 h) in T4 group. Each sample was repeated three times, with CK (20 °) samples as controls, for a total of 15 samples.

### Sample preparation

Biological samples are freeze-dried by vacuum freeze-dryer (Scientz-100 F). The freeze-dried sample was crushed using a mixer mill (MM 400, Retsch) with a zirconia bead for 1.5 min at 30 Hz. Dissolve 100 mg of lyophilized powder with 1.2 mL 70% methanol solution, vortex 30 s every 30 min for 6 times in total, place the sample in a refrigerator at 4 °C overnight. Following centrifugation at 12,000 rpm for 10 min, the extracts were filtrated (SCAA-104, 0.22 μm pore size; ANPEL,Shanghai, China, http://www.anpel.com.cn/) before UPLC-MS/MS analysis.

### UPLC conditions

The sample extracts were analyzed using an UPLC-ESI-MS/MS system (UPLC, SHIMADZU Nexera X2, https://www.shimadzu.com.cn/; MS, Applied Biosystems 4500 Q TRAP, https://www.thermofisher.cn/cn/zh/home/brands/applied-biosystems.html).

### ESI-Q TRAP-MS/MS

Biological samples are freeze-dried by vacuum freeze-dryer (Scientz-100 F).The freeze-dried sample waLIT and triple quadrupole (QQQ) scans were acquired on a triple quadrupole-linear ion trap mass spectrometer (Q TRAP), AB4500 Q TRAP UPLC/MS/MS System, equipped with an ESI Turbo Ion-Spray interface, operating in positive and negative ion mode and controlled by Analyst 1.6.3 software (AB Sciex). QQQ scans were acquired as MRM experiments with collision gas (nitrogen) set to medium. DP and CE for individual MRM transitions was done with further DP and CE optimization. A specific set of MRM transitions were monitored for each period according to the metabolites eluted within this period.

### Qualitative and quantitative analysis of metabolites

Metabolites were qualitatively and quantitatively analyzed by mass spectrometry using a public metabolite database and the self-built MWDB. The detected metabolites were shown in the multi-reaction monitoring mode MRM metabolite detection mul-tipeak map.Each mass spectrum peak of different colors represents a detected metabolite. The characteristic ions of each metabolite were obtained by triple four-stage rod screening, the signal intensity of the characteristic ions was obtained in the detector, and MultiQuant software was used to integrate and correct the chromatographic peaks [51]. The peak area (area) of each chromatographic peak represents the relative content of the corresponding metabolite [52].

### Multivariate data analysis and statistical analysis

Raw data signals are processed using Analyst 1.6.3 software (AB Sciex, Framingham, Massachusetts, USA).Logarithmic conversion of the original abundance of metabolites to normalize the data and test the homogeneity of variance. As described earlier, use R (www.r-project.org, 8 March 2021). Principal component analysis (PCA), clustering, and orthogonal projection discriminant analysis (OPLS-DA) of potential structures. Variable importance projection (VIP) values for all metabolites in OPLS-DA are extracted using the first component. The prediction parameters of the OPLS-DA evaluation model are R^2^X, R^2^Y, and Q^2^, where R^2^X and R^2^Y represent the interpretation rate of the model to the X and Y matrix respectively, and Q^2^ indicates the prediction ability of the model.

### Differential metabolite analysis

Metabolites that met both of the following criteria were selected as differential metabolites between groups (CK vs. T1, CK vs. T2, CK vs. T3, CK vs. T4, T1 vs. T3, T2 vs. T4, T2 vs. T3): (i) high confidence (VIP ≥ 1) two comparisons; (ii) folding change ≥ 2 or folding change ≤ 0.5. VIP values containing score plots and permutation plots were generated using the R package, MetaboAnalystR. The data were log transformed (log_2_) and mean centering was performed before OPLS-DA. To avoid overfitting, a permutation test (200 permutations) was performed. Selected differential metabolites were further annotated in the KEGG com-pound database (http://www.kegg.jp/kegg/compound/, 28 November 2020). Annotated metabolites were then mapped to the KEGG pathway database (http://www.kegg.jp/kegg/ pathway .html, 28 November 2020) [53].

## Conclusions

Our study revealed different metabolite changes in the response of *Sik*. to low temperature stress under different conditions. A total of 753 metabolites were identified. KEGG enrichment showed that these differential metabolites were involved in the biosynthesis of secondary metabolites and the biosynthesis of amino acids and sugars. A comprehensive understanding of metabolite changes under different low-temperature stresses showed that the synthesis of sucrose and trehalose, glycolysis, tricarboxylic acid cycle, pentose phosphate pathway, glutamic acid-mediated proline biosynthesis, purine metabolism, amino acid metabolism and phenylpropane synthesis pathway were involved in the response to low temperature stress. The extent to which each metabolic pathway responds under different cold stress conditions varies. The metabolic network of *Sik*. leaves under low temperature stress was established through the enrichment of differential metabolites in metabolic pathways responding to low temperature in *Sik*. leaves. In this study, a comprehensive metabolic network is proposed based on literature and web-based databases. These results will help further understand the response of *Sik*. metabolites to low-temperature stress. However, changes in metabolites do not explain the exact mechanism of *Sik*. response to low temperature stress. More work is needed to characterize the biological functions of the proposed network, such as recognizing metabolite function and protein changes. Through these, we will further unravel the metabolic mechanism of *Sik*. response to low temperature stress.

## Electronic supplementary material

Below is the link to the electronic supplementary material.

Supplementary Material 1

## Data Availability

All data generated or analysed during this study are included in this published article and its Supplementary information files.
