# Supplementary material for: Metabolomics-based exploration the response mechanisms of Saussurea involucrata leaves under different levels of low temperature stress
Source: BMC Genomics. 2023 Jun 1;24:297. doi: 10.1186/s12864-023-09376-4 (PMC10236807; doi:10.1186/s12864-023-09376-4)
Supplement: Supplementary file 1 — Supplementary Material 1 [file 12864_2023_9376_MOESM1_ESM.pdf]

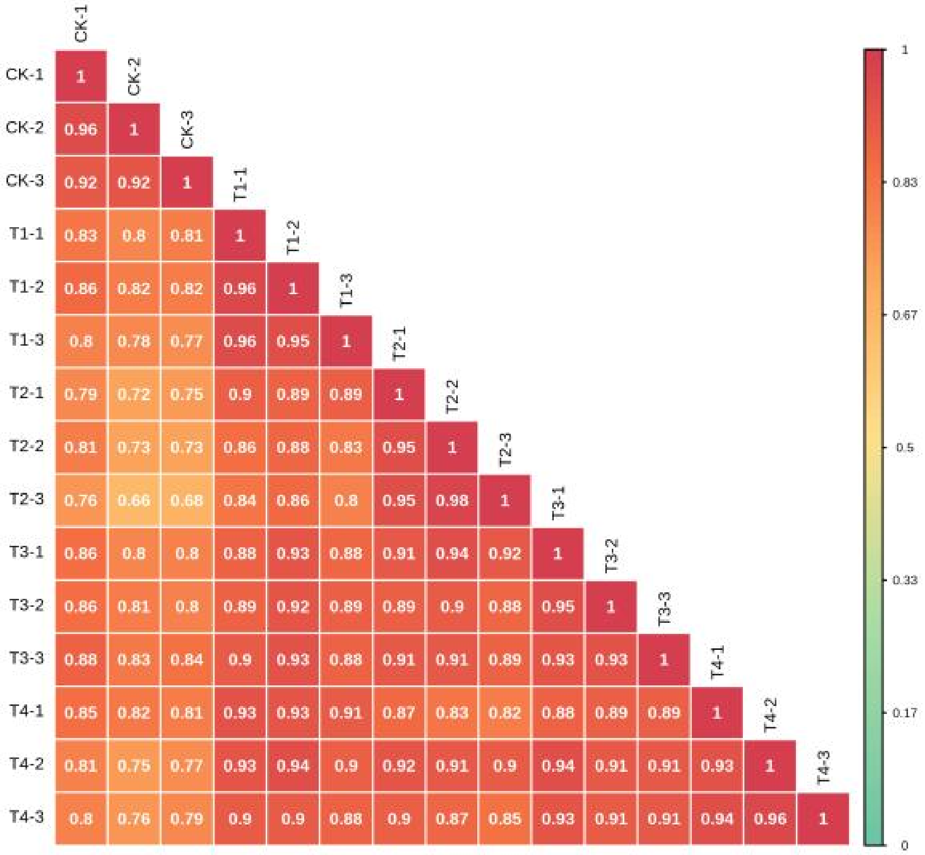

Figure S1. Different groups of Pearson's correlation coefficient diagram. Two repeated sample correlations were shown.

CK vs T1

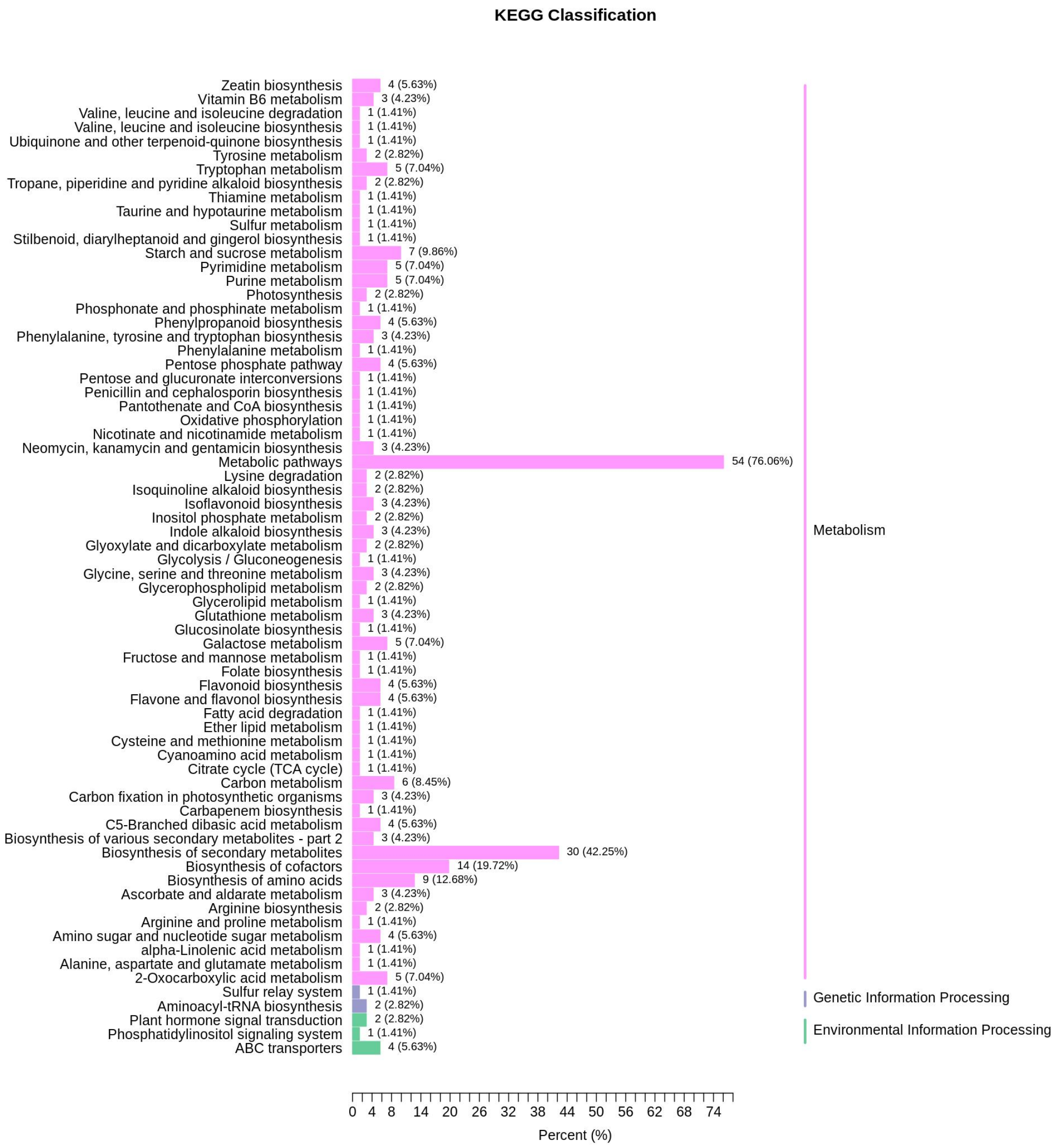
KEGG Classification
Zeatin biosynthesis 4 (5.63%)
Vitamin B6 metabolism 3 (4.23%)
Valine, leucine and isoleucine degradation 1 (1.41%)
Valine, leucine and isoleucine biosynthesis 1 (1.41%)
Ubiquinone and other terpenoid-quinone biosynthesis 1 (1.41%)
Tyrosine metabolism 2 (2.82%)
Tryptophan metabolism 5 (7.04%)
Tropane, piperidine and pyridine alkaloid biosynthesis 2 (2.82%)
Thiamine metabolism 1 (1.41%)
Taurine and hypotaurine metabolism 1 (1.41%)
Sulfur metabolism 1 (1.41%)
Stilbenoid, diarylheptanoid and gingerol biosynthesis 1 (1.41%)
Starch and sucrose metabolism 7 (9.86%)
Pyrimidine metabolism 5 (7.04%)
Purine metabolism 5 (7.04%)
Photosynthesis 2 (2.82%)
Phosphonate and phosphinate metabolism 1 (1.41%)
Phenylpropanoid biosynthesis 4 (5.63%)
Phenylalanine, tyrosine and tryptophan biosynthesis 3 (4.23%)
Phenylalanine metabolism 1 (1.41%)
Pentose phosphate pathway 4 (5.63%)
Pentose and glucuronate interconversions 1 (1.41%)
Penicillin and cephalosporin biosynthesis 1 (1.41%)
Pantothenate and CoA biosynthesis 1 (1.41%)
Oxidative phosphorylation 1 (1.41%)
Nicotinate and nicotinamide metabolism 1 (1.41%)
Neomycin, kanamycin and gentamicin biosynthesis 3 (4.23%)
Metabolic pathways 54 (76.06%)
Lysine degradation 2 (2.82%)
Isoquinoline alkaloid biosynthesis 2 (2.82%)
Isoflavonoid biosynthesis 3 (4.23%)
Inositol phosphate metabolism 2 (2.82%)
Indole alkaloid biosynthesis 3 (4.23%)
Glyoxylate and dicarboxylate metabolism 2 (2.82%)
Glycolysis / Gluconeogenesis 1 (1.41%)
Glycine, serine and threonine metabolism 3 (4.23%)
Glycerophospholipid metabolism 2 (2.82%)
Glycerolipid metabolism 1 (1.41%)
Glutathione metabolism 3 (4.23%)
Glucosinolate biosynthesis 1 (1.41%)
Galactose metabolism 5 (7.04%)
Fructose and mannose metabolism 1 (1.41%)
Folate biosynthesis 1 (1.41%)
Flavonoid biosynthesis 4 (5.63%)
Flavone and flavonol biosynthesis 4 (5.63%)
Fatty acid degradation 1 (1.41%)
Ether lipid metabolism 1 (1.41%)
Cysteine and methionine metabolism 1 (1.41%)
Cyanoamino acid metabolism 1 (1.41%)
Citrate cycle (TCA cycle) 1 (1.41%)
Carbon metabolism 6 (8.45%)
Carbon fixation in photosynthetic organisms 3 (4.23%)
Carbapenem biosynthesis 1 (1.41%)
C5-Branched dibasic acid metabolism 4 (5.63%)
Biosynthesis of various secondary metabolites - part 2 3 (4.23%)
Biosynthesis of secondary metabolites 30 (42.25%)
Biosynthesis of cofactors 14 (19.72%)
Biosynthesis of amino acids 9 (12.68%)
Ascorbate and aldarate metabolism 3 (4.23%)
Arginine biosynthesis 2 (2.82%)
Arginine and proline metabolism 1 (1.41%)
Amino sugar and nucleotide sugar metabolism 4 (5.63%)
alpha-Linolenic acid metabolism 1 (1.41%)
Alanine, aspartate and glutamate metabolism 1 (1.41%)
2-Oxocarboxylic acid metabolism 5 (7.04%)
Sulfur relay system 1 (1.41%)
Aminoacyl-tRNA biosynthesis 2 (2.82%)
Plant hormone signal transduction 2 (2.82%)
Phosphatidylinositol signaling system 1 (1.41%)
ABC transporters 4 (5.63%)
Metabolism
Genetic Information Processing
Environmental Information Processing
0 4 8 14 20 26 32 38 44 50 56 62 68 74
Percent (%)

CK vs T2

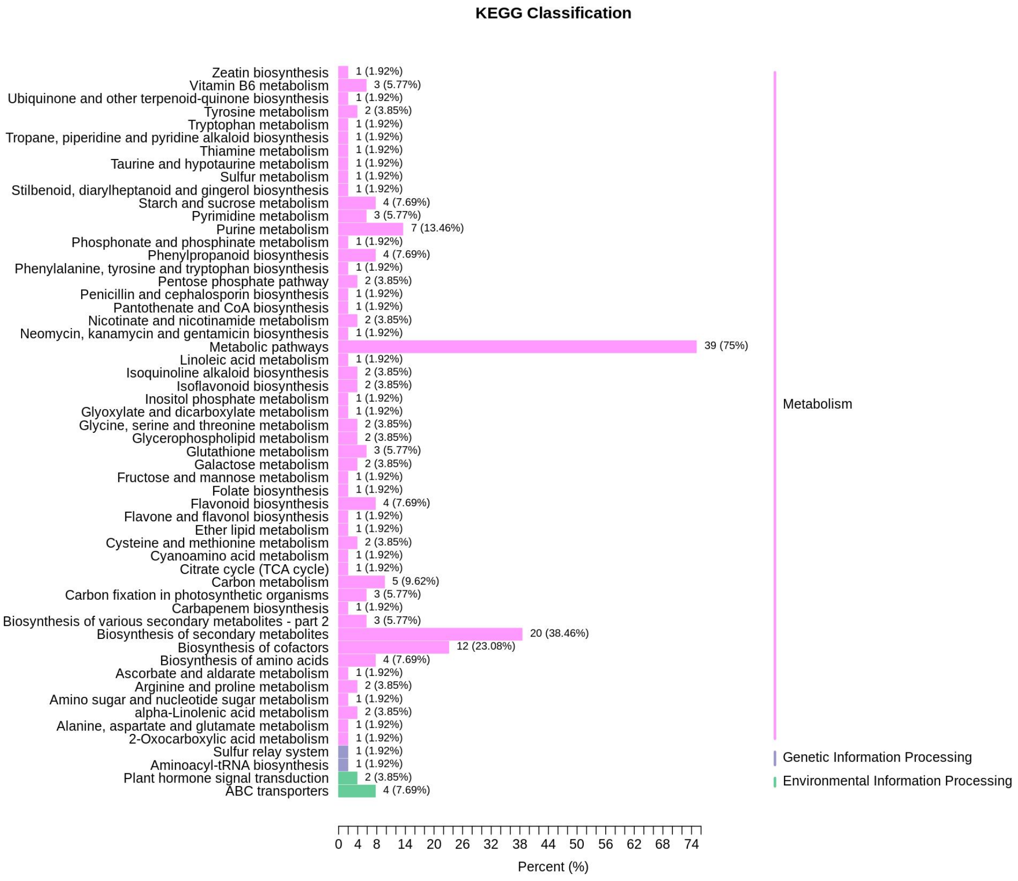

CK vs T3

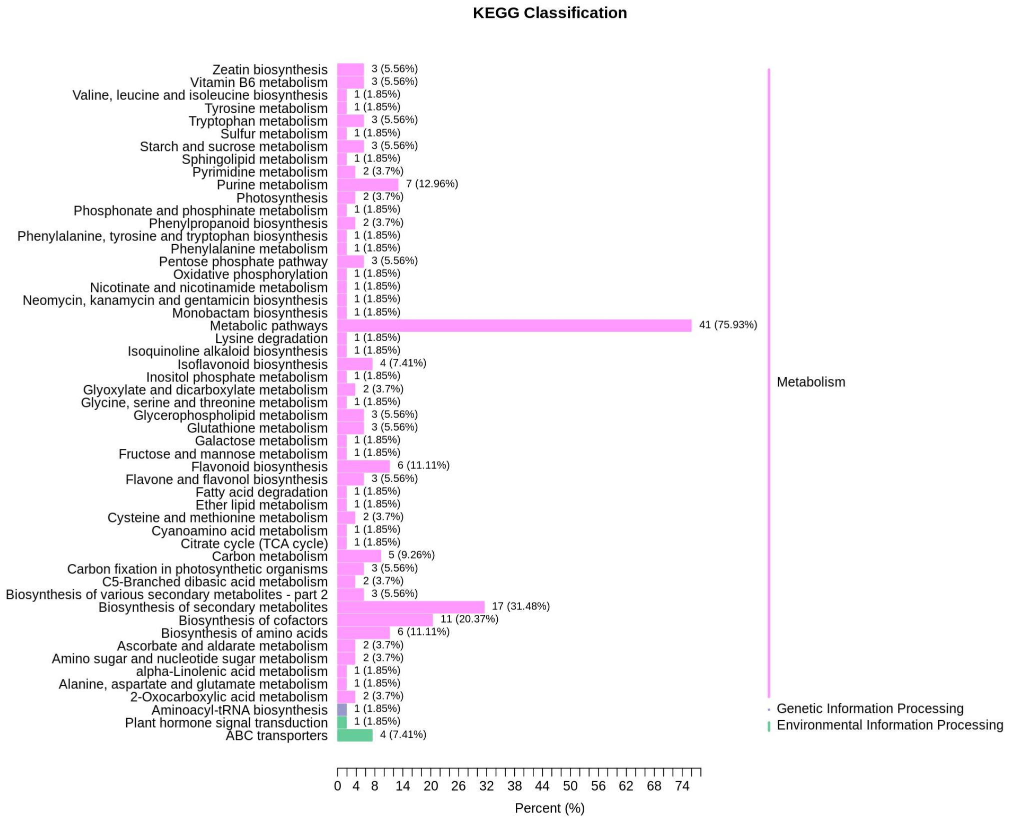

CK vs T4

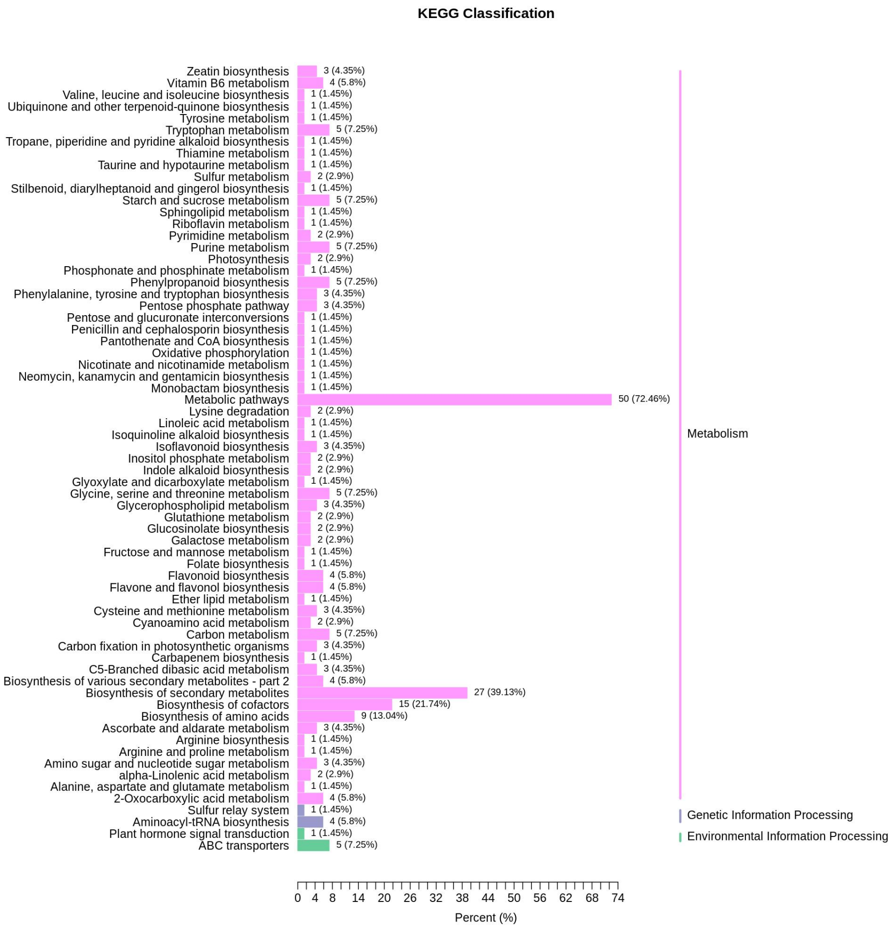

T1vsT3

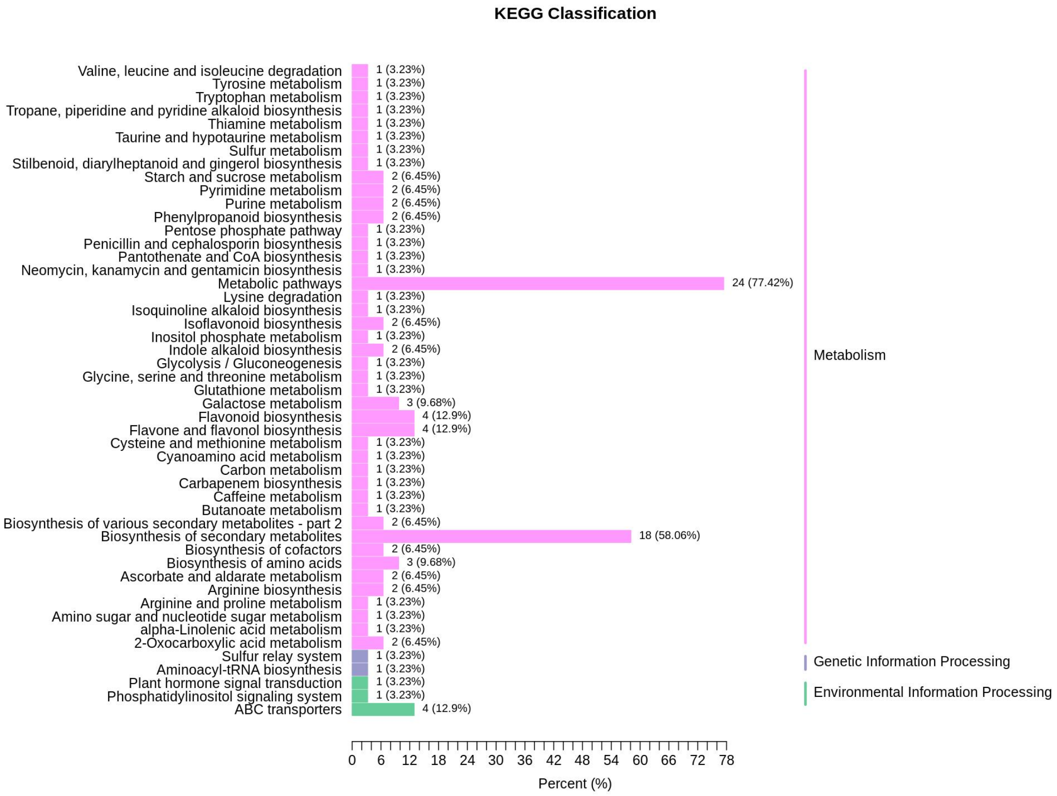

T2vsT3

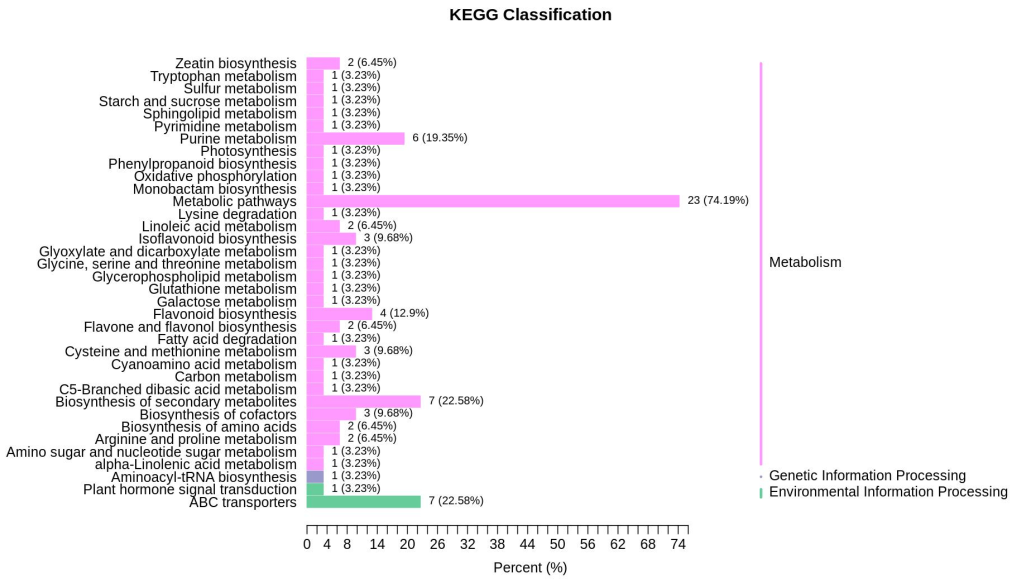

T2vsT4.

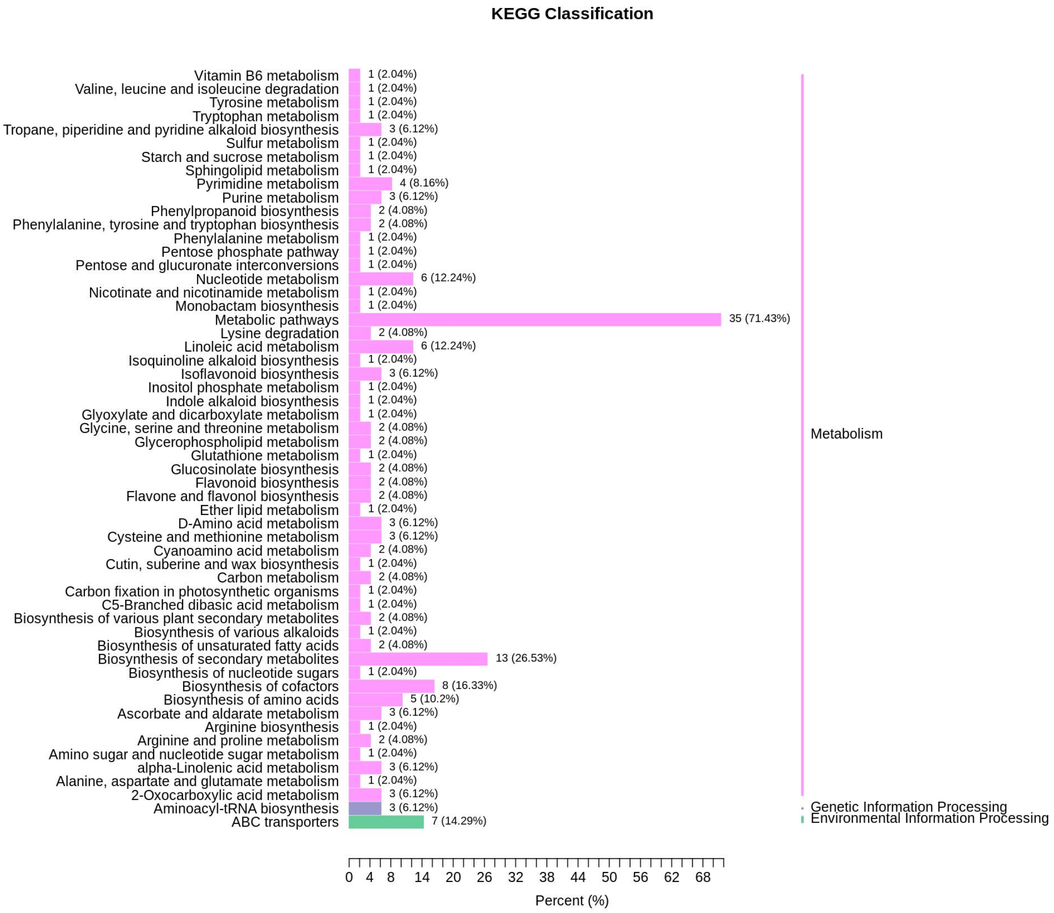

Figure S2. KEGG classification map of differential metabolites. CK vs T1; CK vs T2; CK vs T3; CKvsT4; T1vsT3; T2vsT3; T2vsT4. The ordinate is the name of the KEGG metabolic pathway, and the abscess is the number of metabolites annotated to the pathway and their proportion to the total number of annotated metabolites.

CK vs T1

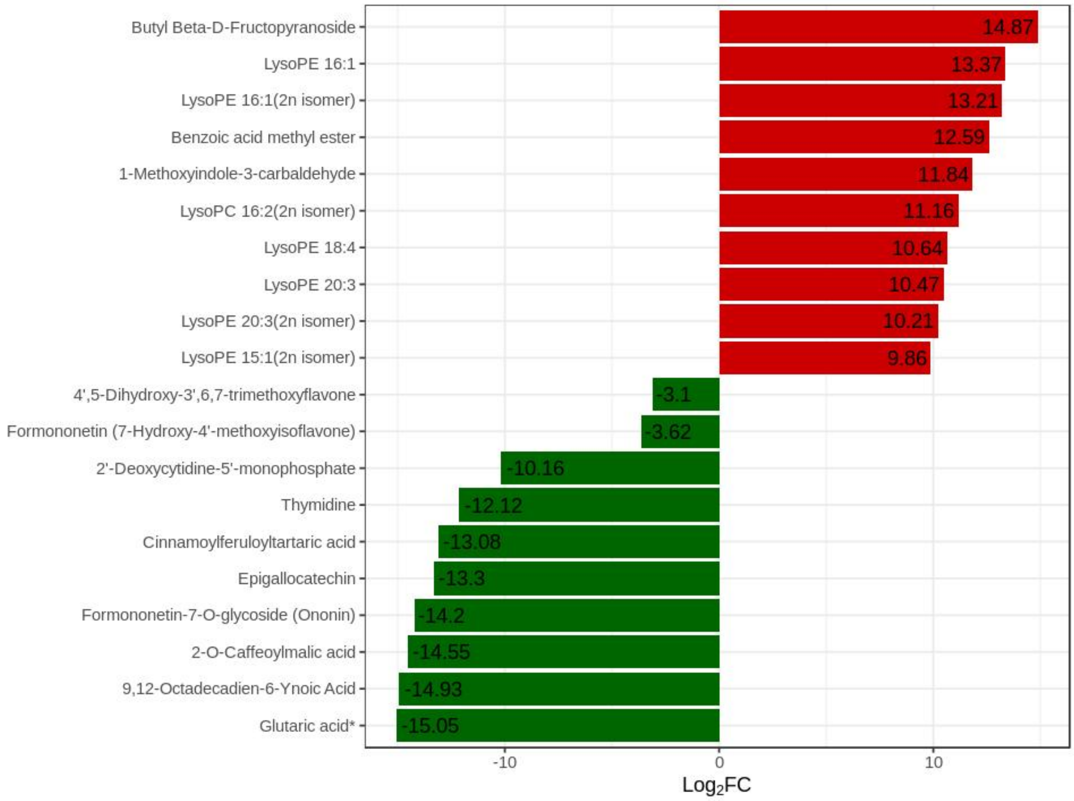

CK vs T2

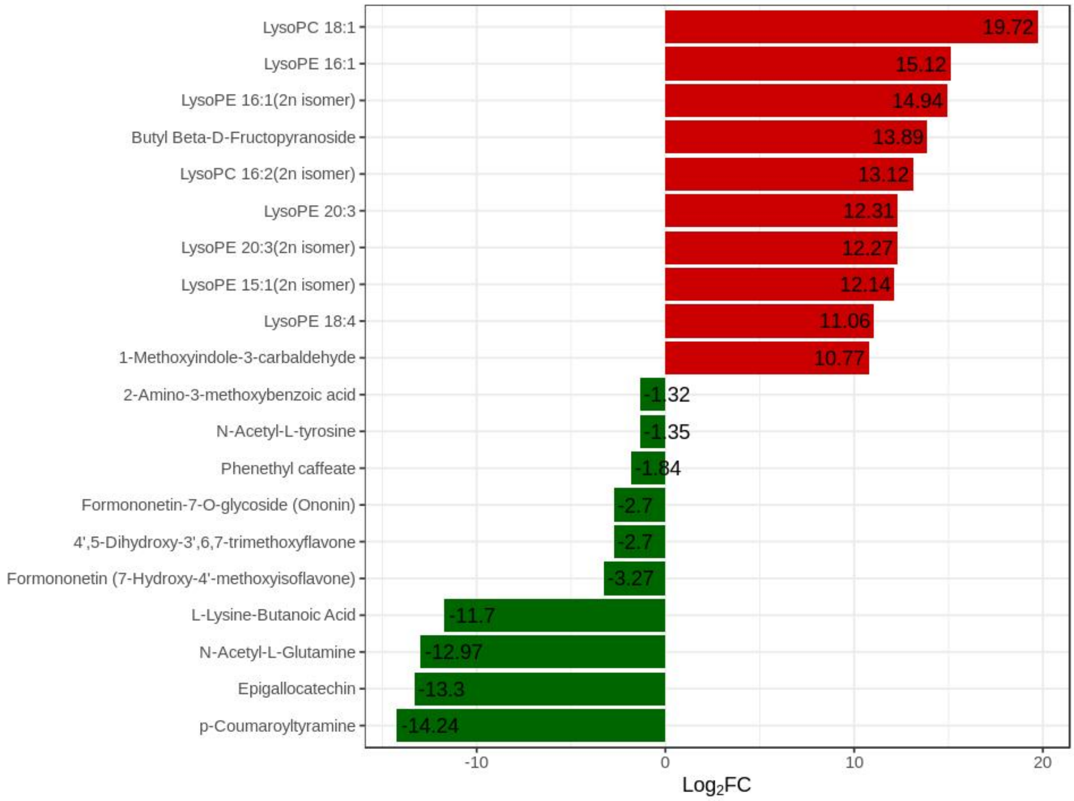

CK vs T3

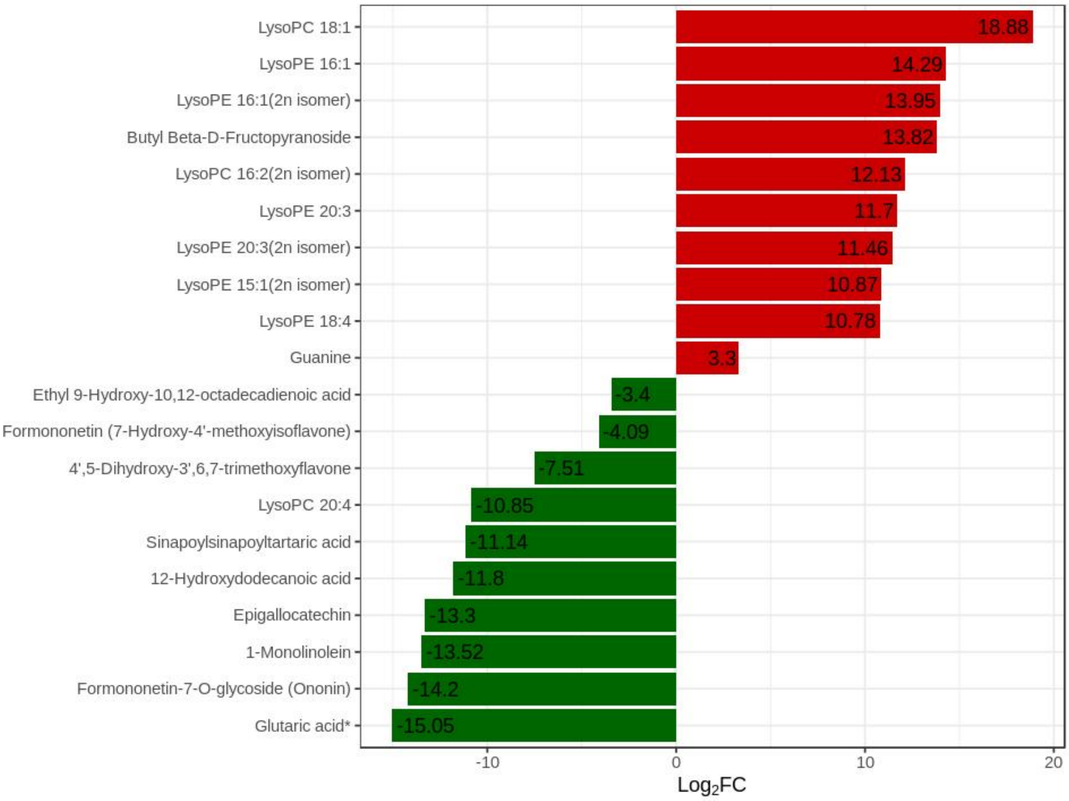

CK vs T4

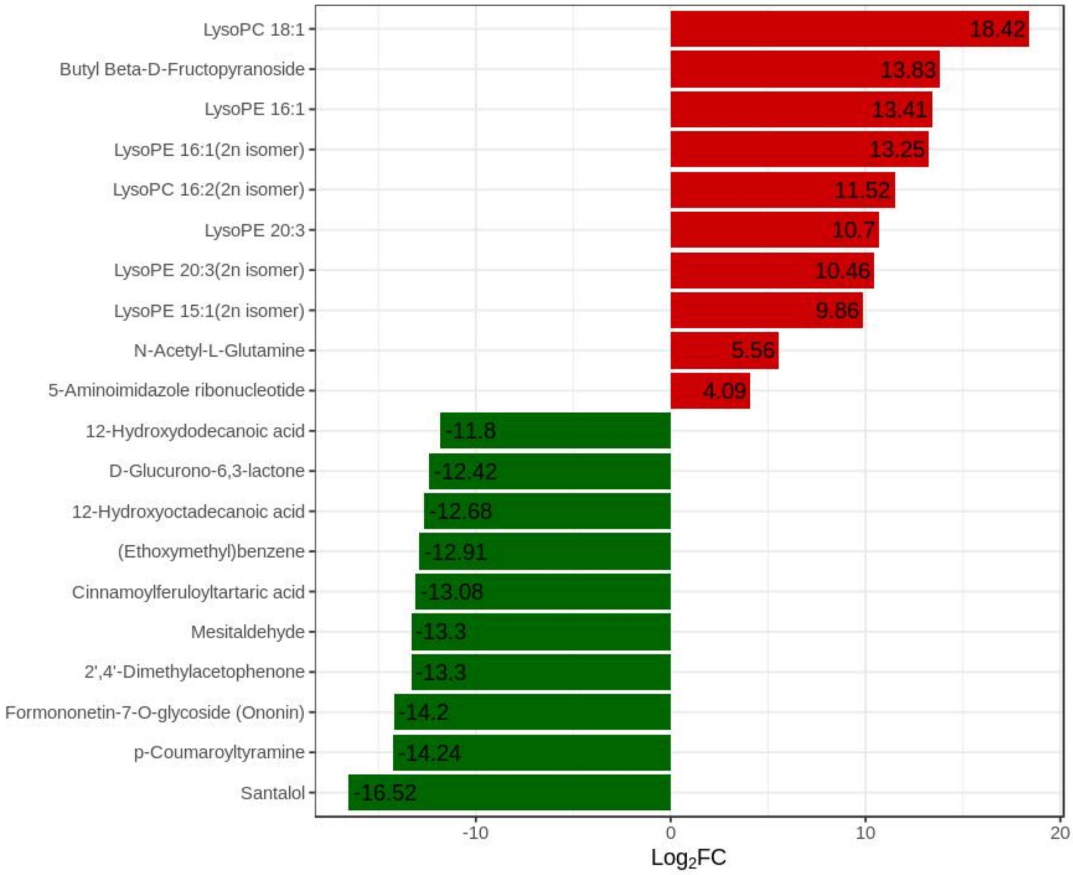

T1vsT3

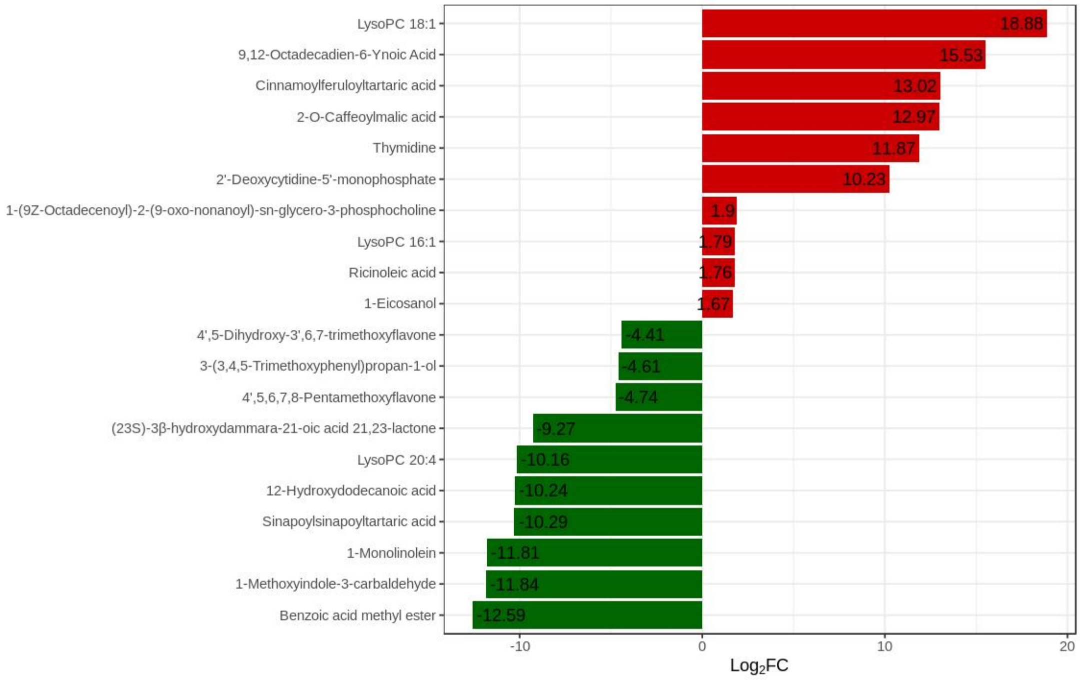

T2vsT3

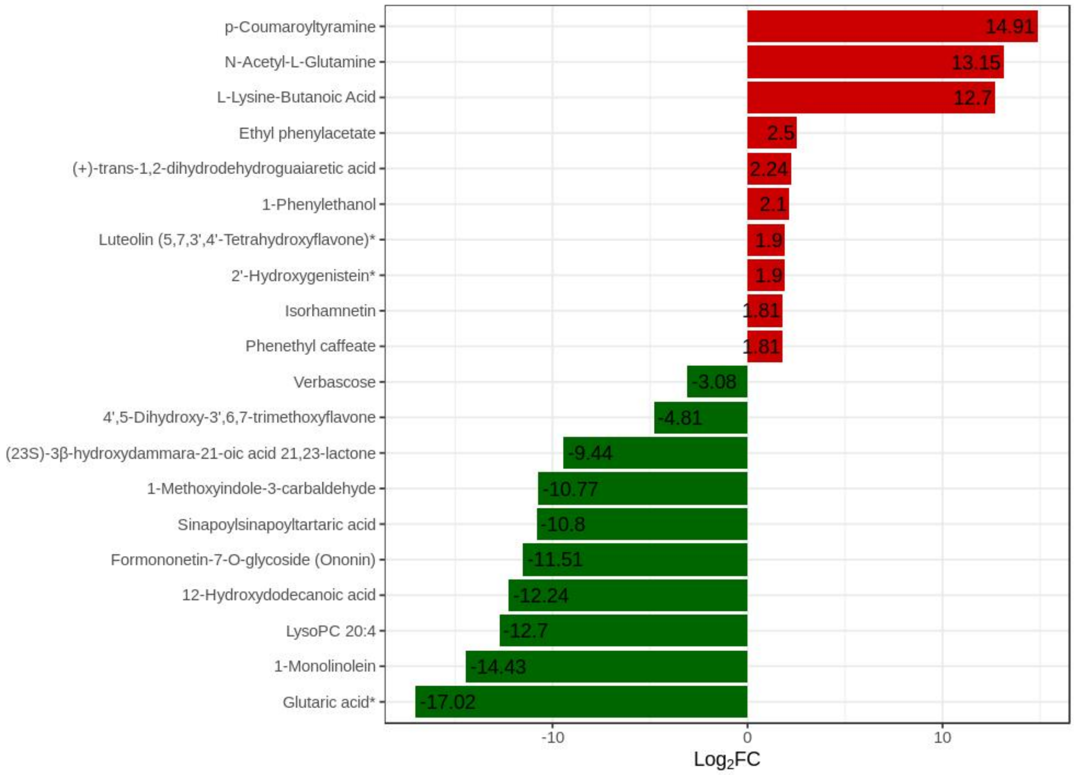

T2vsT4

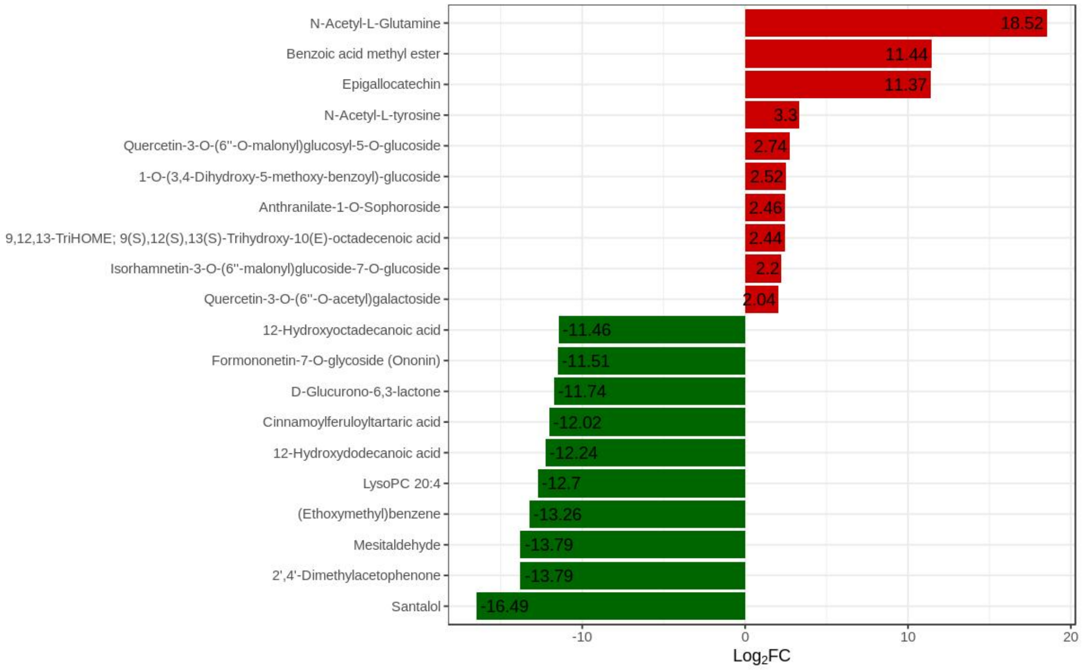

Figure S3. Bar chart of differential metabolites. CK vs T1; CK vs T2; CK vs T3; CKvsT4; T1vsT3; T2vsT3; T2vsT4. The top ten up-regulated metabolites and the top ten down-regulated metabolites of each comparison group were screened.

Table S1. The classification of compounds detected by the metabolome is in CK , T1, T2, T3 and T4.

Index	Q1 (Da)	Q3 (Da)	Molecular Weight (Da)	Formula	Ionization model	Class	Compounds
ML10198895	88.04	88.04	89.05	C3H7NO2	[M-H]-	Amino acids and derivatives	N-Methylglycine
pme2601	89.00	59.00	90.03	C3H6O3	[M-H]-	Organic acids	3-Hydroxypropanoic acid
Lmqp000873	101.02	55.02	100.02	C4H4O3	[M+H]+	Organic acids	Succinic anhydride
pme3017	104.07	58.00	103.06	C4H9NO2	[M+H]+	Organic acids	2-Aminoisobutyric acid
pme3033	104.07	86.00	103.06	C4H9NO2	[M+H]+	Amino acids and derivatives	N,N-Dimethylglycine
MWSmce585	104.07	59.01	103.06	C4H9NO2	[M+H]+	Amino acids and derivatives	Methyl 3-aminopropanoate
pmb0484	104.10	60.10	103.09	C5H13NO	[M+H]+	Alkaloids	Choline
ML10172161	103.00	59.01	104.01	C3H4O4	[M-H]-	Organic acids	Hydroxypyruvic acid
mws0576	103.04	59.10	104.05	C4H8O3	[M-H]-	Organic acids	3-Hydroxybutyric acid
pme1975	103.00	59.00	104.06	C3H4O4	[M-H]-	Organic acids	Malonic acid
pme0010	106.00	60.00	105.04	C3H7NO3	[M+H]+	Amino acids and derivatives	L-Serine
HJAP104	107.05	77.04	106.04	C7H6O	[M+H]+	Phenolic acids	Benzaldehyde
pme0006	116.07	70.10	115.06	C5H9NO2	[M+H]+	Amino acids and derivatives	L-Proline
pme3193	116.04	74.00	117.04	C4H7NO3	[M-H]-	Amino acids and derivatives	N-Acetyl-L-glycine
pmb1096	118.07	91.00	117.06	C8H7N	[M+H]+	Alkaloids	Indole
pme0120	118.09	101.00	117.08	C5H11NO2	[M+H]+	Organic acids	5-Aminovaleric acid
MWSmce548	118.09	58.07	117.08	C5H11NO2	[M+H]+	Alkaloids	Betaine
mws0192	117.02	73.00	118.03	C4H6O4	[M-H]-	Organic acids	Succinic acid*
mws0470	117.02	73.00	118.03	C4H6O4	[M-H]-	Organic acids	Methylmalonic acid*
mws0147	117.06	59.00	118.06	C5H10O3	[M-H]-	Organic acids	β-Hydroxyisovaleric acid
pme3096	118.00	74.00	119.02	C3H5NO4	[M-H]-	Organic acids	Aminomalonic acid
pmp001287	120.08	103.05	119.07	C8H9N	[M+H]+	Alkaloids	N-Benzylmethylene isomethylamine

Lmbp000728	121.06	77.04	120.06	C8H8O	[M+H]+	Others	(S)-2-Phenyloxirane
pme0195	120.02	62.00	121.02	C3H7NO2S	[M-H]-	Amino acids and derivatives	L-Cysteine
mws0628	121.03	92.00	122.04	C7H6O2	[M-H]-	Phenolic acids	4-Hydroxybenzaldehyde
mws0133	123.06	80.05	122.05	C6H6N2O	[M+H]+	Others	Nicotinamide
MWS2091	123.08	77.04	122.07	C8H10O	[M+H]+	Phenolic acids	1-Phenylethanol
pme0490	124.04	78.00	123.03	C6H5NO2	[M+H]+	Others	Nicotinic acid (Vitamin B3)
Lmbp000668	124.04	78.03	123.03	C6H5NO2	[M+H]+	Others	Isonicotinic acid
mws0572	126.07	109.00	125.06	C5H7N3O	[M+H]+	Nucleotides and derivatives	5-Methylcytosine
mws0982	126.10	68.00	125.10	C6H11N3	[M+H]+	Alkaloids	1-Methylhistamine
MWS0813	128.04	82.03	129.04	C5H7NO3	[M-H]-	Amino acids and derivatives	5-Oxoproline
Lmbn000612	128.04	82.03	129.04	C5H7NO3	[M-H]-	Organic acids	1-Pyrroline-4-hydroxy-2-carboxylic acid
MWSmce709	130.06	103.05	129.06	C9H7N	[M+H]+	Alkaloids	Isoquinoline
MWS0811	130.09	84.08	129.08	C6H11NO2	[M+H]+	Organic acids	L-Pipecolic Acid
pme2693	131.12	114.09	130.11	C6H14N2O	[M+H]+	Alkaloids	N-Acetylputrescine
Zmpn000638	130.05	88.04	131.07	C4H9N3O2	[M-H]-	Organic acids	3-Guanidinopropionic acid
mws0258	132.10	86.00	131.10	C6H13NO2	[M+H]+	Amino acids and derivatives	L-Isoleucine*
pme0274	132.10	69.00	131.10	C6H13NO2	[M+H]+	Organic acids	6-Aminocaproic acid
mws0227	132.10	86.20	131.10	C6H13NO2	[M+H]+	Amino acids and derivatives	L-Leucine*
mws1587	132.10	86.00	131.10	C6H13NO2	[M+H]+	Amino acids and derivatives	L-Norleucine*
mws1167	131.00	59.20	132.01	C4H4O5	[M-H]-	Organic acids	Oxaloacetic acid
mws0473	131.03	87.05	132.04	C5H8O4	[M-H]-	Organic acids	2-Methylsuccinic acid*
Lmmn000806	131.03	87.05	132.04	C5H8O4	[M-H]-	Organic acids	Dimethylmalonic acid*
pme0243	131.03	87.05	132.04	C5H8O4	[M-H]-	Organic acids	Glutaric acid*
Lmgn000160	131.05	70.03	132.05	C4H8N2O3	[M-H]-	Organic acids	3-Ureidopropionic Acid
mws0001	133.06	74.00	132.05	C4H8N2O3	[M+H]+	Amino acids and derivatives	L-Asparagine
pmc0682	133.10	105.10	132.07	C8H8N2	[M+H]+	Alkaloids	4-Aminoindole

Lmrn002746	131.07	85.07	132.08	C6H12O3	[M-H]-	Organic acids	2-Hydroxy-4-methylpentanoic acid
pme2527	133.00	116.00	132.09	C5H12N2O2	[M+H]+	Amino acids and derivatives	L-Ornithine
mws0219	132.03	88.00	133.04	C4H7NO4	[M-H]-	Amino acids and derivatives	L-Aspartic Acid*
MWS1882	132.03	88.04	133.04	C4H7NO4	[M-H]-	Organic acids	Iminodiacetic acid*
Lmbn000198	133.01	71.01	134.02	C4H6O5	[M-H]-	Others	3-Dehydro-L-Threonic Acid*
MWS1709	133.01	71.01	134.02	C4H6O5	[M-H]-	Organic acids	D-Malic acid*
Zmdp000972	136.04	91.05	135.04	C4H9NO2S	[M+H]+	Amino acids and derivatives	S-Methyl-L-cysteine
Lmgp000659	136.08	91.05	135.07	C8H9NO	[M+H]+	Alkaloids	2-Phenylacetamide*
HJKP000649	136.07	91.05	135.07	C8H9NO	[M+H]+	Alkaloids	N-benzylformamide*
mws0889	135.03	75.01	136.04	C4H8O5	[M-H]-	Others	D-Threonic Acid
pme0033	137.05	119.00	136.04	C5H4N4O	[M+H]+	Nucleotides and derivatives	Hypoxanthine
MWSmce466	135.05	92.03	136.05	C8H8O2	[M-H]-	Phenolic acids	4-Hydroxyacetophenone*
pme1724	137.06	91.00	136.05	C8H8O2	[M+H]+	Phenolic acids	Benzoic acid methyl ester
MWS1848	135.05	92.03	136.05	C8H8O2	[M-H]-	Phenolic acids	Phenyl acetate*
MWSmce699	137.10	91.05	136.09	C9H12O	[M+H]+	Others	(Ethoxymethyl)benzene
mws0146	138.06	78.00	137.05	C7H7NO2	[M+H]+	Alkaloids	Nicotinic Acid Methyl Ester(Methyl Nicotinate)
pme1002	138.09	103.00	137.08	C8H11NO	[M+H]+	Alkaloids	L-Tyramine
mws0749	137.02	93.00	138.03	C7H6O3	[M-H]-	Phenolic acids	4-Hydroxybenzoic acid*
Hmgn001653	137.02	93.04	138.03	C7H6O3	[M-H]-	Phenolic acids	Protocatechualdehyde*
Lmgn001670	137.03	108.02	138.03	C7H6O3	[M-H]-	Phenolic acids	Salicylic acid
mws2368	137.06	119.05	138.07	C8H10O2	[M-H]-	Phenolic acids	Tyrosol; 4-Hydroxyphenylethanol
pme2828	140.00	99.00	139.03	C6H5NO3	[M+H]+	Phenolic acids	4-Nitrophenol
pma2987	142.10	124.09	141.09	C6H11N3O	[M+H]+	Alkaloids	Histidinol
pme2879	141.00	123.00	142.04	C5H6N2O3	[M-H]-	Nucleotides and derivatives	5-Hydroxymethyluracil
Lmcp007199	143.09	128.06	142.08	C11H10	[M+H]+	Others	2-Methylnaphthalene
pmp000966	144.10	84.10	143.10	C7H13NO2	[M+H]+	Alkaloids	Stachydrine

Rfmb318	144.10	84.08	144.09	C7H14NO2+	[M]+	Amino acids and derivatives	D-Proline betaine
Lmyn008503	143.11	143.11	144.12	C8H16O2	[M-H]-	Lipids	Octanoic acid
mws0851	143.11	143.11	144.12	C8H16O2	[M-H]-	Organic acids	Valproic Acid
Qmkp093004	146.06	91.05	145.05	C9H7NO	[M+H]+	Alkaloids	Indole-3-carboxaldehyde
mws0567	146.09	87.00	145.09	C5H11N3O2	[M+H]+	Organic acids	4-Guanidinobutyric acid
HX1365	147.04	91.05	146.04	C9H6O2	[M+H]+	Lignans and Coumarins	Coumarin
Wmzn000227	145.06	109.04	146.06	C6H10O4	[M-H]-	Organic acids	2,2-Dimethylsuccinic acid
pme0193	147.08	84.00	146.07	C5H10N2O3	[M+H]+	Amino acids and derivatives	L-Glutamine
pme0026	147.11	84.00	146.11	C6H14N2O2	[M+H]+	Amino acids and derivatives	L-Lysine
Zmzn000113	146.05	102.06	147.05	C5H9NO4	[M-H]-	Amino acids and derivatives	L-threo-3-Methylaspartate
pmp001285	149.02	65.04	148.02	C8H4O3	[M+H]+	Phenolic acids	Phthalic anhydride
Lmbn000216	147.03	57.03	148.04	C5H8O5	[M-H]-	Organic acids	3-Methylmalic acid*
Zmyn000247	147.03	57.03	148.04	C5H8O5	[M-H]-	Organic acids	2-Hydroxyglutaric Acid*
Zmyn000230	147.03	57.03	148.04	C5H8O5	[M-H]-	Others	2-Dehydro-3-deoxy-L-arabinonate*
pmb2826	147.03	87.10	148.04	C5H8O5	[M-H]-	Organic acids	L-Citramalic acid
MWS20194	149.06	103.06	148.05	C9H8O2	[M+H]+	Phenolic acids	Cinnamic acid
MWSmce698	149.10	65.04	148.09	C10H12O	[M+H]+	Others	2',4'-Dimethylacetophenone
MWSmce463	149.10	65.04	148.09	C10H12O	[M+H]+	Others	Mesitaldehyde
Lmgp000796	150.06	61.01	149.05	C8H7NO2	[M+H]+	Alkaloids	4-Hydroxymandelonitrile
pme1210	150.06	61.00	149.05	C5H11NO2S	[M+H]+	Amino acids and derivatives	L-Methionine
mws1499	149.05	59.01	150.05	C5H10O5	[M-H]-	Others	D-Arabinose
Zmgn000173	149.05	75.01	150.05	C5H10O5	[M-H]-	Others	D-Ribose
pme1109	152.06	135.00	151.05	C5H5N5O	[M+H]+	Nucleotides and derivatives	Guanine
pme0256	151.03	151.00	152.03	C5H4N4O2	[M-H]-	Nucleotides and derivatives	Xanthine
mws0458	151.04	136.00	152.05	C8H8O3	[M-H]-	Phenolic acids	Vanillin
Zmyn002323	151.04	107.05	152.05	C8H8O3	[M-H]-	Organic acids	2-Hydroxyphenylacetic acid

mws0213	151.06	71.00	152.07	C5H12O5	[M-H]-	Others	Ribitol*
pme0513	151.06	59.00	152.07	C5H12O5	[M-H]-	Others	Xylitol
mws0437	151.06	71.00	152.07	C5H12O5	[M-H]-	Others	D-Arabitol*
mws0444	152.04	108.00	153.04	C7H7NO3	[M-H]-	Phenolic acids	3-Aminosalicylic acid
mws0183	153.02	109.03	154.03	C7H6O4	[M-H]-	Phenolic acids	3,4-Dihydroxybenzoic acid (Protocatechuic acid)*
mws0180	153.02	109.03	154.03	C7H6O4	[M-H]-	Phenolic acids	2,5-Dihydroxybenzoic acid; Gentisic Acid*
mws0254	156.08	110.00	155.07	C6H9N3O2	[M+H]+	Amino acids and derivatives	L-Histidine
pmb0819	157.00	130.00	156.07	C10H8N2	[M+H]+	Alkaloids	3-Indoleacetonitrile
Lmbn001467	158.08	116.07	159.09	C7H13NO3	[M-H]-	Organic acids	5-Acetamidopentanoic Acid
Lmbn002072	159.07	97.07	160.07	C7H12O4	[M-H]-	Organic acids	2-Propylsuccinic acid
mws0005	161.11	144.00	160.10	C10H12N2	[M+H]+	Alkaloids	Tryptamine
mws1417	160.04	116.00	161.05	C9H7NO2	[M-H]-	Alkaloids	Indole-3-carboxylic acid
Lmgp001898	162.06	116.05	161.05	C9H7NO2	[M+H]+	Alkaloids	4,6-Dihydroxyquinoline
pme3382	160.06	98.00	161.07	C6H11NO4	[M-H]-	Amino acids and derivatives	N-Acetyl-L-threonine
Hmqn002118	161.02	133.03	162.03	C9H6O3	[M-H]-	Lignans and Coumarins	Umbelliferone
pme2914	161.05	99.00	162.05	C6H10O5	[M-H]-	Amino acids and derivatives	3-Hydroxy-3-methylpentane-1,5-dioic acid
pmf0440	163.08	91.10	162.07	C10H10O2	[M+H]+	Phenolic acids	4-MethoxycinnaMaldehyde
pme2758	162.04	144.00	163.05	C5H9NO5	[M-H]-	Amino acids and derivatives	4-Hydroxy-L-glutamic acid
pme1419	164.07	104.00	163.07	C6H13NO2S	[M+H]+	Amino acids and derivatives	L-Methionine methyl ester
mws0159	163.04	91.00	164.05	C9H8O3	[M-H]-	Organic acids	Phenylpyruvic acid
pmb0142	165.10	95.20	164.05	C9H8O3	[M+H]+	Phenolic acids	Caffeic aldehyde
MWSmce712	165.09	91.05	164.08	C10H12O2	[M+H]+	Phenolic acids	Ethyl phenylacetate
Lmmn004032	163.11	148.06	164.12	C11H16O	[M-H]-	Others	Cis-Jasmone
pme3083	164.04	120.00	165.04	C8H7NO3	[M-H]-	Phenolic acids	2-(Formylamino)benzoic acid
pme2617	166.05	102.00	165.05	C5H11NO3S	[M+H]+	Amino acids and derivatives	L-Methionine Sulfoxide
pme0021	166.09	120.08	165.07	C9H11NO2	[M+H]+	Amino acids and derivatives	L-Phenylalanine

pme0282	165.02	77.04	166.03	C8H6O4	[M-H]-	Phenolic acids	Phthalic acid
pmc0274	167.10	121.10	166.03	C6H6N4S	[M+H]+	Nucleotides and derivatives	6-Methylmercaptopurine
mws0344	165.04	75.00	166.05	C5H10O6	[M-H]-	Others	D-Xylonic acid
Lmrn003000	165.06	103.06	166.06	C9H10O3	[M-H]-	Phenolic acids	2-Hydroxy-3-phenylpropanoic acid
Lmbn005172	165.06	119.05	166.06	C9H10O3	[M-H]-	Phenolic acids	2,6-Dimethoxybenzaldehyde*
MWS0274	165.06	119.05	166.06	C9H10O3	[M-H]-	Organic acids	DL-3-Phenyllactic acid*
MWSmce165	165.08	59.02	166.08	C6H14O5	[M-H]-	Others	L-Fucitol
pma2400	167.10	94.99	166.14	C11H18O	[M+H]+	Organic acids	Dihydrojasmone
NK10253223	168.06	94.06	167.06	C8H9NO3	[M+H]+	Phenolic acids	2-Amino-3-methoxybenzoic acid
Zmjp000624	168.06	150.05	167.06	C8H9NO3	[M+H]+	Others	Pyridoxal
Zmsp001834	169.05	65.00	168.04	C8H8O4	[M+H]+	Quinones	2,6-Dimethoxy-1,4-benzoquinone
MWSmce501	167.04	108.02	168.04	C8H8O4	[M-H]-	Phenolic acids	Protocatechuic Acid Methyl Ester
pme1292	167.10	123.04	168.04	C8H8O4	[M-H]-	Phenolic acids	Homogentisic acid
pme1383	170.08	134.10	169.07	C8H11NO3	[M+H]+	Others	Pyridoxine
ML10176345	171.03	109.03	172.04	C7H8O5	[M-H]-	Organic acids	3-Dehydroshikimic acid
pme0253	174.11	86.00	173.11	C8H15NO3	[M+H]+	Amino acids and derivatives	N-Acetyl-L-leucine
pme3009	175.02	99.00	174.02	C6H6O6	[M+H]+	Organic acids	Trans-Citridic acid
MA10039492	173.01	71.03	174.02	C6H6O6	[M-H]-	Others	Dehydroascorbic acid
mws0154	173.05	93.00	174.05	C7H10O5	[M-H]-	Organic acids	Shikimic acid
mws0242	173.08	111.00	174.09	C8H14O4	[M-H]-	Organic acids	Suberic Acid
Zmyn000155	173.11	131.08	174.10	C7H14N2O3	[M-H]-	Amino acids and derivatives	N-α-Acetyl-L-ornithine
pme2559	174.04	88.00	175.05	C6H9NO5	[M-H]-	Amino acids and derivatives	N-Acetyl-L-Aspartic Acid
pmb0813	176.10	133.20	175.06	C10H9NO2	[M+H]+	Alkaloids	1-Methoxyindole-3-carbaldehyde
mws4175	175.02	85.03	176.03	C6H8O6	[M-H]-	Others	D-Glucurono-6,3-lactone
mws1075	177.05	121.00	176.05	C10H8O3	[M+H]+	Lignans and Coumarins	7-Methoxycoumarin
Zmgn001448	175.06	115.04	176.07	C7H12O5	[M-H]-	Organic acids	2-Propylmalic Acid

mws1500	177.09	130.90	176.08	C11H12O2	[M+H]+	Phenolic acids	Ethyl cinnamate
mws1013	177.02	133.20	178.03	C9H6O4	[M-H]-	Lignans and Coumarins	Esculetin
MWSmce220	177.04	59.02	178.05	C6H10O6	[M-H]-	Organic acids	D-Glucono-1,5-lactone
pmb2795	177.00	145.00	178.06	C10H10O3	[M-H]-	Phenolic acids	4-Methoxycinnamic acid
mws0981	178.04	136.02	179.04	C6H5N5O2	[M-H]-	Nucleotides and derivatives	Isoxanthopterin
pmb0786	180.09	163.06	179.08	C6H13NO5	[M+H]+	Others	D-Glucosamine
mws2212	179.03	135.05	180.04	C9H8O4	[M-H]-	Phenolic acids	Caffeic acid
mws1164	179.06	59.01	180.06	C6H12O6	[M-H]-	Others	D-Fructose*
mws4170	179.06	59.01	180.06	C6H12O6	[M-H]-	Others	D-Glucose*
Hmln000297	179.05	59.01	180.06	C6H12O6	[M-H]-	Others	Inositol*
mws0093	179.07	146.00	180.08	C10H12O3	[M-H]-	Phenolic acids	Coniferyl alcohol
MWS2070	179.07	92.03	180.08	C10H12O3	[M-H]-	Phenolic acids	Propyl 4-hydroxybenzoate
mws0250	182.08	136.10	181.07	C9H11NO3	[M+H]+	Amino acids and derivatives	L-Tyrosine*
MWStz070	182.08	91.05	181.07	C9H11NO3	[M+H]+	Alkaloids	N-(2-Hydroxy-4-methoxyphenyl)acetamide
Zmsp000878	182.08	136.08	181.07	C9H11NO3	[M+H]+	Alkaloids	4-Hydroxy-5-(2-oxo-1-pyrrolidinyl)benzoic acid*
MWS5206	181.05	135.04	182.06	C9H10O4	[M-H]-	Phenolic acids	Hydroxyphenyllactic acid*
Lmrn001951	181.05	135.04	182.06	C9H10O4	[M-H]-	Phenolic acids	(S)-2-Hydroxy-3-(4-Hydroxyphenyl)Propanoic Acid*
Hmtn001288	181.05	135.04	182.06	C9H10O4	[M-H]-	Phenolic acids	Methyl 2,4-dihydroxyphenylacetate*
mws1155	181.07	101.02	182.08	C6H14O6	[M-H]-	Others	Mannitol*
pme2237	181.07	101.00	182.08	C6H14O6	[M-H]-	Others	Dulcitol*
pme2596	184.06	148.00	183.05	C8H9NO4	[M+H]+	Others	4-Pyridoxic acid
pmb1754	184.00	125.00	184.07	C5H15NO4P+	[M]+	Alkaloids	O-Phosphocholine
MWS1816	183.14	183.14	184.15	C11H20O2	[M-H]-	Lipids	10-Undecenoic acid
mws0752	185.15	185.16	186.16	C11H22O2	[M-H]-	Lipids	Undecylic Acid
Hmmp001310	188.07	118.07	187.06	C11H9NO2	[M+H]+	Alkaloids	3-Indoleacrylic acid
pme0137	187.07	125.00	188.08	C7H12N2O4	[M-H]-	Amino acids and derivatives	N-Acetyl-L-Glutamine

mws0237	187.10	125.00	188.11	C9H16O4	[M-H]-	Organic acids	Azelaic acid
pme0122	189.12	126.00	188.12	C8H16N2O3	[M+H]+	Amino acids and derivatives	N6-Acetyl-L-lysine
mws5041	189.12	86.10	188.12	C8H16N2O3	[M+H]+	Amino acids and derivatives	L-Glycyl-L-isoleucine
Zmdp000292	189.14	70.07	188.13	C7H16N4O2	[M+H]+	Amino acids and derivatives	Arginine methyl ester*
Zmjp000182	189.13	70.07	188.13	C7H16N4O2	[M+H]+	Amino acids and derivatives	N-Monomethyl-L-arginine*
pme0075	188.06	128.00	189.06	C7H11NO5	[M-H]-	Amino acids and derivatives	N-Acetyl-L-glutamic acid
pme2244	190.09	118.07	189.07	C11H11NO2	[M+H]+	Alkaloids	3-Indolepropionic acid
mws0597	190.05	146.00	191.06	C10H9NO3	[M-H]-	Alkaloids	5-Hydroxyindole-3-acetic acid
Zmyn000453	191.02	111.01	192.03	C6H8O7	[M-H]-	Organic acids	Isocitric Acid
Hmjp002337	193.04	133.03	192.03	C10H8O4	[M+H]+	Lignans and Coumarins	Isoscopoletin (6-Hydroxy-7-Methoxycoumarin)
Cmpp003619	193.05	105.07	192.04	C10H8O4	[M+H]+	Lignans and Coumarins	6,7-Dihydroxy-4-methylcoumarin
pme2993	193.05	133.00	192.04	C10H8O4	[M+H]+	Lignans and Coumarins	Scopoletin (7-Hydroxy-6-methoxycoumarin)
mws0277	191.06	85.00	192.06	C7H12O6	[M-H]-	Organic acids	Quinic Acid
MWSslk144	193.09	147.04	192.08	C11H12O3	[M+H]+	Phenolic acids	p-Coumaric acid ethyl ester
Lmmp002080	193.13	105.03	192.13	C11H16N2O	[M+H]+	Alkaloids	N-(4-Aminobutyl)benzamide
pme2743	194.08	91.00	193.07	C10H11NO3	[M+H]+	Amino acids and derivatives	N-Phenylacetylglycine
pme3705	193.04	73.00	194.04	C6H10O7	[M-H]-	Others	D-Glucoronic acid
Lmdn003756	193.05	135.05	194.06	C10H10O4	[M-H]-	Phenolic acids	Methyl caffeate
MWSslk097	195.06	163.04	194.06	C10H10O4	[M+H]+	Phenolic acids	Dimethyl phthalate
XLn05261	195.03	108.02	196.04	C9H8O5	[M-H]-	Phenolic acids	Salicylacetic acid
pme0534	195.05	74.90	196.06	C6H12O7	[M-H]-	Others	Gluconic acid
Zmzn000079	199.00	78.96	200.01	C4H9O7P	[M-H]-	Others	D-Erythrose-4-phosphate
pmb2640	199.17	199.17	200.18	C12H24O2	[M-H]-	Lipids	Dodecanoic acid (Lauric acid)
MWS5165	203.09	129.05	202.08	C9H14O5	[M+H]+	Others	Methyloxalacetic Acid Diethyl Ester
pmb0130	203.12	144.00	202.11	C12H14N2O	[M+H]+	Alkaloids	Acetryptine
MWS5164	203.15	70.07	202.14	C8H18N4O2	[M+H]+	Amino acids and derivatives	N,N'-Dimethylarginine;SDMA

mws0018	203.22	83.00	202.22	C10H26N4	[M+H]+	Alkaloids	Spermine
Zmbp002538	205.10	146.06	204.09	C11H12N2O2	[M+H]+	Alkaloids	1-Methoxy-indole-3-acetamide
mws0282	203.08	116.05	204.09	C11H12N2O2	[M-H]-	Amino acids and derivatives	L-Tryptophan
pmb0818	206.10	147.50	205.07	C11H11NO3	[M+H]+	Alkaloids	Methoxyindoleacetic acid
MWS0618	204.12	71.05	205.13	C9H19NO4	[M-H]-	Others	Pantothenol
MWS1877	205.16	133.06	206.17	C14H22O	[M-H]-	Phenolic acids	4-tert-Octylphenol
Lmbn013410	205.16	189.13	206.17	C14H22O	[M-H]-	Phenolic acids	2,4-Di-Tert-Butylphenol
Zmgn002106	206.08	58.03	207.09	C11H13NO3	[M-H]-	Amino acids and derivatives	N-Acetyl-L-phenylalanine
mws1014	209.04	149.10	208.04	C10H8O5	[M+H]+	Lignans and Coumarins	Fraxetin (7,8-Dihydroxy-6-methoxycoumarin)
MA10107783	207.03	119.05	208.04	C10H8O5	[M-H]-	Phenolic acids	3-[(1-Carboxyvinyl)oxy]benzoic acid
pme3443	207.07	192.00	208.07	C11H12O4	[M-H]-	Phenolic acids	Sinapinaldehyde
mws2184	207.07	135.10	208.07	C11H12O4	[M-H]-	Phenolic acids	Ethyl caffeate
pmb2620	207.07	133.10	208.07	C11H12O4	[M-H]-	Phenolic acids	3,4-Dimethoxycinnamic acid
mws1212	209.08	177.30	208.07	C11H12O4	[M+H]+	Phenolic acids	Methyl ferulate
Zmyn000108	209.03	85.03	210.04	C6H10O8	[M-H]-	Others	D-Saccharic acid*
Zmpn000199	209.03	85.03	210.04	C6H10O8	[M-H]-	Others	D-Galactaric acid*
Hmcn000192	209.07	59.01	210.07	C7H14O7	[M-H]-	Others	Sedoheptulose
mws0853	209.08	179.00	210.09	C11H14O4	[M-H]-	Phenolic acids	Sinapyl alcohol
pme1654	209.12	59.02	210.13	C12H18O3	[M-H]-	Organic acids	Jasmonic acid
Zmgn003633	211.13	183.14	212.14	C12H20O3	[M-H]-	Lipids	12-Oxo-10E-Dodecenoic Acid
mws1509	215.07	121.00	214.06	C13H10O3	[M+H]+	Phenolic acids	Phenyl salicylate
mws0751	213.19	195.00	214.19	C13H26O2	[M-H]-	Lipids	Tridecanoic Acid
pme0170	217.13	158.00	216.12	C8H16N4O3	[M+H]+	Amino acids and derivatives	N-Acetyl-L-Arginine
MWS1900	215.13	153.13	216.14	C11H20O4	[M-H]-	Lipids	Undecanedioic acid
mws5045	215.17	169.16	216.17	C12H24O3	[M-H]-	Lipids	12-Hydroxydodecanoic acid
pme2566	217.08	199.00	218.09	C8H14N2O5	[M-H]-	Amino acids and derivatives	5-L-Glutamyl-L-amino acid

mws0677	219.11	160.00	218.11	C12H14N2O2	[M+H]+	Alkaloids	N-Acetyl-5-hydroxytryptamine
pmb0426	219.10	174.40	218.14	C13H18N2O	[M+H]+	Alkaloids	N,N-Dimethyl-5-methoxytryptamine
MWSmce257	218.11	88.04	219.11	C9H17NO5	[M-H]-	Others	D-Pantothenic Acid
pme1228	221.09	204.00	220.09	C11H12N2O3	[M+H]+	Amino acids and derivatives	5-Hydroxy-L-tryptophan
Hmyp009454	221.19	119.08	220.18	C15H24O	[M+H]+	Terpenoids	Nootkatol
Zjyp102918	221.19	119.08	220.18	C15H24O	[M+H]+	Terpenoids	epinootkatol
Lmtp005226	221.19	105.07	220.18	C15H24O	[M+H]+	Others	Santalol
pme2755	222.00	144.00	221.09	C8H15NO6	[M+H]+	Others	N-Acetyl-D-glucosamine
mws2608	222.10	84.05	221.09	C8H15NO6	[M+H]+	Others	N-Acetyl-D-galactosamine
MWS20178	223.06	190.03	222.05	C11H10O5	[M+H]+	Lignans and Coumarins	isofraxidin
mws5042	223.11	120.09	222.10	C11H14N2O3	[M+H]+	Amino acids and derivatives	L-Glycyl-L-phenylalanine
Hmcp003852	223.21	207.03	222.20	C15H26O	[M+H]+	Terpenoids	Elemol
mws0520	224.09	136.00	223.08	C11H13NO4	[M+H]+	Amino acids and derivatives	N-Acetyl-L-tyrosine
HJAP044	225.11	167.06	224.11	C12H16O4	[M+H]+	Phenolic acids	1-(2,4,5-Trimethoxyphenyl)propan-1-one
pme0220	225.15	151.11	224.14	C13H20O3	[M+H]+	Organic acids	Methyl jasmonate
Cmsp002787	227.09	181.05	226.08	C11H14O5	[M+H]+	Phenolic acids	3,4'-Dihydroxy-3',5'-dimethoxypropiophenone
HJAP051	227.13	181.09	226.12	C12H18O4	[M+H]+	Phenolic acids	3-(3,4,5-Trimethoxyphenyl)propan-1-ol
pme1194	228.10	112.00	227.09	C9H13N3O4	[M+H]+	Nucleotides and derivatives	2'-Deoxycytidine
mws0119	227.20	227.20	228.21	C14H28O2	[M-H]-	Lipids	Myristic Acid
pma6455	229.01	96.97	230.02	C5H11O8P	[M-H]-	Others	Ribulose-5-phosphate
Zmwp007375	231.14	185.13	230.13	C15H18O2	[M+H]+	Terpenoids	Dehydrocostuslactone
mws0474	229.14	211.00	230.15	C12H22O4	[M-H]-	Organic acids	Dodecanedioic aicd
Lmhp001670	231.16	72.08	230.16	C11H22N2O3	[M+H]+	Amino acids and derivatives	L-Valyl-L-Leucine
Lmjp007763	233.15	187.15	232.15	C15H20O2	[M+H]+	Terpenoids	6,7-Dehydroartemisinic acid
Lhhp102907	233.15	131.08	232.15	C15H20O2	[M+H]+	Terpenoids	Costunolide
pmb0962	235.10	118.30	234.16	C10H22N2O4	[M+H]+	Amino acids and derivatives	L-Lysine-Butanoic Acid

Jmcp010061	235.17	189.16	234.16	C15H22O2	[M+H]+	Terpenoids	costic acid*
MWSmce502	235.17	189.16	234.16	C15H22O2	[M+H]+	Terpenoids	Artemisinic acid*
Jmbn003202	235.12	59.01	236.13	C10H20O6	[M-H]-	Others	Butyl Beta-D-Fructopyranoside
Lmcp006516	237.18	201.16	236.18	C15H24O2	[M+H]+	Terpenoids	Baimuxinal
Lmyn000160	237.06	87.01	238.07	C8H14O8	[M-H]-	Phenolic acids	Mucic acid Dimethyl Ester
mws0221	241.03	152.00	240.02	C6H12N2O4S2	[M+H]+	Amino acids and derivatives	L-Cystine
MWS0552	239.20	223.03	240.21	C15H28O2	[M-H]-	Lipids	Cis-10-Pentadecenoic Acid(C15: 1)
pme0264	243.10	127.00	242.09	C10H14N2O5	[M+H]+	Nucleotides and derivatives	Thymidine
MWS2430	241.22	241.21	242.23	C15H30O2	[M-H]-	Lipids	13-methylmyristic acid
pme3732	244.09	112.00	243.09	C9H13N3O5	[M+H]+	Nucleotides and derivatives	Cytidine
ML10180524	244.09	112.05	243.09	C9H13N3O5	[M+H]+	Nucleotides and derivatives	Cytarabine
mws0248	243.06	110.00	244.07	C9H12N2O6	[M-H]-	Nucleotides and derivatives	Uridine
pme2266	245.10	227.00	244.09	C10H16N2O3S	[M+H]+	Others	Biotin
Lmhp002031	245.18	86.10	244.18	C12H24N2O3	[M+H]+	Amino acids and derivatives	L-Leucyl-L-Leucine
Zmdp001647	247.13	184.10	246.12	C10H18N2O5	[M+H]+	Amino acids and derivatives	γ-Glutamyl-L-valine
MWSmce062	247.13	131.08	246.13	C15H18O3	[M+H]+	Terpenoids	Xanthatin
Hmcp003285	249.15	185.13	248.14	C15H20O3	[M+H]+	Terpenoids	Santamarin
Hmcn003273	247.13	203.14	248.14	C15H20O3	[M-H]-	Terpenoids	Reynosin
Qmqp102523	249.15	175.11	248.14	C15H20O3	[M+H]+	Terpenoids	Pechueloic acid
Lhhp102906	249.15	145.10	248.14	C15H20O3	[M+H]+	Terpenoids	Parthenolide
pme2563	249.06	128.00	250.06	C8H14N2O5S	[M-H]-	Amino acids and derivatives	γ-Glu-Cys
Jmcn007352	249.15	203.14	250.16	C15H22O3	[M-H]-	Terpenoids	5α-Hydroxycostic acid
pme3961	252.11	136.00	251.10	C10H13N5O3	[M+H]+	Nucleotides and derivatives	2'-Deoxyadenosine
Lmcn009122	253.22	235.21	254.23	C16H30O2	[M-H]-	Lipids	(7Z)-Hexadecenoic acid
mws0361	253.22	235.19	254.23	C16H30O2	[M-H]-	Lipids	Palmitoleic Acid
MWSHY0124	257.08	153.02	256.07	C15H12O4	[M+H]+	Flavonoids	Pinocembrin (Dihydrochrysin)

pma3101	256.20	124.10	256.08	C11H14NO6+	[M+H]+	Others	Nicotinate D-ribonucleoside
mws1488	255.23	237.22	256.24	C16H32O2	[M-H]-	Lipids	Palmitic acid
mws0120	258.11	104.11	257.10	C8H20NO6P	[M+H]+	Lipids	Choline Alfoscerate
MWSslk133	257.18	195.18	258.18	C14H26O4	[M-H]-	Lipids	1,14-Tetradecanedioic Acid
Zmyn000110	258.04	78.96	259.05	C6H14NO8P	[M-H]-	Others	D-Glucosamine 1-phosphate
mws0866	259.02	97.00	260.03	C6H13O9P	[M-H]-	Others	D-Glucose 6-phosphate*
MWS2442	259.02	96.97	260.03	C6H13O9P	[M-H]-	Others	D-Fructose 6-Phosphate*
Zmdp002216	261.14	86.10	260.14	C11H20N2O5	[M+H]+	Amino acids and derivatives	L-γ-Glutamyl-L-leucine
MWS4309	262.12	188.07	261.11	C13H15N3O3	[M+H]+	Amino acids and derivatives	Glycyl-tryptophan
Zmpn000095	261.04	78.96	262.05	C6H15O9P	[M-H]-	Others	Sorbitol-6-phosphate
Lmhp001732	263.13	70.07	262.13	C14H18N2O3	[M+H]+	Amino acids and derivatives	L-Prolyl-L-Phenylalanine
mws0715	263.10	145.00	264.11	C13H16N2O4	[M-H]-	Amino acids and derivatives	Phenylacetyl-L-glutamine
Lmtn004049	263.13	204.12	264.14	C15H20O4	[M-H]-	Organic acids	Abscisic acid
Lmhp002001	265.15	72.08	264.15	C14H20N2O3	[M+H]+	Amino acids and derivatives	L-Valyl-L-Phenylalanine
Zmgn001039	266.09	134.05	267.08	C9H17NO6S	[M-H]-	Amino acids and derivatives	S-Ribosyl-L-homocysteine
Zmjp000966	268.11	136.06	267.10	C10H13N5O4	[M+H]+	Others	Vidarabine*
pme0230	268.10	136.06	267.10	C10H13N5O4	[M+H]+	Nucleotides and derivatives	Adenosine*
pma2172	268.13	121.00	267.13	C17H17NO2	[M+H]+	Alkaloids	Cinnamoyltyramine
mws0037	267.07	252.00	268.07	C16H12O4	[M-H]-	Flavonoids	Formononetin (7-Hydroxy-4'-methoxyisoflavone)
mws1060	267.07	135.00	268.08	C10H12N4O5	[M-H]-	Nucleotides and derivatives	9-(Arabinosyl)hypoxanthine
MWS20151	271.06	153.02	270.05	C15H10O5	[M+H]+	Flavonoids	Apigenin; 4',5,7-Trihydroxyflavone
MWS20169	269.05	254.02	270.05	C15H10O5	[M-H]-	Quinones	2,5-dihydroxy-1-methoxy-anthraquinone
Lmyn006227	269.05	151.00	270.05	C15H10O5	[M-H]-	Flavonoids	3,5,7-Trihydroxyflavone
mws1297	269.10	101.02	270.11	C13H18O6	[M-H]-	Phenolic acids	Benzyl glucoside
MWSmce675	271.08	108.02	272.09	C12H16O7	[M-H]-	Phenolic acids	Arbutin*
Ymjm000152	271.08	108.02	272.09	C12H16O7	[M-H]-	Phenolic acids	p-Hydroxypheny-β-D-allopyranoside*

Lmmn003323	271.23	225.22	272.24	C16H32O3	[M-H]-	Organic acids	2-Hydroxyhexadecanoic acid
pmp001264	274.27	256.27	273.27	C16H35NO2	[M+H]+	Lipids	Hexadecylsphingosine
pme1201	273.08	167.00	274.08	C15H14O5	[M-H]-	Flavonoids	Phloretin
pmb0034	277.10	114.70	276.10	C10H16N2O7	[M+H]+	Amino acids and derivatives	L-α-Glutamyl-L-Glutamic Acid
pmb1574	277.20	93.00	276.21	C18H28O2	[M+H]+	Lipids	9,12-Octadecadien-6-Ynoic Acid
mws1038	277.12	146.00	278.13	C11H22N2O4S	[M-H]-	Others	Pantetheine
Lmlp012720	279.16	149.02	278.15	C16H22O4	[M+H]+	Phenolic acids	Dibutyl phthalate*
Lmxp011770	279.16	149.02	278.15	C16H22O4	[M+H]+	Phenolic acids	Diisobutyl phthalate*
mws5035	279.17	120.08	278.16	C15H22N2O3	[M+H]+	Amino acids and derivatives	L-Leucyl-L-phenylalanine
mws0367	277.22	277.22	278.23	C18H30O2	[M-H]-	Lipids	α-Linolenic Acid
MWSmce692	277.22	59.01	278.23	C18H30O2	[M-H]-	Lipids	γ-Linolenic Acid
pmb1650	279.20	149.40	278.23	C18H30O2	[M+H]+	Lipids	Octadeca-11E,13E,15Z-trienoic acid
Lmbn005923	277.22	59.01	278.23	C18H30O2	[M-H]-	Lipids	Crepenynic acid
Wmln002759	278.11	131.04	279.29	C14H17NO5	[M-H]-	Alkaloids	4-(Rhamnosyloxy)phenylacetonitrile
Lmhn002926	279.05	163.04	280.06	C13H12O7	[M-H]-	Phenolic acids	p-Coumaroylmalic acid
mws0629	281.11	166.00	280.11	C13H16N2O5	[M+H]+	Amino acids and derivatives	L-Aspartyl-L-Phenylalanine
mws1491	279.23	279.23	280.24	C18H32O2	[M-H]-	Lipids	Linoleic acid
Lmbn006152	279.23	59.01	280.24	C18H32O2	[M-H]-	Lipids	(9Z,11E)-Octadecadienoic acid
Hmhp001812	282.12	136.06	281.11	C11H15N5O4	[M+H]+	Nucleotides and derivatives	2'-O-Methyladenosine
YC512118	282.28	247.24	281.27	C18H35NO	[M+H]+	Lipids	Oleamide (9-Octadecenamide)
Lmyn012331	281.25	281.25	282.26	C18H34O2	[M-H]-	Lipids	Petroselinic acid
mws2623	281.25	281.25	282.26	C18H34O2	[M-H]-	Lipids	11-Octadecanoic acid(Vaccenic acid)
pme1178	284.10	152.00	283.09	C10H13N5O5	[M+H]+	Nucleotides and derivatives	Guanosine
MWSmce089	284.12	147.04	283.12	C17H17NO3	[M+H]+	Alkaloids	p-Coumaroyltyramine
Zmzp005934	284.30	102.09	283.29	C18H37NO	[M+H]+	Alkaloids	Stearamide
mws0129	285.08	270.00	284.07	C16H12O5	[M+H]+	Flavonoids	Apigenin 7-methyl ether*

mws0051	285.08	270.00	284.07	C16H12O5	[M+H]+	Flavonoids	Acacetin*
mws0918	283.06	268.00	284.07	C16H12O5	[M-H]-	Flavonoids	Prunetin (5,4'-Dihydroxy-7-methoxyisoflavone)
mws4160	285.08	270.07	284.07	C16H12O5	[M+H]+	Flavonoids	5,7-Dihydroxy-8-Methoxyflavone*
mws0668	283.07	151.00	284.08	C10H12N4O6	[M-H]-	Nucleotides and derivatives	Xanthosine
mws1505	283.10	135.00	284.11	C17H16O4	[M-H]-	Phenolic acids	Phenethyl caffeate
Zmyn005026	283.26	283.26	284.27	C18H36O2	[M-H]-	Lipids	16-Methylheptadecanoic acid
mws1489	283.26	283.26	284.27	C18H36O2	[M-H]-	Lipids	Stearic Acid
Hmbp002730	287.06	153.01	286.05	C15H10O6	[M+H]+	Flavonoids	Isoscutellarein*
Lmmp004504	287.06	153.02	286.05	C15H10O6	[M+H]+	Flavonoids	2'-Hydroxygenistein*
MWSHY0058	287.06	153.02	286.05	C15H10O6	[M+H]+	Flavonoids	Luteolin (5,7,3',4'-Tetrahydroxyflavone)*
MWSslk132	285.21	223.21	286.21	C16H30O4	[M-H]-	Lipids	Hexadecanedioic acid
pme2289	287.20	119.00	286.23	C20H30O	[M+H]+	Others	Vitamin A (Retinol)
pmp000727	288.13	177.05	287.13	C15H17N3O3	[M+H]+	Alkaloids	Feruloylhistamine
MWSHY0145	289.07	153.02	288.06	C15H12O6	[M+H]+	Flavonoids	Eriodictyol (5,7,3',4'-Tetrahydroxyflavanone)*
Cmxp005429	289.07	153.02	288.06	C15H12O6	[M+H]+	Flavonoids	Okanin*
Lmsp004137	289.07	163.04	288.06	C15H12O6	[M+H]+	Flavonoids	3,4,2',4',6'-Pentahydroxychalcone
pmn001686	287.22	241.22	288.23	C16H32O4	[M-H]-	Lipids	10,16-Dihydroxypalmitic acid
mws1496	290.18	124.00	289.17	C17H23NO3	[M+H]+	Alkaloids	L-Hyoscyamine
pmb3081	289.00	97.00	290.00	C6H11PO11	[M-H]-	Others	Glucarate O-Phosphoric acid
pme3163	289.00	97.00	290.04	C7H15O10P	[M-H]-	Others	D-Sedoheptuiose 7-phosphate
pmb0885	291.00	190.90	290.19	C18H26O3	[M+H]+	Lipids	4-Oxo-9Z,11Z,13E,15E-Octadecatetraenoic Acid
Zmyn004548	291.20	235.17	292.20	C18H28O3	[M-H]-	Lipids	12-Oxo-phytodienoic acid
MWSmce689	293.25	95.09	292.24	C19H32O2	[M+H]+	Lipids	Methyl linolenate
Zmdn001564	293.11	164.07	294.12	C14H18N2O5	[M-H]-	Amino acids and derivatives	γ-Glutamylphenylalanine
Zmyn004676	293.21	113.10	294.22	C18H30O3	[M-H]-	Lipids	17-Hydroxylinolenic acid
Zmyn004732	293.21	185.12	294.22	C18H30O3	[M-H]-	Lipids	2R-hydroxy-9Z,12Z,15Z-octadecatrienoic acid

pmb2787	293.21	275.10	294.22	C18H30O3	[M-H]-	Lipids	9-Oxo-10E,12Z-octadecadienoic acid
Lmbn005443	293.21	235.17	294.22	C18H30O3	[M-H]-	Lipids	13-KODE; (9Z,11E)-13-Oxooctadeca-9,11-dienoic acid
Lmqp000329	296.07	104.11	295.06	C8H14N3O7P	[M+H]+	Nucleotides and derivatives	5-Aminoimidazole ribonucleotide
Lmhn102068	295.05	179.03	296.05	C13H12O8	[M-H]-	Phenolic acids	2-O-Caffeoylmalic acid
Hmqp005411	297.24	81.07	296.23	C18H32O3	[M+H]+	Lipids	9-Oxo-12Z-Octadecenoic acid
Hmqp005455	297.24	81.07	296.23	C18H32O3	[M+H]+	Lipids	15(R)-Hydroxylinoleic Acid
Lmbn005662	295.23	171.10	296.24	C18H32O3	[M-H]-	Lipids	9(10)-EpOME;(9R,10S)-(12Z)-9,10-Epoxyoctadecenoic acid
Rfmb091	295.23	195.14	296.24	C18H32O3	[M-H]-	Lipids	9S-Hydroxy-10E,12Z-octadecadienoic acid
pmb2799	295.23	195.20	296.24	C18H32O3	[M-H]-	Lipids	12,13-Epoxy-9-Octadecenoic Acid
pme1474	298.10	136.00	297.09	C11H15N5O3S	[M+H]+	Nucleotides and derivatives	5'-Deoxy-5'-(methylthio)adenosine
pmb0197	298.10	166.30	297.11	C11H15N5O5	[M+H]+	Nucleotides and derivatives	N7-Methylguanosine
Zmyn004714	297.24	183.14	298.25	C18H34O3	[M-H]-	Lipids	Ricinoleic acid
pmf0297	297.32	183.10	298.32	C20H42O	[M-H]-	Lipids	1-Eicosanol
mws0058	299.06	284.00	300.06	C16H12O6	[M-H]-	Flavonoids	Diosmetin (5,7,3'-Trihydroxy-4'-methoxyflavone)*
MWSHY0069	301.07	286.05	300.06	C16H12O6	[M+H]+	Flavonoids	Hispidulin (5,7,4'-Trihydroxy-6-methoxyflavone)*
MWS20189	301.07	286.05	300.06	C16H12O6	[M+H]+	Flavonoids	Tectorigenin*
Zmhn003257	299.06	284.04	300.06	C16H12O6	[M-H]-	Flavonoids	5,7,2'-Trhiyroxy-8-methoxyflavone*
Zmhn001926	299.08	137.02	300.08	C13H16O8	[M-H]-	Phenolic acids	1-O-Salicyloyl-β-D-glucose*
Zmhn001358	299.08	137.02	300.08	C13H16O8	[M-H]-	Phenolic acids	4-O-Glucosyl-4-hydroxybenzoic acid*
pmc1990	299.10	223.10	300.10	C17H16O5	[M-H]-	Flavonoids	4'-Hydroxy-5,7-dimethoxyflavanone
mws2367	299.11	119.00	300.12	C14H20O7	[M-H]-	Phenolic acids	Salidroside
MWS5235	299.26	253.25	300.27	C18H36O3	[M-H]-	Lipids	12-Hydroxyoctadecanoic acid
Zmyn005384	299.26	253.26	300.27	C18H36O3	[M-H]-	Lipids	2R-Hydroxyoctadecanoic Acid
pmp001176	302.14	103.10	301.13	C17H19NO4	[M+H]+	Alkaloids	Dihydrocaffeoyltyramine
MWSmce521	302.17	137.06	301.17	C18H23NO3	[M+H]+	Alkaloids	Dobutamine

mws0920	301.04	151.00	302.04	C15H10O7	[M-H]-	Flavonoids	Tricetin (5,7,3',4',5'-Pentahydroxyflavone)
Zmhn000892	301.09	139.05	302.10	C13H18O8	[M-H]-	Phenolic acids	4-O-Glucosyl-3,4-dihydroxybenzyl alcohol
MWSmce549	303.25	57.07	302.25	C17H34O4	[M+H]+	Lipids	1-Monomyristin
mws1375	304.15	185.09	303.14	C12H21N3O6	[M+H]+	Alkaloids	Nicotianamine
mws0042	305.07	125.00	306.07	C15H14O7	[M-H]-	Flavonoids	Epigallocatechin
pme1373	306.05	110.00	307.06	C9H14N3O7P	[M-H]-	Nucleotides and derivatives	2'-Deoxycytidine-5'-monophosphate
pme1086	306.08	143.00	307.08	C10H17N3O6S	[M-H]-	Amino acids and derivatives	Glutathione reduced form
pmb2601	307.10	161.10	308.09	C15H16O7	[M-H]-	Lignans and Coumarins	7-Hydroxycoumarin-O-rhamnoside
pmn001610	307.26	307.30	308.27	C20H36O2	[M-H]-	Lipids	Eicosadienoic acid
pmb2857	308.10	146.10	309.11	C11H19NO9	[M-H]-	Amino acids and derivatives	L-Glutamic acid-O-glycoside
Lmhn003074	309.06	193.05	310.07	C14H14O8	[M-H]-	Phenolic acids	Feruloylmalic acid
pmb2791	309.21	209.10	310.10	C18H30O4	[M-H]-	Lipids	9-Hydroperoxy-10E,12,15Z-octadecatrienoic acid
Zmzn003953	309.20	227.20	310.21	C18H30O4	[M-H]-	Lipids	13(s)-hydroperoxy-(9z,11e,15z)-octadecatrienoic acid
Hmqp006221	311.26	279.32	310.25	C19H34O3	[M+H]+	Lipids	13-Hydroxy-9Z,11E-octadecadienoic acid
Lmtp001399	312.11	150.05	311.10	C14H17NO7	[M+H]+	Alkaloids	5,6-Dihydroxyindole-5-O-β-glucoside
pme3967	312.13	180.00	311.12	C12H17N5O5	[M+H]+	Nucleotides and derivatives	2-(Dimethylamino)guanosine
pmb2804	311.10	171.30	312.23	C18H32O4	[M-H]-	Lipids	13S-Hydroperoxy-9Z,11E-octadecadienoic acid
pmn001689	311.22	223.17	312.23	C18H32O4	[M-H]-	Lipids	9-Hydroxy-12-oxo-15(Z)-octadecenoic acid
Lmbn004685	311.22	223.17	312.23	C18H32O4	[M-H]-	Lipids	5S,8R-DiHODE; (5S,8R,9Z,12Z)-5,8-Dihydroxyoctadeca-9,12-dienoate
HJAP057	315.09	282.06	314.08	C17H14O6	[M+H]+	Flavonoids	Dihydroxy-dimethoxyflavone*
MWSmce415	315.09	282.06	314.08	C17H14O6	[M+H]+	Flavonoids	4',5-dihydroxy-6,7-dimethoxyflavone*
Hmxp007521	315.09	300.06	314.08	C17H14O6	[M+H]+	Flavonoids	3',4'-Dihydroxy-7,5'-dimethoxyflavone
Lmyn002403	313.09	101.02	314.10	C14H18O8	[M-H]-	Phenolic acids	Mandelic acid-β-glucoside
Lmmn002179	313.09	107.05	314.10	C14H18O8	[M-H]-	Phenolic acids	Methyl salicylate-2-O-glucoside
Lmbn005487	313.24	251.24	314.25	C18H34O4	[M-H]-	Lipids	12,13-DHOME; (9Z)-12,13-Dihydroxyoctadec-9-enoic acid

Lmbn007891	313.24	183.14	314.25	C18H34O4	[M-H]-	Lipids	Hydroxy ricinoleic acid
pmp000003	317.07	302.04	316.06	C16H12O7	[M+H]+	Flavonoids	5,7,3',4'-Tetrahydroxy-6-methoxyflavone
mws0066	315.05	151.00	316.06	C16H12O7	[M-H]-	Flavonoids	Isorhamnetin
pmn001367	315.07	153.02	316.08	C13H16O9	[M-H]-	Phenolic acids	Protocatechuic acid-4-O-glucoside*
pmb2871	315.07	153.00	316.08	C13H16O9	[M-H]-	Phenolic acids	1-O-Gentisoyl-β-D-glucoside*
Jmwn002172	315.11	135.04	316.12	C14H20O8	[M-H]-	Phenolic acids	3,4-dihydroxyphenylethanol-β-D-glucopyranoside
Zmtn001661	315.11	153.02	316.12	C14H20O8	[M-H]-	Phenolic acids	Vanilloloside
Hmtn001120	315.11	153.06	316.12	C14H20O8	[M-H]-	Phenolic acids	5-(2-Hydroxyethyl)-2-O-glucosylphenol
pmb0751	321.10	147.30	320.09	C16H16O7	[M+H]+	Phenolic acids	Trans-5-O-(p-Coumaroyl)shikimate
mws0582	322.11	130.00	321.10	C11H19N3O6S	[M+H]+	Amino acids and derivatives	S-(Methyl)glutathione
pme2074	322.40	129.70	323.21	C18H29NO4	[M-H]-	Organic acids	(-)-Jasmonoyl-L-Isoleucine
pme3188	323.03	211.00	324.04	C9H13N2O9P	[M-H]-	Nucleotides and derivatives	Uridine 5'-monophosphate
Lmbp003825	325.09	163.04	324.09	C15H16O8	[M+H]+	Lignans and Coumarins	Skimmin (7-Hydroxycoumarin-7-O-glucoside)
Hmqp006023	326.27	309.27	324.27	C20H36O3	[M+H]+	Lipids	Ethyl 9-Hydroxy-10,12-octadecadienoic acid
mws0983	326.31	62.06	325.30	C20H39NO2	[M+H]+	Alkaloids	N-Oleoylethanolamine
pmn001419	325.09	163.04	326.10	C15H18O8	[M-H]-	Phenolic acids	1-O-p-Coumaroyl-β-D-glucose
Jmwn002494	325.09	119.05	326.10	C15H18O8	[M-H]-	Phenolic acids	(2E)-3-[4-(β-D-glucopyranoside)-phenylacrylic]-acid
Hmhn003067	325.09	119.05	326.10	C15H18O8	[M-H]-	Phenolic acids	Phenylpropionic acid-O-β-D-glucopyranoside
HX1255	327.16	203.11	326.15	C20H22O4	[M+H]+	Lignans and Coumarins	(+)-trans-1,2-dihydrodehydroguaiaretic acid
pmn001691	327.22	291.20	328.23	C18H32O5	[M-H]-	Lipids	9,12,13-Trihydroxy-10,15-octadecadienoic acid
mws0884	328.05	134.20	329.05	C10H12N5O6P	[M-H]-	Nucleotides and derivatives	Cyclic 3',5'-Adenylic acid
MWSHY0169	331.08	301.04	330.07	C17H14O7	[M+H]+	Flavonoids	4',5,7-Trihydroxy-3',6-dimethoxyflavone
pmp001168	331.08	168.00	330.07	C17H14O7	[M+H]+	Flavonoids	Iristectorigenin B
Hmgp003130	331.08	316.05	330.08	C17H14O7	[M+H]+	Flavonoids	Iristectorigenin A
Lmtn002565	329.11	167.03	330.10	C14H18O9	[M-H]-	Phenolic acids	1-O-Vanilloyl-D-Glucose*
Lmmn003663	329.09	167.04	330.10	C14H18O9	[M-H]-	Phenolic acids	5-Glucosyloxy-2-Hydroxybenzoic acid methyl ester*

pmn001517	329.12	167.00	330.13	C15H22O8	[M-H]-	Phenolic acids	3,4,5-Trimethoxyphenyl-1-O-Glucoside
Hmqn003054	329.23	311.23	330.24	C18H34O5	[M-H]-	Lipids	9,10,11-Trihydroxy-12-octadecenoic acid
Lmbn003970	329.23	211.14	330.24	C18H34O5	[M-H]-	Lipids	9,12,13-TriHOME; 9(S),12(S),13(S)-Trihydroxy-10(E)-octadecenoic acid
pmp001284	331.28	313.27	330.28	C19H38O4	[M+H]+	Lipids	Monopalmitin
pme3184	332.08	136.00	331.07	C10H14N5O6P	[M+H]+	Nucleotides and derivatives	2'-Deoxyadenosine-5'-monophosphate
pmb0789	332.13	152.07	331.13	C14H21NO8	[M+H]+	Others	Pyridoxine-5'-O-glucoside
pmc0066	333.05	137.05	332.05	C10H13N4O7P	[M+H]+	Nucleotides and derivatives	2'-Deoxyinosine-5'-monophosphate
Lmjn004351	331.05	165.99	332.05	C16H12O8	[M-H]-	Flavonoids	Quercetagetin-4'-Methyl Ether
Lmpn007255	331.04	316.02	332.05	C16H12O8	[M-H]-	Flavonoids	Patuletin (Quercetagetin-6-methyl ether)
Hmln000873	331.07	169.01	332.08	C13H16O10	[M-H]-	Phenolic acids	2-O-Galloyl-D-glucose
Jmwn002117	331.10	153.02	332.11	C14H20O9	[M-H]-	Phenolic acids	2-(3,4-dihydroxyphenyl)ethanediol 1-O-β-D-glucopyranoside*
Lmmn001294	331.10	153.02	332.11	C14H20O9	[M-H]-	Phenolic acids	Koaburaside*
Hmln002806	335.08	179.03	336.09	C16H16O8	[M-H]-	Phenolic acids	5-O-Caffeoylshikimic acid
pmb2636	335.10	253.10	336.23	C20H32O4	[M-H]-	Lipids	8,15-Dihydroxy-5,9,11,13-eicosatetraenoic acid
pmn001421	337.09	191.05	338.10	C16H18O8	[M-H]-	Phenolic acids	3-O-p-Coumaroylquinic acid*
pmb3074	337.09	191.05	338.10	C16H18O8	[M-H]-	Phenolic acids	5-O-p-Coumaroylquinic acid*
pmb4777	337.09	161.10	338.10	C16H18O8	[M-H]-	Lignans and Coumarins	4-Hydroxy-7-methoxycoumarin-β-rhamnoside
Zmyn000083	338.99	78.96	340.00	C6H14O12P2	[M-H]-	Others	D-Glucose 1,6-bisphosphate
Cmyn001328	339.07	177.02	340.08	C15H16O9	[M-H]-	Lignans and Coumarins	Daphnin*
mws1015	339.07	177.00	340.08	C15H16O9	[M-H]-	Lignans and Coumarins	Esculin (6,7-DihydroxyCoumarin-6-glucoside)*
Lmbn001162	339.07	177.02	340.08	C15H16O9	[M-H]-	Lignans and Coumarins	Esculetin-7-O-glucoside*
Hmcn004128	339.11	159.04	340.12	C16H20O8	[M-H]-	Phenolic acids	P-Methoyxcinnamate glucoside
Lmsn002548	341.09	161.02	342.10	C15H18O9	[M-H]-	Phenolic acids	1-O-Caffeoyl-β-D-glucose*
Jmwn002620	341.09	161.02	342.10	C15H18O9	[M-H]-	Phenolic acids	Vanillic Acid-4-O-Glucuronide*

mws0264	341.11	119.00	342.12	C12H22O11	[M-H]-	Others	D-Trehalose
mws0906	341.12	179.00	342.13	C16H22O8	[M-H]-	Phenolic acids	Coniferin
Lmjn005592	343.08	313.04	344.09	C18H16O7	[M-H]-	Flavonoids	4',5-Dihydroxy-3',6,7-trimethoxyflavone
Lmmn000774	343.10	181.05	344.11	C15H20O9	[M-H]-	Phenolic acids	Dihydrocaffeoylglucose
mws0609	344.04	150.00	345.05	C10H12N5O7P	[M-H]-	Nucleotides and derivatives	Guanosine 3',5'-cyclic monophosphate
Hmcp003223	347.07	332.05	346.07	C17H14O8	[M+H]+	Flavonoids	Limocitrin
Hmgp003163	347.08	332.05	346.07	C17H14O8	[M+H]+	Flavonoids	Laciniatin
Lmtn000940	345.08	139.04	346.09	C14H18O10	[M-H]-	Phenolic acids	1-O-(3,4-Dihydroxy-5-methoxy-benzoyl)-glucoside
pmb0981	348.07	136.00	347.06	C10H14N5O7P	[M+H]+	Nucleotides and derivatives	Adenosine 5'-monophosphate
pmb2848	347.10	267.10	348.06	C21H13ClO3	[M-H]-	Lignans and Coumarins	3(2'-Chlorophenyl)-7-hydroxy-4-phenylcoumarin
Lmhp011388	353.27	261.22	352.26	C21H36O4	[M+H]+	Lipids	2-α-Linolenoyl-glycerol
Lmhp011562	353.27	261.22	352.26	C21H36O4	[M+H]+	Lipids	1-α-Linolenoyl-glycerol
mws2108	353.09	191.05	354.10	C16H18O9	[M-H]-	Phenolic acids	Cryptochlorogenic acid (4-O-Caffeoylquinic acid)*
HX1364	355.10	163.04	354.10	C16H18O9	[M+H]+	Lignans and Coumarins	isoscopoletin-glucoside
mws0178	353.09	191.01	354.10	C16H18O9	[M-H]-	Phenolic acids	Chlorogenic acid (3-O-Caffeoylquinic acid)*
pmp001283	355.28	337.27	354.28	C21H38O4	[M+H]+	Lipids	1-Monolinolein
Lmhp112042	355.28	263.24	354.28	C21H38O4	[M+H]+	Lipids	1-Linoleoylglycerol
Lmhp012042	355.28	263.24	354.28	C21H38O4	[M+H]+	Lipids	2-Linoleoylglycerol
Zmhn002422	355.10	193.05	356.11	C16H20O9	[M-H]-	Phenolic acids	1-O-Feruloyl-β-D-glucose
Lssp210123	357.13	137.06	356.13	C20H20O6	[M+H]+	Others	α-Conidendrin
pmb0296	357.30	265.40	356.29	C21H40O4	[M+H]+	Lipids	1-Oleoyl-Sn-Glycerol
Lmsp004450	359.15	131.05	358.14	C20H22O6	[M+H]+	Phenolic acids	Dehydrodiconiferyl alcohol
mws0097	357.13	151.00	358.14	C20H22O6	[M-H]-	Lignans and Coumarins	Pinoresinol
MWS20190	361.09	346.07	360.09	C18H16O8	[M+H]+	Flavonoids	5,7,4'-trihydroxy-6,3',5'-trimethoxyisoflavone
pmb3107	359.10	182.10	360.11	C15H20O10	[M-H]-	Phenolic acids	Glucosyringic Acid
HX1369	361.16	189.09	360.16	C20H24O6	[M+H]+	Lignans and Coumarins	(-)-Lariciresinol

pmb0964	366.20	204.10	365.17	C16H23N5O5	[M+H]+	Nucleotides and derivatives	Isopentenyladenine-7-N-glucoside
Lmgp003270	369.16	177.06	368.07	C16H16O10	[M+H]+	Lignans and Coumarins	Scopoletin-7-O-glucuronide
pma0104	369.12	207.10	368.09	C20H16O7	[M+H]+	Lignans and Coumarins	N-Sinapoylhydroxycoumarin
pmb0752	369.12	177.10	368.11	C17H20O9	[M+H]+	Phenolic acids	3-O-Feruloylquinic acid
mws0179	367.10	190.90	368.11	C17H20O9	[M-H]-	Phenolic acids	Chlorogenic acid methyl ester
pmn001695	371.10	249.06	372.11	C16H20O10	[M-H]-	Phenolic acids	Trihydroxycinnamoylquinic acid
mws0055	373.13	343.09	372.12	C20H20O7	[M+H]+	Flavonoids	4',5,6,7,8-Pentamethoxyflavone
pmb0108	375.20	137.10	374.10	C19H18O8	[M+H]+	Phenolic acids	Feruloyl syringic acid
Lmzn001983	375.13	135.05	376.14	C16H24O10	[M-H]-	Phenolic acids	D-Threo-guaiacylglycerol-7-O-β-D-glucoside
mws0232	377.15	243.00	376.14	C17H20N4O6	[M+H]+	Others	Riboflavin (Vitamin B2)
pme3337	384.12	252.00	383.11	C14H17N5O8	[M+H]+	Nucleotides and derivatives	Succinyladenosine
Lmjp003090	385.11	223.06	384.11	C17H20O10	[M+H]+	Lignans and Coumarins	Isofraxidin-7-O-glucoside
pmb2940	385.11	205.00	386.12	C17H22O10	[M-H]-	Phenolic acids	1-O-Glucosyl sinapate
pmn001423	385.19	223.13	386.19	C19H30O8	[M-H]-	Others	Roseoside
Lmzn001582	387.17	207.10	388.17	C18H28O9	[M-H]-	Phenolic acids	5'-Glucosyloxyjasmanic acid
Lmwp011196	391.28	149.02	390.28	C24H38O4	[M+H]+	Phenolic acids	Bis(2-ethylhexyl)phthalate*
Lmmp010562	391.28	149.02	390.28	C24H38O4	[M+H]+	Phenolic acids	Diisooctyl Phthalate*
mws0043	403.14	373.10	402.13	C21H22O8	[M+H]+	Flavonoids	5,6,7,8,3',4'-Hexamethoxyflavone
Cmjn004337	401.14	269.10	402.15	C18H26O10	[M-H]-	Phenolic acids	Benzyl-(2''-O-xylosyl)glucoside
pme3007	402.99	159.00	404.00	C9H14N2O12P2	[M-H]-	Nucleotides and derivatives	Uridine 5'-diphosphate
pmn001394	405.12	243.10	406.13	C20H22O9	[M-H]-	Others	2,3,5,4'-Tetrahydroxystilbene-2-O-glucoside
pmd0152	409.24	153.00	410.24	C19H39O7P	[M-H]-	Lipids	LysoPA 16:0 (2n isomer)
pmd0153	409.24	153.00	410.24	C19H39O7P	[M-H]-	Lipids	LysoPA 16:0
HJAP012	419.10	383.07	418.09	C20H18O10	[M+H]+	Flavonoids	Luteolin-8-C-arabinoside
MWS20152	417.16	402.10	418.16	C22H26O8	[M-H]-	Lignans and Coumarins	Syringaresinol
mws2523	421.08	241.10	422.08	C12H23O14P	[M-H]-	Others	Trehalose 6-phosphate

Lmhp008337	426.26	285.24	425.25	C19H40NO7P	[M+H]+	Lipids	LysoPE 14:0(2n isomer)
pmb0864	426.26	285.24	425.25	C19H40NO7P	[M+H]+	Lipids	LysoPE 14:0
pme2117	426.00	159.00	427.03	C10H15N5O10P2	[M-H]-	Nucleotides and derivatives	Adenosine 5'-diphosphate
pmp001248	428.19	369.11	428.17	C20H30NO9+	[M]+	Alkaloids	Caffeoylcholine-4-O-glucoside
Cmbn007148	429.12	429.12	430.13	C22H22O9	[M-H]-	Lipids	1-O-Feruloyl-3-O-caffeoylglycerol
pme3504	431.13	269.08	430.13	C22H22O9	[M+H]+	Flavonoids	Formononetin-7-O-glycoside (Ononin)
pmp000413	433.11	283.06	432.11	C21H20O10	[M+H]+	Flavonoids	Genistein-8-C-glucoside
Lmgp004731	433.11	271.06	432.11	C21H20O10	[M+H]+	Flavonoids	Genistein-7-O-galactoside
Lmdp003509	435.09	303.06	434.09	C20H18O11	[M+H]+	Flavonoids	Quercetin-3-O-xyloside*
mws2186	435.09	303.00	434.09	C20H18O11	[M+H]+	Flavonoids	Quercetin-3-O-α-L-arabinofuranoside*
Cmzn005251	433.11	271.06	434.12	C21H22O10	[M-H]-	Phenolic acids	6-O-Caffeoylarbutin
HJN090	433.11	271.06	434.12	C21H22O10	[M-H]-	Flavonoids	Butin-7-O-glucoside
Lmhp008273	438.26	297.24	437.25	C20H40NO7P	[M+H]+	Lipids	LysoPE 15:1(2n isomer)
Lmhp008440	438.26	297.24	437.25	C20H40NO7P	[M+H]+	Lipids	LysoPE 15:1
Lmhp009187	440.28	299.26	439.27	C20H42NO7P	[M+H]+	Lipids	LysoPE 15:0
Lmhp008885	440.28	299.26	439.27	C20H42NO7P	[M+H]+	Lipids	LysoPE 15:0(2n isomer)
Hmcp002316	449.11	317.06	448.10	C21H20O11	[M+H]+	Flavonoids	Isorhamnetin-3-O-arabinoside
Xmyp005654	449.11	287.06	448.10	C21H20O11	[M+H]+	Flavonoids	Kaempferol-4'-O-glucoside*
MWSHY0104	449.11	287.06	448.10	C21H20O11	[M+H]+	Flavonoids	Luteolin-7-O-glucoside*
Lmsn003297	449.11	287.06	450.12	C21H22O11	[M-H]-	Flavonoids	3,4,2',4',6'-Pentahydroxychalcone-4'-O-glucoside
Lmmn004625	449.11	287.06	450.12	C21H22O11	[M-H]-	Flavonoids	Dihydrokaempferol-7-O-glucoside*
MWS20145	451.12	289.07	450.12	C21H22O11	[M+H]+	Flavonoids	Eriodictyol-7-O-glucoside*
Cmxp003975	451.12	289.07	450.12	C21H22O11	[M+H]+	Flavonoids	Okanin-4'-O-glucoside(Marein)*
HJN086	449.11	287.06	450.12	C21H22O11	[M-H]-	Flavonoids	Eriodictyol-3'-O-glucoside*
Jmgn004021	449.11	269.05	450.12	C21H22O11	[M-H]-	Flavonoids	6-C-Glucosyl-2-Hydroxynaringenin
MWSmce674	451.36	89.06	450.35	C31H46O2	[M+H]+	Others	Phylloquinone (Vitamin K1)

Lmhp009034	452.28	311.26	451.27	C21H42NO7P	[M+H]+	Lipids	LysoPE 16:1
Lmhp008763	452.28	311.26	451.27	C21H42NO7P	[M+H]+	Lipids	LysoPE 16:1(2n isomer)
Hmpn005101	451.12	289.07	452.13	C21H24O11	[M-H]-	Flavonoids	Sieboldin
HJN041	451.12	289.07	452.13	C21H24O11	[M-H]-	Flavonoids	Epicatechin glucoside
Zmhn001257	451.12	289.07	452.13	C21H24O11	[M-H]-	Flavonoids	Catechin-5-O-glucoside
pmd0160	454.29	313.27	453.29	C21H44NO7P	[M+H]+	Lipids	LysoPE 16:0(2n isomer)
pmb0876	454.29	313.27	453.29	C21H44NO7P	[M+H]+	Lipids	LysoPE 16:0
MWS5083	455.10	96.97	456.11	C17H21N4O9P	[M-H]-	Nucleotides and derivatives	Flavin Single Nucleotide(FMN)
Lmhn004756	455.10	233.04	456.11	C23H20O10	[M-H]-	Phenolic acids	Cinnamoylferuloyltartaric acid
Zmjn014062	455.35	455.35	456.36	C30H48O3	[M-H]-	Terpenoids	(23S)-3β-hydroxydammara-21-oic acid 21,23-lactone
pmb2654	460.10	117.90	461.15	C19H27NO12	[M-H]-	Phenolic acids	Anthranilate-1-O-Sophoroside
Lmzn001894	461.07	285.04	462.08	C21H18O12	[M-H]-	Flavonoids	Kaempferol-3-O-glucuronide
Lmhn002321	461.07	167.04	462.08	C21H18O12	[M-H]-	Phenolic acids	Vnilloylcaffeoyltartaric acid
Lmjp003655	463.12	301.08	462.12	C22H22O11	[M+H]+	Flavonoids	6-C-MethylKaempferol-3-glucoside*
Cmyp002064	463.12	301.08	462.12	C22H22O11	[M+H]+	Flavonoids	Yuanhuanin*
Hmmp004965	463.12	301.07	462.12	C22H22O11	[M+H]+	Flavonoids	Diosmetin-7-O-glucoside*
MWSslk013	463.12	301.09	462.12	C22H22O11	[M+H]+	Flavonoids	Tectoridin*
pmb3012	461.11	299.20	462.12	C22H22O11	[M-H]-	Flavonoids	Chrysoeriol-7-O-glucoside
pmp000579	463.12	301.07	462.12	C22H22O11	[M+H]+	Flavonoids	Diosmetin-7-O-galactoside*
MWSHY0195	463.12	301.08	462.12	C22H22O11	[M+H]+	Flavonoids	Hispidulin-7-O-Glucoside*
pmp001309	465.10	303.10	464.10	C21H20O12	[M+H]+	Flavonoids	6-Hydroxykaempferol-7-O-glucoside
Hmcn001884	463.09	301.03	464.10	C21H20O12	[M-H]-	Flavonoids	6-Hydroxyluteolin 5-glucoside
MWSHY0046	465.10	303.05	464.10	C21H20O12	[M+H]+	Flavonoids	Quercetin-3-O-glucoside*
Lmdp003286	465.10	303.06	464.10	C21H20O12	[M+H]+	Flavonoids	Isohyperoside*
MWSHY0113	465.10	303.06	464.10	C21H20O12	[M+H]+	Flavonoids	Quercetin-3-O-galactoside*
mws1329	463.09	301.03	464.10	C21H20O12	[M-H]-	Flavonoids	Quercetin-7-O-glucoside

Smgp004575	465.10	303.05	464.10	C21H20O12	[M+H]+	Flavonoids	Quercetin-5-O-β-D-glucoside*
Lmhp009464	466.29	325.27	465.29	C22H44NO7P	[M+H]+	Lipids	LysoPE 17:1(2n isomer)
Lmhp009769	466.29	325.27	465.29	C22H44NO7P	[M+H]+	Lipids	LysoPE 17:1
Xmsn002700	465.13	303.06	466.11	C21H22O12	[M-H]-	Flavonoids	Taxifolin-3'-O-glucoside
pmd0130	468.31	184.07	467.30	C22H46NO7P	[M+H]+	Lipids	LysoPC 14:0
Qmgp102003	468.31	184.07	467.30	C29H41NO4	[M+H]+	Alkaloids	O-Acetyljervine
pmb1912	474.17	327.00	473.17	C20H23N7O7	[M+H]+	Alkaloids	10-Formyltetrahydrofolic Acid
Lmhp008233	474.26	333.24	473.25	C23H40NO7P	[M+H]+	Lipids	LysoPE 18:4
Lmhp008801	476.28	335.26	475.27	C23H42NO7P	[M+H]+	Lipids	LysoPE 18:3
Lmhp008589	476.28	335.26	475.27	C23H42NO7P	[M+H]+	Lipids	LysoPE 18:3(2n isomer)
pmb0881	478.29	337.27	477.29	C23H44NO7P	[M+H]+	Lipids	LysoPE 18:2
pmb0874	478.29	337.27	477.29	C23H44NO7P	[M+H]+	Lipids	LysoPE 18:2(2n isomer)
Lmmp003783	479.09	303.05	478.08	C21H18O13	[M+H]+	Flavonoids	Quercetin-3-O-glucuronide
Lmqp003647	479.08	303.05	478.08	C21H18O13	[M+H]+	Flavonoids	Herbacetin-3-O-glucuronide
Lmjp003295	479.12	317.07	478.11	C22H22O12	[M+H]+	Flavonoids	6-Methoxykaempferol-3-O-glucoside*
Lmcp003788	479.12	317.06	478.11	C22H22O12	[M+H]+	Flavonoids	Isotamarixin
Hmcp002207	479.12	317.06	478.11	C22H22O12	[M+H]+	Flavonoids	Isorhamnetin-7-O-glucoside*
Hmgp002148	479.12	317.06	478.11	C22H22O12	[M+H]+	Flavonoids	Nepetin-7-O-alloside*
Hmgp002036	479.12	317.06	478.11	C22H22O12	[M+H]+	Flavonoids	Nepetin-7-O-glucoside*
mws0289	480.31	339.29	479.30	C23H46NO7P	[M+H]+	Lipids	LysoPE 18:1
pmb2260	480.31	184.07	479.30	C23H46NO7P	[M+H]+	Lipids	LysoPC 15:1
pmb0856	480.31	339.29	479.30	C23H46NO7P	[M+H]+	Lipids	LysoPE 18:1(2n isomer)
pmb0880	482.32	341.31	481.32	C23H48NO7P	[M+H]+	Lipids	LysoPE 18:0(2n isomer)
Lmhp009129	482.32	184.07	481.32	C23H48NO7P	[M+H]+	Lipids	LysoPC 15:0(2n isomer)
pmb0883	482.32	341.31	481.32	C23H48NO7P	[M+H]+	Lipids	LysoPE 18:0
pmb2319	482.32	184.07	481.32	C23H48NO7P	[M+H]+	Lipids	LysoPC 15:0

Lmsn011830	485.33	485.33	486.34	C30H46O5	[M-H]-	Terpenoids	2α,19α-Dihydroxy-3-oxours-12-en-28-oic acid
Lmmn003398	489.11	285.04	490.11	C23H22O12	[M-H]-	Flavonoids	Kaempferol-3-O-(6"-acetyl)glucoside
pmb0863	492.31	184.07	491.30	C24H46NO7P	[M+H]+	Lipids	LysoPC 16:2(2n isomer)
pmp001164	493.13	316.10	492.13	C23H24O12	[M+H]+	Flavonoids	Iristectorin B
pmb0736	493.13	331.08	492.13	C23H24O12	[M+H]+	Flavonoids	Tricin-7-O-Glucoside*
Lmgp003546	493.13	331.08	492.13	C23H24O12	[M+H]+	Flavonoids	4',5'-Dihydroxy-6,8-dimethoxyisoflavone-7-O-galactoside
Hmgp002238	493.14	331.08	492.13	C23H24O12	[M+H]+	Flavonoids	Jaceosidin-7-O-Glucoside*
pmb0790	494.19	332.50	493.18	C20H31NO13	[M+H]+	Others	Pyridoxine-5'-O-diglucoside
pmp001270	494.32	184.07	493.32	C24H48NO7P	[M+H]+	Lipids	LysoPC 16:1
Lmhp008833	494.32	184.07	493.32	C24H48NO7P	[M+H]+	Lipids	LysoPC 16:1(2n isomer)
Lmjp003231	495.11	333.07	494.11	C22H22O13	[M+H]+	Flavonoids	Patuletin-3-O-glucoside
Lmtn002997	493.10	331.05	494.11	C22H22O13	[M-H]-	Flavonoids	Mearnsetin-3-O-glucoside
pmd0132	496.34	184.07	495.33	C24H50NO7P	[M+H]+	Lipids	LysoPC 16:0(2n isomer)
pmb0855	496.34	184.07	495.33	C24H50NO7P	[M+H]+	Lipids	LysoPC 16:0
mad2394	501.10	337.05	502.11	C24H22O12	[M-H]-	Phenolic acids	Sinapoyl-p-coumaroyltartaric acid
Lmhp009802	504.31	363.29	503.30	C25H46NO7P	[M+H]+	Lipids	LysoPE 20:3(2n isomer)
Lmhp010040	504.31	363.29	503.30	C25H46NO7P	[M+H]+	Lipids	LysoPE 20:3
pmn001642	503.08	459.10	504.09	C23H20O13	[M-H]-	Flavonoids	Kaempferol-3-O-(2"-O-acetyl)glucuronide
mws1589	503.16	179.00	504.17	C18H32O16	[M-H]-	Others	D-Panose
MWS0442	527.16	365.10	504.17	C18H32O16	[M+Na]+	Others	Maltotriose
Lmhp010514	506.32	365.31	505.32	C25H48NO7P	[M+H]+	Lipids	LysoPE 20:2(2n isomer)
Lmhp008718	506.32	184.07	505.32	C25H48NO7P	[M+H]+	Lipids	LysoPC 17:2
Lmhp010757	506.32	365.31	505.32	C25H48NO7P	[M+H]+	Lipids	LysoPE 20:2
Hmln002199	505.10	300.03	506.11	C23H22O13	[M-H]-	Flavonoids	Quercetin-3-O-(6"-O-acetyl)galactoside
Lmhp009590	508.34	184.07	507.33	C25H50NO7P	[M+H]+	Lipids	LysoPC 17:1
Hmcp002187	509.13	347.07	508.12	C23H24O13	[M+H]+	Flavonoids	Limocitrin-3-O-galactoside*

Zmgp004436	509.13	347.08	508.12	C23H24O13	[M+H]+	Flavonoids	Okanin-4'-(6''-O-acetyl)glucoside*
Zmjp004852	509.13	347.08	508.12	C23H24O13	[M+H]+	Flavonoids	5,6,3',4'-Tetrahydroxy-3,7-dimethoxyflavone-6-O-glucoside *
HJAP006	509.13	347.08	508.12	C23H24O13	[M+H]+	Flavonoids	Syringetin-7-O-glucoside*
pmb2406	510.36	184.07	509.35	C25H52NO7P	[M+H]+	Lipids	LysoPC 17:0
Lmhp010515	510.36	184.07	509.35	C25H52NO7P	[M+H]+	Lipids	LysoPC 17:0(2n isomer)
Lmgp004518	531.15	177.05	512.13	C26H24O11	[M+H]+	Phenolic acids	CaffeoylferuloylQuinic acid
Lmhp009773	515.32	261.22	514.31	C27H46O9	[M+H]+	Lipids	1-α-Linolenoyl-glycerol-3-O-glucoside
Hmqp006235	516.30	184.07	515.30	C26H46NO7P	[M+H]+	Lipids	LysoPC 18:4
MWSmce328	517.13	163.04	516.13	C25H24O12	[M+H]+	Phenolic acids	Isochlorogenic acid C
Li512115	515.12	353.09	516.13	C25H24O12	[M-H]-	Phenolic acids	Isochlorogenic acid B
Wmzn002116	515.12	353.09	516.13	C25H24O12	[M-H]-	Phenolic acids	3,5-Dicaffeoylquinic acid
mws1584	515.12	191.05	516.13	C25H24O12	[M-H]-	Phenolic acids	1,3-O-Dicaffeoylquinic Acid
Lmhp010573	517.34	263.24	516.33	C27H48O9	[M+H]+	Lipids	1-Linoleoylglycerol-3-O-glucoside
pmb0854	518.32	184.07	517.32	C26H48NO7P	[M+H]+	Lipids	LysoPC 18:3
pmb0865	518.32	184.07	517.32	C26H48NO7P	[M+H]+	Lipids	LysoPC 18:3(2n isomer)
pmp001273	520.34	184.07	519.33	C26H50NO7P	[M+H]+	Lipids	LysoPC 18:2
pmp001251	520.34	184.07	519.33	C26H50NO7P	[M+H]+	Lipids	LysoPC 18:2(2n isomer)
pmn001644	519.08	300.00	520.09	C23H20O14	[M-H]-	Flavonoids	Quercetin-3-O-(2''-O-acetyl)glucuronide
pmp000682	538.22	359.14	520.17	C26H32O11	[M+NH4]+	Lignans and Coumarins	Matairesinol-4'-O-glucoside
Lmsp003655	521.20	131.05	520.19	C26H32O11	[M+H]+	Phenolic acids	Dehydrodiconiferyl alcohol-gamma'-O-glucoside*
Cmsp003083	521.20	131.05	520.19	C26H32O11	[M+H]+	Others	Dehydrodiconiferyl alcohol-4-O-glucoside*
pmp001281	522.36	184.07	521.35	C26H52NO7P	[M+H]+	Lipids	LysoPC 18:1
Lmhp010190	522.36	184.07	521.35	C26H52NO7P	[M+H]+	Lipids	LysoPC 18:1(2n isomer)
pmn001710	521.13	359.08	522.14	C24H26O13	[M-H]-	Phenolic acids	Rosmarinic acid-3'-O-glucoside
Zmgp005070	523.14	361.09	522.14	C24H26O13	[M+H]+	Flavonoids	Centaurein

Lmmn002274	521.20	359.15	522.21	C26H34O11	[M-H]-	Lignans and Coumarins	Isolariciresinol-9'-O-glucoside*
Lmyn003971	521.20	359.15	522.21	C26H34O11	[M-H]-	Lignans and Coumarins	Dihydrodehydrodiconiferyl alcohol-4-O-glucoside*
pmd0136	524.37	184.07	523.36	C26H54NO7P	[M+H]+	Lipids	LysoPC 18:0(2n isomer)
mws0126	524.37	184.07	523.36	C26H54NO7P	[M+H]+	Lipids	LysoPC 18:0
Lmtn003096	523.21	361.14	524.23	C26H36O11	[M-H]-	Lignans and Coumarins	Secoisolariciresinol 4-O-glucoside
Lmjp003731	531.15	177.06	530.14	C26H26O12	[M+H]+	Phenolic acids	3,4-O-Dicaffeoylquinic Acid Methyl Ester
Lmhp008924	532.34	184.07	531.33	C27H50NO7P	[M+H]+	Lipids	LysoPC 19:3
pmp000587	535.11	449.10	534.10	C24H22O14	[M+H]+	Flavonoids	Luteolin-7-O-(6"-malonyl)glucoside
Lmdp004892	535.11	287.05	534.10	C24H22O14	[M+H]+	Flavonoids	Kaempferol-3-O-(6"-malonyl)galactoside
Zmfn000481	535.04	323.03	536.04	C14H22N2O16P2	[M-H]-	Nucleotides and derivatives	Uridine-5'-Diphosphate-D-Xylose
Cmxp004324	537.12	289.07	536.12	C24H24O14	[M+H]+	Flavonoids	Okanin-4'-O-(6"-O-malonyl)glucoside
Lmdn001925	537.20	375.15	538.21	C26H34O12	[M-H]-	Lignans and Coumarins	Olivil-4'-O-glucoside
pmc0960	544.34	184.07	543.33	C28H50NO7P	[M+H]+	Lipids	LysoPC 20:4
Lmhp009890	546.36	184.07	545.35	C28H52NO7P	[M+H]+	Lipids	LysoPC 20:3
pmd0146	548.37	184.07	547.36	C28H54NO7P	[M+H]+	Lipids	LysoPC 20:2(2n isomer)
pmd0147	548.37	184.07	547.36	C28H54NO7P	[M+H]+	Lipids	LysoPC 20:2
pmb0608	549.12	301.40	548.12	C25H24O14	[M+H]+	Flavonoids	Chrysoeriol-7-O-(6"-malonyl)glucoside
pmp000588	549.12	463.12	548.12	C25H24O14	[M+H]+	Flavonoids	Diosmetin-7-O-(6"-malonyl)glucoside
pmp000589	551.10	303.05	550.10	C24H22O15	[M+H]+	Flavonoids	Quercetin-7-O-(6"-malonyl)glucoside
Lmmn002260	551.21	389.17	552.22	C27H36O12	[M-H]-	Lignans and Coumarins	5'-Methoxyisolariciresinol-9'-O-glucoside
pma0702	558.28	207.07	557.27	C29H39N3O8	[M+H]+	Alkaloids	N1,N8-Bis(sinapoyl)spermidine
Lmhn003802	561.13	337.06	562.13	C26H26O14	[M-H]-	Phenolic acids	Sinapoylsinapoyltartaric acid
Rfmb25702	561.20	357.13	562.21	C28H34O12	[M-H]-	Lignans and Coumarins	Pinoresinol-4-O-(6"-acetyl)glucoside
HJAP064	565.12	317.08	564.09	C25H24O15	[M+H]+	Flavonoids	Isorhamnetin-3-O-(6"-malonyl)glucoside*
Zmhp005139	565.12	317.07	564.11	C25H24O15	[M+H]+	Flavonoids	Tamarixetin-3-O-(6"-malonyl)glucoside*
pmb2922	565.05	323.10	566.06	C15H24N2O17P2	[M-H]-	Nucleotides and derivatives	Uridine 5'-diphospho-D-glucose

Zmxp005470	579.13	331.08	578.13	C26H26O15	[M+H]+	Flavonoids	Tricin-7-O-(6''-O-malonyl)glucoside
Lmgp004474	579.17	271.06	578.16	C27H30O14	[M+H]+	Flavonoids	Genistein-7-O-galactoside-rhamnose*
Hmcp001769	581.15	303.04	580.14	C26H28O15	[M+H]+	Flavonoids	Quercetin-3-O-rhamnosyl(1→2)arabinoside
Zmhn001446	579.21	417.16	580.22	C28H36O13	[M-H]-	Lignans and Coumarins	Syringaresinol-4'-O-glucoside
pmb0492	584.28	325.00	583.27	C34H37N3O6	[M+H]+	Alkaloids	N',N'',N'''-p-Coumaroyl-cinnamoyl-caffeoyl spermidine
Rfmb056	591.21	387.14	592.22	C29H36O13	[M-H]-	Lignans and Coumarins	Medioresinol-4'-O-(6'''-acetyl)glucoside
MWSHY0061	595.16	287.06	594.16	C27H30O15	[M+H]+	Flavonoids	Kaempferol-3-O-neohesperidoside*
Lmsn002815	593.15	285.04	594.16	C27H30O15	[M-H]-	Flavonoids	Kaempferol-3-O-rutinoside
pmp001106	595.17	577.16	594.16	C27H30O15	[M+H]+	Flavonoids	Vitexin-2''-O-glucoside
MWSHY0080	595.17	287.06	594.16	C27H30O15	[M+H]+	Flavonoids	Luteolin-7-O-neohesperidoside*
Lmsp004670	595.17	287.06	594.16	C27H30O15	[M+H]+	Flavonoids	Kaempferol-3-O-glucoside-7-O-rhamnoside*
mws1073	595.17	457.20	594.16	C27H30O15	[M+H]+	Flavonoids	Apigenin-6,8-di-C-glucoside (Vicenin-2)
Lmjp002596	597.14	303.05	596.14	C26H28O16	[M+H]+	Flavonoids	Quercetin-3-O-sambubioside
Lmmp000897	611.14	287.05	610.13	C30H26O14	[M+H]+	Flavonoids	Gallocatechin-(4α→8)-gallocatechin
pmn001583	609.15	300.00	610.15	C27H30O16	[M-H]-	Flavonoids	Quercetin-3-O-robinobioside
Lmsp004166	611.16	303.05	610.15	C27H30O16	[M+H]+	Flavonoids	Quercetin-3-O-glucoside-7-O-rhamnoside
pmb0665	611.16	465.10	610.15	C27H30O16	[M+H]+	Flavonoids	Orientin-7-O-glucoside
mws0059	609.15	301.00	610.15	C27H30O16	[M-H]-	Flavonoids	Quercetin-3-O-rutinoside (Rutin)
pme0001	609.18	301.00	610.19	C28H34O15	[M-H]-	Flavonoids	3',5,7-Trihydroxy-4'-Methoxyflavanone-7-O-neohesperidoside
mws4134	611.14	306.08	612.15	C20H32N6O12S2	[M-H]-	Amino acids and derivatives	Oxiglutatione
Cmxp003531	613.18	289.07	612.17	C27H32O16	[M+H]+	Flavonoids	Okanin-4'-O-gentiobioside
XLn05122	615.14	191.06	616.14	C29H28O15	[M-H]-	Phenolic acids	1,4-O-dicaffeoyl-3-O-succinoyl-quinic acid*
XLn05515	615.14	191.06	616.14	C29H28O15	[M-H]-	Phenolic acids	1,5-O-dicaffeoyl-3-O-succinoyl-quinic acid*
Rfmb26201	621.22	417.16	622.23	C30H38O14	[M-H]-	Lignans and Coumarins	Syringaresinol-4'-O-(6''-acetyl)glucoside
Zmxp002867	625.17	301.07	624.17	C28H32O16	[M+H]+	Flavonoids	Chrysoeriol-5,7-di-O-glucoside

HJN051	623.16	315.05	624.17	C28H32O16	[M-H]-	Flavonoids	Tamarixetin-3-O-rutinoside
Lmhp003217	625.17	317.06	624.17	C28H32O16	[M+H]+	Flavonoids	2'-Hydoxy,5-methoxyGenistein-O-rhamnosyl-glucoside*
Lmmp002463	625.18	317.07	624.17	C28H32O16	[M+H]+	Flavonoids	Sexangularetin-3-O-glucoside-7-O-rhamnoside*
MWSHY0064	625.17	317.07	624.17	C28H32O16	[M+H]+	Flavonoids	Isorhamnetin-3-O-neohesperidoside*
Lmsp004721	625.18	317.07	624.17	C28H32O16	[M+H]+	Flavonoids	Tamarixetin-3-O-glucoside-7-O-rhamnoside*
Lmnp002584	625.18	463.13	624.17	C28H32O16	[M+H]+	Flavonoids	Hispidulin-8-C-(2''-O-glucosyl)glucoside*
HJAP026	625.18	463.13	624.17	C28H32O16	[M+H]+	Flavonoids	Chrysoeriol-6-C-glucoside-4'-O-glucoside*
Hmgp001944	625.21	163.03	624.21	C29H36O15	[M+H]+	Phenolic acids	Isoacteoside
Zmdp003457	627.16	319.05	626.15	C27H30O17	[M+H]+	Flavonoids	Myricetin-3-O-galactoside-3'-O-rhamnoside*
pmp001310	627.16	303.10	626.15	C27H30O17	[M+H]+	Flavonoids	6-Hydroxykaempferol-3,6-O-Diglucoside*
MWSHY0162	627.16	303.06	626.15	C27H30O17	[M+H]+	Flavonoids	Quercetin-3-O-sophoroside (Baimaside)*
Lmsp003729	627.16	319.05	626.15	C27H30O17	[M+H]+	Flavonoids	Myricetin-3-O-rutinoside*
pmp000596	627.16	465.10	626.15	C27H30O17	[M+H]+	Flavonoids	Quercetin-3-O-(2''-O-galactosyl)glucoside
Zmcp002666	627.14	303.05	626.15	C27H30O17	[M+H]+	Flavonoids	Quercetin-3,7-Di-O-glucoside
HJN038	631.10	317.04	632.10	C28H24O17	[M-H]-	Flavonoids	Myricetin-3-O-(6''-galloyl)glucoside
XLn05042	631.14	469.11	632.14	C29H28O16	[M-H]-	Phenolic acids	1,4-O-dicaffeoyl-3-O-maloyl-quinic acid*
XLn04840	631.14	469.11	632.14	C29H28O16	[M-H]-	Phenolic acids	1,5-O-dicaffeoyl-3-O-maloyl-quinic acid*
Lmmp002560	641.13	303.05	640.13	C27H28O18	[M+H]+	Flavonoids	Quercetin-3-O-(2''-O-glucosyl)glucuronide
Hmcp001578	641.17	479.11	640.16	C28H32O17	[M+H]+	Flavonoids	Isorhamnetin-3,7-O-diglucoside
Lmsp004749	655.19	347.08	654.18	C29H34O17	[M+H]+	Flavonoids	Syringetin-3-O-rutinoside
Lmgp003803	655.19	331.08	654.18	C29H34O17	[M+H]+	Flavonoids	5,7,4'-Trihydroxy-6,8-dimethoxyisoflavone-7-O-galactoside -rhamnose
pmb0713	655.19	331.08	654.18	C29H34O17	[M+H]+	Flavonoids	Tricin-7-O-(2''-O-glucosyl)glucoside*
XLn05373	659.17	497.13	660.17	C31H32O16	[M-H]-	Phenolic acids	1,5-O-dicaffeoyl-3-O-dimethylmalyl-quinic acid
mws4163	665.21	485.30	666.22	C24H42O21	[M-H]-	Others	Nystose
mws1593	665.21	161.00	666.22	C24H42O21	[M-H]-	Others	D-Maltotetraose

Lmqn000213	665.21	341.11	666.22	C24H42O21	[M-H]-	Others	Stachyose
Lmxp010913	676.45	184.08	675.45	C35H66NO9P	[M+H]+	Lipids	1-(9Z-Octadecenoyl)-2-sn-glycero-3-phosphocholine
Lmyn005812	675.36	397.14	676.37	C33H56O14	[M-H]-	Lipids	Gingerglycolipid A
Lmhp008744	677.37	261.22	676.37	C33H56O14	[M+H]+	Lipids	1-α-Linolenoyl-glycerol-2,3-di-O-glucoside
pmp001276	677.37	677.37	676.37	C33H56O14	[M+H]+	Lipids	1-Linolenoyl-rac-glycerol-diglucoside
Lmhp008513	677.37	261.22	676.37	C33H56O14	[M+H]+	Lipids	2-α-Linolenoyl-glycerol-1,3-di-O-glucoside
HJN102	677.17	515.14	678.16	C34H30O15	[M-H]-	Phenolic acids	3,4,5-Tricaffeoylquinic acid
Lmgn002250	677.17	191.06	678.18	C31H34O17	[M-H]-	Phenolic acids	Dicaffeoylquinic acid-O-glucoside
XLn04217	677.18	515.14	678.18	C31H34O17	[M-H]-	Phenolic acids	1,5-O-dicaffeoyl-3-O-glucoside-quinic acid
Lmyn006011	677.38	397.13	678.38	C33H58O14	[M-H]-	Lipids	Gingerglycolipid B
Lmmn004145	675.41	397.14	679.42	C38H60O10	[M-H]-	Terpenoids	3-O-(2-O-Acetyl-glucosyl)oleanolic acid
Lmyn006221	679.39	397.13	680.40	C33H60O14	[M-H]-	Lipids	Gingerglycolipid C
pmn001370	681.24	519.19	682.25	C32H42O16	[M-H]-	Lignans and Coumarins	Pinoresinol-4,4'-O-di-O-glucoside
MWSmce499	685.27	523.22	686.28	C32H46O16	[M-H]-	Lignans and Coumarins	Secoisolariciresinol diglucoside
Lmgn002253	695.20	353.09	696.19	C31H36O18	[M-H]-	Phenolic acids	Syringoylcaffeoylquinic acid-D-glucose
pmb0709	713.16	465.20	712.15	C30H32O20	[M+H]+	Flavonoids	Quercetin-7-O-(6"-malonyl)glucosyl-5-O-glucoside
pmb0706	713.16	465.10	712.15	C30H32O20	[M+H]+	Flavonoids	Quercetin-3-O-(6"-O-malonyl)glucosyl-5-O-glucoside
XLn05670	715.16	353.09	716.16	C33H32O18	[M-H]-	Phenolic acids	3,5-Di-O-caffeoyl-1-O-(2,4-disuccinoyl)-quinic acid
Lmgn004359	721.18	353.09	722.21	C33H38O18	[M-H]-	Phenolic acids	SinapoylcaffeoylQuinic acid O-glucose
Hmcp001658	727.17	479.11	726.16	C31H34O20	[M+H]+	Flavonoids	Isorhamnetin-3-O-(6"-malonyl)glucoside-7-O-glucoside
pme2651	742.10	620.10	743.08	C21H28N7O17P3	[M-H]-	Nucleotides and derivatives	NADP (Nicotinamide adenine dinucleotide phosphate)
XLn04903	747.15	585.12	748.15	C33H32O20	[M-H]-	Phenolic acids	3,5-Di-O-caffeoyl-1-O-(2,4-dimaloyl)-quinic acid
Lmsp003161	757.22	287.06	756.21	C33H40O20	[M+H]+	Flavonoids	Kaempferol-3-O-sophoroside-7-O-rhamnoside
HJAP127	757.22	303.05	756.21	C33H40O20	[M+H]+	Flavonoids	Quercetin-3-O-(2"-O-Rhamnosyl)rutinoside
pmn001505	763.43	763.40	764.44	C41H64O13	[M-H]-	Terpenoids	Oleanolic acid-3-O-xylosyl(1→3)glucuronide
Lmmp002334	773.21	303.05	772.21	C33H40O21	[M+H]+	Flavonoids	Quercetin-3-O-rutinoside-7-O-glucoside*

Zmhp002640	773.21	303.05	772.21	C33H40O21	[M+H]+	Flavonoids	6-Hydroxykaempferol-3-O-Rutinoside-6-O-glucoside*
Lmsp003790	787.23	317.07	786.22	C34H42O21	[M+H]+	Flavonoids	Isorhamnetin-3-O-sophoroside-7-O-rhamnoside
XLn05839	793.17	631.15	794.17	C38H34O19	[M-H]-	Phenolic acids	3,5-Di-O-caffeoyl-1-O-(2-O-caffeoylmaloyl)-quinic acid
XLn04478	793.19	631.16	794.19	C35H38O21	[M-H]-	Phenolic acids	3,5-Di-O-caffeoyl-1-O-(2-O-glucosylmaloyl)-quinic acid*
XLn04250	793.19	631.16	794.19	C35H38O21	[M-H]-	Phenolic acids	3,5-Di-O-caffeoyl-1-O-(4-O-glucosylmaloyl)-quinic acid*
Lmdn000248	827.27	665.22	828.28	C30H52O26	[M-H]-	Others	Verbascose
XLn05736	909.18	747.15	910.18	C42H38O23	[M-H]-	Phenolic acids	3,4,5-Tri-O-caffeoyl-1-O-(2,4-dimaloyl)-quinic acid
Lmsp003853	919.25	303.05	918.24	C42H46O23	[M+H]+	Flavonoids	Quercetin-3-O-sophoroside-7-O-rhamnoside
Lmwp004293	935.25	303.05	934.24	C42H46O24	[M+H]+	Flavonoids	Quercetin-3-O-sophoroside-7-O-glucoside

Table S2. Differential metabolites in the CK_vs_T1 , CK_vs_T2, CK_vs_T3, and CK_vs_T4.

Index	Formula	Compounds	Class	CK_vs_T1	CK_vs_T2	CK_vs_T3	CK_vs_T4
ML10198895	C3H7NO2	N-Methylglycine	Amino acids and derivatives	up	FALSE	FALSE	up
pme3033	C4H9NO2	N,N-Dimethylglycine	Amino acids and derivatives	FALSE	up	FALSE	up
pme0010	C3H7NO3	L-Serine	Amino acids and derivatives	FALSE	FALSE	up	up
mws0147	C5H10O3	β-Hydroxyisovaleric acid	Organic acids	down	FALSE	FALSE	FALSE
Lmbp000728	C8H8O	(S)-2-Phenyloxirane	Others	up	up	up	FALSE
pme0195	C3H7NO2S	L-Cysteine	Amino acids and derivatives	down	down	FALSE	down
mws0133	C6H6N2O	Nicotinamide	Others	FALSE	up	FALSE	FALSE
MWS2091	C8H10O	1-Phenylethanol	Phenolic acids	FALSE	FALSE	up	FALSE
pme0490	C6H5NO2	Nicotinic acid (Vitamin B3)	Others	FALSE	up	FALSE	FALSE

Lmbn000612	C5H7NO3	1-Pyrroline-4-hydroxy-2-carboxylic acid	Organic acids	FALSE	up	FALSE	FALSE
MWSmce709	C9H7N	Isoquinoline	Alkaloids	FALSE	up	up	FALSE
MWS0811	C6H11NO2	L-Pipecolic Acid	Organic acids	up	FALSE	FALSE	up
pme2693	C6H14N2O	N-Acetylputrescine	Alkaloids	FALSE	down	FALSE	FALSE
Zmpn000638	C4H9N3O2	3-Guanidinopropionic acid	Organic acids	up	FALSE	FALSE	up
mws0473	C5H8O4	2-Methylsuccinic acid*	Organic acids	FALSE	FALSE	FALSE	up
pme0243	C5H8O4	Glutaric acid*	Organic acids	down	FALSE	down	FALSE
Lmrn002746	C6H12O3	2-Hydroxy-4-methylpentanoic acid	Organic acids	down	FALSE	down	FALSE
mws0889	C4H8O5	D-Threonic Acid	Others	FALSE	FALSE	FALSE	up
pme0033	C5H4N4O	Hypoxanthine	Nucleotides and derivatives	FALSE	up	FALSE	FALSE
MWSmce466	C8H8O2	4-Hydroxyacetophenone*	Phenolic acids	FALSE	FALSE	down	down
pme1724	C8H8O2	Benzoic acid methyl ester	Phenolic acids	up	FALSE	FALSE	FALSE
MWS1848	C8H8O2	Phenyl acetate*	Phenolic acids	down	FALSE	down	down
MWSmce699	C9H12O	(Ethoxymethyl)benzene	Others	FALSE	FALSE	FALSE	down
pme1002	C8H11NO	L-Tyramine	Alkaloids	up	up	up	FALSE
mws0749	C7H6O3	4-Hydroxybenzoic acid*	Phenolic acids	up	up	FALSE	up
Hmgn001653	C7H6O3	Protocatechualdehyde*	Phenolic acids	up	up	FALSE	up
pme2828	C6H5NO3	4-Nitrophenol	Phenolic acids	down	down	down	down
Qmkp093004	C9H7NO	Indole-3-carboxaldehyde	Alkaloids	down	FALSE	FALSE	down
HX1365	C9H6O2	Coumarin	Lignans and Coumarins	up	FALSE	FALSE	up
Zmzn000113	C5H9NO4	L-threo-3-Methylaspartate	Amino acids and derivatives	up	FALSE	FALSE	FALSE
Lmbn000216	C5H8O5	3-Methylmalic acid*	Organic acids	up	FALSE	up	up
Zmyn000247	C5H8O5	2-Hydroxyglutaric Acid*	Organic acids	FALSE	FALSE	FALSE	up
Zmyn000230	C5H8O5	2-Dehydro-3-deoxy-L-arabinonate*	Others	FALSE	FALSE	up	up

pmb2826	C5H8O5	L-Citramalic acid	Organic acids	up	FALSE	up	up
MWSmce698	C10H12O	2',4'-Dimethylacetophenone	Others	FALSE	FALSE	FALSE	down
MWSmce463	C10H12O	Mesitaldehyde	Others	FALSE	FALSE	FALSE	down
Lmgp000796	C8H7NO2	4-Hydroxymandelonitrile	Alkaloids	FALSE	FALSE	FALSE	up
pme1210	C5H11NO2S	L-Methionine	Amino acids and derivatives	FALSE	FALSE	FALSE	up
Zmgn000173	C5H10O5	D-Ribose	Others	up	FALSE	up	up
pme1109	C5H5N5O	Guanine	Nucleotides and derivatives	up	up	up	up
mws0183	C7H6O4	3,4-Dihydroxybenzoic acid (Protocatechuic acid)*	Phenolic acids	up	up	FALSE	up
mws0180	C7H6O4	2,5-Dihydroxybenzoic acid; Gentisic Acid*	Phenolic acids	up	up	FALSE	up
pmb0819	C10H8N2	3-Indoleacetonitrile	Alkaloids	down	FALSE	FALSE	FALSE
mws0005	C10H12N2	Tryptamine	Alkaloids	down	FALSE	FALSE	down
pme2914	C6H10O5	3-Hydroxy-3-methylpentane-1,5-dioic acid	Amino acids and derivatives	FALSE	FALSE	FALSE	up
MWSmce712	C10H12O2	Ethyl phenylacetate	Phenolic acids	up	FALSE	up	FALSE
MWS0274	C9H10O3	DL-3-Phenyllactic acid*	Organic acids	down	FALSE	FALSE	down
NK10253223	C8H9NO3	2-Amino-3-methoxybenzoic acid	Phenolic acids	FALSE	down	down	down
Zmjp000624	C8H9NO3	Pyridoxal	Others	FALSE	down	down	down
MWSmce501	C8H8O4	Protocatechuic Acid Methyl Ester	Phenolic acids	up	FALSE	up	up
pme1383	C8H11NO3	Pyridoxine	Others	down	FALSE	FALSE	down
ML10176345	C7H8O5	3-Dehydroshikimic acid	Organic acids	up	FALSE	FALSE	FALSE
pme0253	C8H15NO3	N-Acetyl-L-leucine	Amino acids and derivatives	FALSE	FALSE	FALSE	up
pme3009	C6H6O6	Trans-Citridic acid	Organic acids	down	FALSE	FALSE	down
MA10039492	C6H6O6	Dehydroascorbic acid	Others	up	up	up	FALSE

mws0154	C7H10O5	Shikimic acid	Organic acids	FALSE	FALSE	FALSE	up
Zmyn000155	C7H14N2O3	N-α-Acetyl-L-ornithine	Amino acids and derivatives	up	FALSE	FALSE	FALSE
pme2559	C6H9NO5	N-Acetyl-L-Aspartic Acid	Amino acids and derivatives	up	up	up	up
pmb0813	C10H9NO2	1-Methoxyindole-3-carbaldehyde	Alkaloids	up	up	FALSE	FALSE
mws4175	C6H8O6	D-Glucurono-6,3-lactone	Others	FALSE	FALSE	FALSE	down
mws1013	C9H6O4	Esculetin	Lignans and Coumarins	FALSE	up	FALSE	FALSE
pmb2795	C10H10O3	4-Methoxycinnamic acid	Phenolic acids	up	up	FALSE	FALSE
mws0981	C6H5N5O2	Isoxanthopterin	Nucleotides and derivatives	down	FALSE	down	down
mws2212	C9H8O4	Caffeic acid	Phenolic acids	up	up	up	up
mws1164	C6H12O6	D-Fructose*	Others	up	FALSE	FALSE	FALSE
mws4170	C6H12O6	D-Glucose*	Others	up	FALSE	FALSE	FALSE
Hmln000297	C6H12O6	Inositol*	Others	up	FALSE	FALSE	FALSE
mws0093	C10H12O3	Coniferyl alcohol	Phenolic acids	FALSE	up	FALSE	up
MWS2070	C10H12O3	Propyl 4-hydroxybenzoate	Phenolic acids	down	FALSE	down	down
pmb1754	C5H15NO4P+	O-Phosphocholine	Alkaloids	up	up	up	up
Hmmp001310	C11H9NO2	3-Indoleacrylic acid	Alkaloids	down	FALSE	FALSE	FALSE
pme0137	C7H12N2O4	N-Acetyl-L-Glutamine	Amino acids and derivatives	up	down	FALSE	up
pme0075	C7H11NO5	N-Acetyl-L-glutamic acid	Amino acids and derivatives	up	FALSE	FALSE	up
pme2244	C11H11NO2	3-Indolepropionic acid	Alkaloids	down	FALSE	FALSE	down
mws0597	C10H9NO3	5-Hydroxyindole-3-acetic acid	Alkaloids	FALSE	FALSE	up	FALSE
Zmyn000453	C6H8O7	Isocitric Acid	Organic acids	up	up	up	FALSE

Lmmp002080	C11H16N2O	N-(4-Aminobutyl)benzamide	Alkaloids	up	FALSE	FALSE	up
pme3705	C6H10O7	D-Glucoronic acid	Others	FALSE	FALSE	FALSE	up
XLn05261	C9H8O5	Salicylacetic acid	Phenolic acids	up	up	up	up
Zmzn000079	C4H9O7P	D-Erythrose-4-phosphate	Others	up	up	up	up
Zmbp002538	C11H12N2O2	1-Methoxy-indole-3-acetamide	Alkaloids	down	FALSE	FALSE	down
mws0282	C11H12N2O2	L-Tryptophan	Amino acids and derivatives	down	FALSE	FALSE	down
pmb0818	C11H11NO3	Methoxyindoleacetic acid	Alkaloids	down	FALSE	FALSE	down
MWS0618	C9H19NO4	Pantothenol	Others	FALSE	FALSE	down	FALSE
pme3443	C11H12O4	Sinapinaldehyde	Phenolic acids	up	up	up	up
pme1654	C12H18O3	Jasmonic acid	Organic acids	up	up	FALSE	FALSE
MWS1900	C11H20O4	Undecanedioic acid	Lipids	down	FALSE	FALSE	FALSE
mws5045	C12H24O3	12-Hydroxydodecanoic acid	Lipids	down	FALSE	down	down
pme1228	C11H12N2O3	5-Hydroxy-L-tryptophan	Amino acids and derivatives	down	FALSE	down	down
Lmtp005226	C15H24O	Santalol	Others	FALSE	FALSE	FALSE	down
MWS20178	C11H10O5	isofraxidin	Lignans and Coumarins	down	FALSE	FALSE	FALSE
mws0520	C11H13NO4	N-Acetyl-L-tyrosine	Amino acids and derivatives	FALSE	down	FALSE	up
pme0220	C13H20O3	Methyl jasmonate	Organic acids	FALSE	FALSE	FALSE	down
HJAP051	C12H18O4	3-(3,4,5-Trimethoxyphenyl)propan-1-ol	Phenolic acids	up	FALSE	FALSE	FALSE
pma6455	C5H11O8P	Ribulose-5-phosphate	Others	up	up	up	up
mws0474	C12H22O4	Dodecanedioic aicd	Organic acids	down	FALSE	down	down
Lmhp001670	C11H22N2O3	L-Valyl-L-Leucine	Amino acids and derivatives	FALSE	up	FALSE	up
Lmjp007763	C15H20O2	6,7-Dehydroartemisinic acid	Terpenoids	up	FALSE	up	up

pmb0962	C10H22N2O4	L-Lysine-Butanoic Acid	Amino acids and derivatives	FALSE	down	FALSE	down
Jmbn003202	C10H20O6	Butyl Beta-D-Fructopyranoside	Others	up	up	up	up
Lmcp006516	C15H24O2	Baimuxinal	Terpenoids	FALSE	FALSE	FALSE	down
pme0264	C10H14N2O5	Thymidine	Nucleotides and derivatives	down	FALSE	FALSE	FALSE
MWS2430	C15H30O2	13-methylmyristic acid	Lipids	FALSE	down	FALSE	down
pme3732	C9H13N3O5	Cytidine	Nucleotides and derivatives	FALSE	FALSE	FALSE	down
ML10180524	C9H13N3O5	Cytarabine	Nucleotides and derivatives	FALSE	up	FALSE	FALSE
mws0248	C9H12N2O6	Uridine	Nucleotides and derivatives	FALSE	up	FALSE	FALSE
Lmhp002031	C12H24N2O3	L-Leucyl-L-Leucine	Amino acids and derivatives	FALSE	up	up	up
MWSmce062	C15H18O3	Xanthatin	Terpenoids	FALSE	FALSE	FALSE	down
Hmcn003273	C15H20O3	Reynosin	Terpenoids	up	up	up	up
Jmcn007352	C15H22O3	5α-Hydroxycostic acid	Terpenoids	up	up	up	up
pme3961	C10H13N5O3	2'-Deoxyadenosine	Nucleotides and derivatives	FALSE	up	FALSE	FALSE
Lmcn009122	C16H30O2	(7Z)-Hexadecenoic acid	Lipids	FALSE	FALSE	down	FALSE
MWSHY0124	C15H12O4	Pinocembrin (Dihydrochrysin)	Flavonoids	down	down	down	down
mws0120	C8H20NO6P	Choline Alfoscerate	Lipids	up	up	up	up
Zmyn000110	C6H14NO8P	D-Glucosamine 1-phosphate	Others	up	up	up	up
mws0866	C6H13O9P	D-Glucose 6-phosphate*	Others	up	up	up	up
MWS2442	C6H13O9P	D-Fructose 6-Phosphate*	Others	up	up	up	up

Zmpn000095	C6H15O9P	Sorbitol-6-phosphate	Others	up	up	up	up
Lmhp001732	C14H18N2O3	L-Prolyl-L-Phenylalanine	Amino acids and derivatives	FALSE	FALSE	up	up
mws0715	C13H16N2O4	Phenylacetyl-L-glutamine	Amino acids and derivatives	down	FALSE	down	FALSE
Lmhp002001	C14H20N2O3	L-Valyl-L-Phenylalanine	Amino acids and derivatives	FALSE	FALSE	FALSE	up
Zmgn001039	C9H17NO6S	S-Ribosyl-L-homocysteine	Amino acids and derivatives	FALSE	FALSE	down	FALSE
pme0230	C10H13N5O4	Adenosine*	Nucleotides and derivatives	FALSE	FALSE	down	FALSE
mws0037	C16H12O4	Formononetin (7-Hydroxy-4'-methoxyisoflavone)	Flavonoids	down	down	down	down
mws1060	C10H12N4O5	9-(Arabinosyl)hypoxanthine	Nucleotides and derivatives	up	up	up	FALSE
MWS20151	C15H10O5	Apigenin; 4',5,7-Trihydroxyflavone	Flavonoids	FALSE	FALSE	up	FALSE
Lmyn006227	C15H10O5	3,5,7-Trihydroxyflavone	Flavonoids	FALSE	FALSE	up	FALSE
mws1297	C13H18O6	Benzyl glucoside	Phenolic acids	FALSE	FALSE	FALSE	down
pmb0034	C10H16N2O7	L-α-Glutamyl-L-Glutamic Acid	Amino acids and derivatives	down	FALSE	down	down
pmb1574	C18H28O2	9,12-Octadecadien-6-Ynoic Acid	Lipids	down	up	FALSE	FALSE
Wmln002759	C14H17NO5	4-(Rhamnosyloxy)phenylacetonitrile	Alkaloids	down	down	down	FALSE
Hmhp001812	C11H15N5O4	2'-O-Methyladenosine	Nucleotides and derivatives	FALSE	FALSE	up	FALSE
YC512118	C18H35NO	Oleamide (9-Octadecenamide)	Lipids	FALSE	FALSE	FALSE	up
pme1178	C10H13N5O5	Guanosine	Nucleotides and derivatives	FALSE	up	FALSE	FALSE

MWSmce089	C17H17NO3	p-Coumaroyltyramine	Alkaloids	FALSE	down	FALSE	down
mws0129	C16H12O5	Apigenin 7-methyl ether*	Flavonoids	down	FALSE	FALSE	down
mws0051	C16H12O5	Acacetin*	Flavonoids	down	FALSE	FALSE	down
mws0918	C16H12O5	Prunetin (5,4'-Dihydroxy-7-methoxyisoflavone)	Flavonoids	down	FALSE	FALSE	down
mws4160	C16H12O5	5,7-Dihydroxy-8-Methoxyflavone*	Flavonoids	down	FALSE	FALSE	down
mws1505	C17H16O4	Phenethyl caffeate	Phenolic acids	down	down	FALSE	down
Hmbp002730	C15H10O6	Isoscutellarein*	Flavonoids	up	FALSE	up	FALSE
Lmmp004504	C15H10O6	2'-Hydroxygenistein*	Flavonoids	FALSE	FALSE	up	FALSE
MWSHY0058	C15H10O6	Luteolin (5,7,3',4'-Tetrahydroxyflavone)*	Flavonoids	FALSE	FALSE	up	FALSE
pmp000727	C15H17N3O3	Feruloylhistamine	Alkaloids	FALSE	FALSE	FALSE	down
Lmsp004137	C15H12O6	3,4,2',4',6'-Pentahydroxychalcone	Flavonoids	FALSE	FALSE	FALSE	down
mws1496	C17H23NO3	L-Hyoscyamine	Alkaloids	down	FALSE	FALSE	FALSE
pmb2787	C18H30O3	9-Oxo-10E,12Z-octadecadienoic acid	Lipids	FALSE	up	FALSE	FALSE
Lmqp000329	C8H14N3O7P	5-Aminoimidazole ribonucleotide	Nucleotides and derivatives	up	up	up	up
Lmhn102068	C13H12O8	2-O-Caffeoylmalic acid	Phenolic acids	down	FALSE	FALSE	FALSE
Hmqp005411	C18H32O3	9-Oxo-12Z-Octadecenoic acid	Lipids	FALSE	FALSE	down	FALSE
Hmqp005455	C18H32O3	15(R)-Hydroxylinoleic Acid	Lipids	FALSE	FALSE	down	FALSE
Zmhn001358	C13H16O8	4-O-Glucosyl-4-hydroxybenzoic acid*	Phenolic acids	up	FALSE	FALSE	FALSE
MWS5235	C18H36O3	12-Hydroxyoctadecanoic acid	Lipids	FALSE	down	down	down
Zmyn005384	C18H36O3	2R-Hydroxyoctadecanoic Acid	Lipids	FALSE	FALSE	FALSE	up
pmp001176	C17H19NO4	Dihydrocaffeoyltyramine	Alkaloids	FALSE	FALSE	FALSE	down
mws0920	C15H10O7	Tricetin (5,7,3',4',5'-Pentahydroxyflavone)	Flavonoids	up	up	up	up
MWSmce549	C17H34O4	1-Monomyristin	Lipids	FALSE	FALSE	up	FALSE
mws1375	C12H21N3O6	Nicotianamine	Alkaloids	down	FALSE	FALSE	FALSE
mws0042	C15H14O7	Epigallocatechin	Flavonoids	down	down	down	FALSE

pme1373	C9H14N3O7P	2'-Deoxycytidine-5'-monophosphate	Nucleotides and derivatives	down	FALSE	FALSE	FALSE
pme1086	C10H17N3O6S	Glutathione reduced form	Amino acids and derivatives	FALSE	down	FALSE	FALSE
pmb2601	C15H16O7	7-Hydroxycoumarin-O-rhamnoside	Lignans and Coumarins	up	up	up	up
Hmqp006221	C19H34O3	13-Hydroxy-9Z,11E-octadecadienoic acid	Lipids	FALSE	FALSE	up	FALSE
pmn001689	C18H32O4	9-Hydroxy-12-oxo-15(Z)-octadecenoic acid	Lipids	FALSE	up	up	up
Lmbn007891	C18H34O4	Hydroxy ricinoleic acid	Lipids	FALSE	FALSE	up	FALSE
mws0066	C16H12O7	Isorhamnetin	Flavonoids	FALSE	FALSE	up	FALSE
Jmwn002172	C14H20O8	3,4-dihydroxyphenylethanol-β-D-glucopyranoside	Phenolic acids	down	FALSE	FALSE	down
Hmtn001120	C14H20O8	5-(2-Hydroxyethyl)-2-O-glucosylphenol	Phenolic acids	FALSE	FALSE	down	down
mws0582	C11H19N3O6S	S-(Methyl)glutathione	Amino acids and derivatives	up	up	FALSE	up
pme2074	C18H29NO4	(-)-Jasmonoyl-L-Isoleucine	Organic acids	up	up	up	up
pme3188	C9H13N2O9P	Uridine 5'-monophosphate	Nucleotides and derivatives	up	up	up	FALSE
Lmbp003825	C15H16O8	Skimmin (7-Hydroxycoumarin-7-O-glucoside)	Lignans and Coumarins	up	up	FALSE	up
Hmqp006023	C20H36O3	Ethyl 9-Hydroxy-10,12-octadecadienoic acid	Lipids	FALSE	FALSE	down	FALSE
mws0983	C20H39NO2	N-Oleoylethanolamine	Alkaloids	FALSE	FALSE	down	FALSE
HX1255	C20H22O4	(+)-trans-1,2-dihydrodehydroguaiaretic acid	Lignans and Coumarins	FALSE	down	up	FALSE
pmn001691	C18H32O5	9,12,13-Trihydroxy-10,15-octadecadienoic acid	Lipids	FALSE	up	FALSE	FALSE
mws0884	C10H12N5O6P	Cyclic 3',5'-Adenylic acid	Nucleotides and derivatives	FALSE	FALSE	down	FALSE
Lmbn003970	C18H34O5	9,12,13-TriHOME; 9(S),12(S),13(S)-Trihydroxy-10(E)-octadecenoic acid	Lipids	FALSE	FALSE	FALSE	up
pmp001284	C19H38O4	Monopalmitin	Lipids	FALSE	FALSE	FALSE	down

pmc0066	C10H13N4O7P	2'-Deoxyinosine-5'-monophosphate	Nucleotides and derivatives	up	up	up	up
pmn001421	C16H18O8	3-O-p-Coumaroylquinic acid*	Phenolic acids	up	up	FALSE	up
pmb3074	C16H18O8	5-O-p-Coumaroylquinic acid*	Phenolic acids	up	up	FALSE	up
pmb4777	C16H18O8	4-Hydroxy-7-methoxycoumarin-β-rhamnoside	Lignans and Coumarins	up	up	up	up
Zmyn000083	C6H14O12P2	D-Glucose 1,6-bisphosphate	Others	up	up	up	up
Hmcn004128	C16H20O8	P-Methoyxcinnamate glucoside	Phenolic acids	FALSE	FALSE	FALSE	up
Lmsn002548	C15H18O9	1-O-Caffeoyl-β-D-glucose*	Phenolic acids	up	FALSE	down	up
mws0264	C12H22O11	D-Trehalose	Others	up	FALSE	FALSE	up
Lmjn005592	C18H16O7	4',5-Dihydroxy-3',6,7-trimethoxyflavone	Flavonoids	down	down	down	down
mws0609	C10H12N5O7P	Guanosine 3',5'-cyclic monophosphate	Nucleotides and derivatives	up	up	up	up
HX1364	C16H18O9	isoscopoletin-glucoside	Lignans and Coumarins	up	up	FALSE	up
pmp001283	C21H38O4	1-Monolinolein	Lipids	down	FALSE	down	down
pmb0296	C21H40O4	1-Oleoyl-Sn-Glycerol	Lipids	FALSE	FALSE	down	down
mws0097	C20H22O6	Pinoresinol	Lignans and Coumarins	FALSE	FALSE	down	FALSE
pmb0964	C16H23N5O5	Isopentenyladenine-7-N-glucoside	Nucleotides and derivatives	up	FALSE	FALSE	up
Lmgp003270	C16H16O10	Scopoletin-7-O-glucuronide	Lignans and Coumarins	up	up	up	up
mws0179	C17H20O9	Chlorogenic acid methyl ester	Phenolic acids	up	up	up	up
mws0055	C20H20O7	4',5,6,7,8-Pentamethoxyflavone	Flavonoids	up	FALSE	down	FALSE
mws0232	C17H20N4O6	Riboflavin (Vitamin B2)	Others	FALSE	FALSE	FALSE	down
Lmjp003090	C17H20O10	Isofraxidin-7-O-glucoside	Lignans and Coumarins	FALSE	FALSE	down	FALSE
pmn001423	C19H30O8	Roseoside	Others	up	FALSE	FALSE	FALSE
Lmmp010562	C24H38O4	Diisooctyl Phthalate*	Phenolic acids	down	FALSE	FALSE	FALSE
mws0043	C21H22O8	5,6,7,8,3',4'-Hexamethoxyflavone	Flavonoids	up	FALSE	down	FALSE

pme3007	C9H14N2O12P2	Uridine 5'-diphosphate	Nucleotides and derivatives	up	up	up	up
pmd0152	C19H39O7P	LysoPA 16:0 (2n isomer)	Lipids	FALSE	up	up	up
pmd0153	C19H39O7P	LysoPA 16:0	Lipids	FALSE	up	up	up
mws2523	C12H23O14P	Trehalose 6-phosphate	Others	up	up	FALSE	up
Lmhp008337	C19H40NO7P	LysoPE 14:0(2n isomer)	Lipids	up	up	up	up
pmb0864	C19H40NO7P	LysoPE 14:0	Lipids	up	up	up	up
pme2117	C10H15N5O10P2	Adenosine 5'-diphosphate	Nucleotides and derivatives	up	FALSE	up	up
pmp001248	C20H30NO9+	Caffeoylcholine-4-O-glucoside	Alkaloids	FALSE	FALSE	down	FALSE
Cmbn007148	C22H22O9	1-O-Feruloyl-3-O-caffeoylglycerol	Lipids	FALSE	FALSE	up	FALSE
pme3504	C22H22O9	Formononetin-7-O-glycoside (Ononin)	Flavonoids	down	down	down	down
pmp000413	C21H20O10	Genistein-8-C-glucoside	Flavonoids	FALSE	down	FALSE	FALSE
Lmdp003509	C20H18O11	Quercetin-3-O-xyloside*	Flavonoids	up	FALSE	FALSE	up
mws2186	C20H18O11	Quercetin-3-O-α-L-arabinofuranoside*	Flavonoids	up	FALSE	FALSE	up
Lmhp008273	C20H40NO7P	LysoPE 15:1(2n isomer)	Lipids	up	up	up	up
Lmhp008440	C20H40NO7P	LysoPE 15:1	Lipids	FALSE	up	up	FALSE
Lmhp009187	C20H42NO7P	LysoPE 15:0	Lipids	up	up	up	up
Lmhp008885	C20H42NO7P	LysoPE 15:0(2n isomer)	Lipids	up	up	up	up
Hmcp002316	C21H20O11	Isorhamnetin-3-O-arabinoside	Flavonoids	up	FALSE	FALSE	up
Xmyp005654	C21H20O11	Kaempferol-4'-O-glucoside*	Flavonoids	FALSE	up	up	FALSE
Lmmn004625	C21H22O11	Dihydrokaempferol-7-O-glucoside*	Flavonoids	up	up	FALSE	up
MWS20145	C21H22O11	Eriodictyol-7-O-glucoside*	Flavonoids	down	FALSE	FALSE	FALSE
HJN086	C21H22O11	Eriodictyol-3'-O-glucoside*	Flavonoids	up	up	FALSE	up
Jmgn004021	C21H22O11	6-C-Glucosyl-2-Hydroxynaringenin	Flavonoids	down	FALSE	down	down
Lmhp009034	C21H42NO7P	LysoPE 16:1	Lipids	up	up	up	up

Lmhp008763	C21H42NO7P	LysoPE 16:1(2n isomer)	Lipids	up	up	up	up
Hmpn005101	C21H24O11	Sieboldin	Flavonoids	down	FALSE	FALSE	FALSE
Zmhn001257	C21H24O11	Catechin-5-O-glucoside	Flavonoids	up	FALSE	up	FALSE
pmd0160	C21H44NO7P	LysoPE 16:0(2n isomer)	Lipids	up	up	up	up
pmb0876	C21H44NO7P	LysoPE 16:0	Lipids	up	up	up	up
Lmhn004756	C23H20O10	Cinnamoylferuloyltartaric acid	Phenolic acids	down	down	FALSE	down
Zmjn014062	C30H48O3	(23S)-3β-hydroxydammara-21-oic acid 21,23-lactone	Terpenoids	up	up	FALSE	up
pmb2654	C19H27NO12	Anthranilate-1-O-Sophoroside	Phenolic acids	up	up	FALSE	up
Lmhn002321	C21H18O12	Vnilloylcaffeoyltartaric acid	Phenolic acids	FALSE	FALSE	FALSE	up
pmb3012	C22H22O11	Chrysoeriol-7-O-glucoside	Flavonoids	down	FALSE	FALSE	down
pmp001309	C21H20O12	6-Hydroxykaempferol-7-O-glucoside	Flavonoids	up	FALSE	FALSE	up
Hmcn001884	C21H20O12	6-Hydroxyluteolin 5-glucoside	Flavonoids	FALSE	FALSE	up	FALSE
MWSHY0046	C21H20O12	Quercetin-3-O-glucoside*	Flavonoids	FALSE	FALSE	up	FALSE
Lmdp003286	C21H20O12	Isohyperoside*	Flavonoids	up	up	up	up
MWSHY0113	C21H20O12	Quercetin-3-O-galactoside*	Flavonoids	up	up	up	up
mws1329	C21H20O12	Quercetin-7-O-glucoside	Flavonoids	up	up	up	up
Smgp004575	C21H20O12	Quercetin-5-O-β-D-glucoside*	Flavonoids	FALSE	FALSE	up	up
Lmhp009769	C22H44NO7P	LysoPE 17:1	Lipids	up	up	up	up
Xmsn002700	C21H22O12	Taxifolin-3'-O-glucoside	Flavonoids	up	FALSE	FALSE	up
pmd0130	C22H46NO7P	LysoPC 14:0	Lipids	FALSE	up	up	FALSE
Qmgp102003	C29H41NO4	O-Acetyljervine	Alkaloids	FALSE	up	up	FALSE
Lmhp008233	C23H40NO7P	LysoPE 18:4	Lipids	up	up	up	FALSE
Lmhp008801	C23H42NO7P	LysoPE 18:3	Lipids	up	up	up	up
Lmhp008589	C23H42NO7P	LysoPE 18:3(2n isomer)	Lipids	up	up	up	up
pmb0881	C23H44NO7P	LysoPE 18:2	Lipids	up	up	up	up
pmb0874	C23H44NO7P	LysoPE 18:2(2n isomer)	Lipids	up	up	up	up

Lmmp003783	C21H18O13	Quercetin-3-O-glucuronide	Flavonoids	up	up	up	up
Lmqp003647	C21H18O13	Herbacetin-3-O-glucuronide	Flavonoids	up	FALSE	up	FALSE
Lmcp003788	C22H22O12	Isotamarixin	Flavonoids	FALSE	FALSE	up	FALSE
Hmcp002207	C22H22O12	Isorhamnetin-7-O-glucoside*	Flavonoids	FALSE	FALSE	up	FALSE
mws0289	C23H46NO7P	LysoPE 18:1	Lipids	up	up	up	up
pmb2260	C23H46NO7P	LysoPC 15:1	Lipids	FALSE	up	up	up
pmb0856	C23H46NO7P	LysoPE 18:1(2n isomer)	Lipids	up	up	up	up
pmb0880	C23H48NO7P	LysoPE 18:0(2n isomer)	Lipids	up	up	up	up
Lmhp009129	C23H48NO7P	LysoPC 15:0(2n isomer)	Lipids	FALSE	up	up	FALSE
pmb0883	C23H48NO7P	LysoPE 18:0	Lipids	up	up	up	up
pmb2319	C23H48NO7P	LysoPC 15:0	Lipids	FALSE	up	up	up
Lmsn011830	C30H46O5	2α,19α-Dihydroxy-3-oxours-12-en-28-oic acid	Terpenoids	FALSE	FALSE	up	FALSE
Lmmn003398	C23H22O12	Kaempferol-3-O-(6"-acetyl)glucoside	Flavonoids	FALSE	FALSE	FALSE	down
pmb0863	C24H46NO7P	LysoPC 16:2(2n isomer)	Lipids	up	up	up	up
pmp001270	C24H48NO7P	LysoPC 16:1	Lipids	FALSE	up	up	FALSE
Lmhp008833	C24H48NO7P	LysoPC 16:1(2n isomer)	Lipids	up	up	up	up
pmd0132	C24H50NO7P	LysoPC 16:0(2n isomer)	Lipids	up	up	up	up
pmb0855	C24H50NO7P	LysoPC 16:0	Lipids	up	up	up	up
mad2394	C24H22O12	Sinapoyl-p-coumaroyltartaric acid	Phenolic acids	up	FALSE	FALSE	up
Lmhp009802	C25H46NO7P	LysoPE 20:3(2n isomer)	Lipids	up	up	up	up
Lmhp010040	C25H46NO7P	LysoPE 20:3	Lipids	up	up	up	up
mws1589	C18H32O16	D-Panose	Others	up	FALSE	FALSE	up
MWS0442	C18H32O16	Maltotriose	Others	FALSE	FALSE	down	FALSE
Lmhp010514	C25H48NO7P	LysoPE 20:2(2n isomer)	Lipids	up	up	up	up
Lmhp008718	C25H48NO7P	LysoPC 17:2	Lipids	up	up	up	up
Lmhp010757	C25H48NO7P	LysoPE 20:2	Lipids	up	up	up	up

Hmln002199	C23H22O13	Quercetin-3-O-(6''-O-acetyl)galactoside	Flavonoids	up	FALSE	FALSE	up
Lmhp009590	C25H50NO7P	LysoPC 17:1	Lipids	FALSE	up	up	FALSE
pmb2406	C25H52NO7P	LysoPC 17:0	Lipids	FALSE	up	FALSE	FALSE
Lmgp004518	C26H24O11	CaffeoylferuloylQuinic acid	Phenolic acids	up	up	up	up
Lmhp009773	C27H46O9	1-α-Linolenoyl-glycerol-3-O-glucoside	Lipids	FALSE	FALSE	down	down
Hmqp006235	C26H46NO7P	LysoPC 18:4	Lipids	up	up	up	up
MWSmce328	C25H24O12	Isochlorogenic acid C	Phenolic acids	up	up	FALSE	up
Li512115	C25H24O12	Isochlorogenic acid B	Phenolic acids	up	FALSE	FALSE	up
Wmzn002116	C25H24O12	3,5-Dicaffeoylquinic acid	Phenolic acids	FALSE	FALSE	FALSE	up
mws1584	C25H24O12	1,3-O-Dicaffeoylquinic Acid	Phenolic acids	FALSE	FALSE	FALSE	up
Lmhp010573	C27H48O9	1-Linoleoylglycerol-3-O-glucoside	Lipids	FALSE	FALSE	FALSE	down
pmb0854	C26H48NO7P	LysoPC 18:3	Lipids	up	up	up	up
pmb0865	C26H48NO7P	LysoPC 18:3(2n isomer)	Lipids	up	up	up	up
pmp001273	C26H50NO7P	LysoPC 18:2	Lipids	up	up	up	up
pmp001251	C26H50NO7P	LysoPC 18:2(2n isomer)	Lipids	up	up	up	up
pmn001644	C23H20O14	Quercetin-3-O-(2''-O-acetyl)glucuronide	Flavonoids	down	FALSE	FALSE	FALSE
pmp000682	C26H32O11	Matairesinol-4'-O-glucoside	Lignans and Coumarins	FALSE	up	up	FALSE
pmp001281	C26H52NO7P	LysoPC 18:1	Lipids	FALSE	up	up	up
Lmhp010190	C26H52NO7P	LysoPC 18:1(2n isomer)	Lipids	up	up	up	up
pmn001710	C24H26O13	Rosmarinic acid-3'-O-glucoside	Phenolic acids	FALSE	FALSE	FALSE	down
Zmgp005070	C24H26O13	Centaurein	Flavonoids	FALSE	FALSE	down	FALSE
pmd0136	C26H54NO7P	LysoPC 18:0(2n isomer)	Lipids	up	up	up	up
mws0126	C26H54NO7P	LysoPC 18:0	Lipids	up	up	up	up
Lmjp003731	C26H26O12	3,4-O-Dicaffeoylquinic Acid Methyl Ester	Phenolic acids	up	up	up	up
Zmfn000481	C14H22N2O16P2	Uridine-5'-Diphosphate-D-Xylose	Nucleotides and derivatives	up	FALSE	up	up

Lmdn001925	C26H34O12	Olivil-4'-O-glucoside	Lignans and Coumarins	down	FALSE	FALSE	down
pmc0960	C28H50NO7P	LysoPC 20:4	Lipids	FALSE	FALSE	down	down
Lmhp009890	C28H52NO7P	LysoPC 20:3	Lipids	FALSE	up	FALSE	FALSE
pmd0146	C28H54NO7P	LysoPC 20:2(2n isomer)	Lipids	FALSE	up	up	up
pmd0147	C28H54NO7P	LysoPC 20:2	Lipids	FALSE	up	up	up
pma0702	C29H39N3O8	N1,N8-Bis(sinapoyl)spermidine	Alkaloids	down	FALSE	down	down
Lmhn003802	C26H26O14	Sinapoylsinapoyltartaric acid	Phenolic acids	FALSE	FALSE	down	FALSE
Zmhp005139	C25H24O15	Tamarixetin-3-O-(6"-malonyl)glucoside*	Flavonoids	up	FALSE	FALSE	up
pmb2922	C15H24N2O17P2	Uridine 5'-diphospho-D-glucose	Nucleotides and derivatives	up	FALSE	FALSE	FALSE
Hmcp001769	C26H28O15	Quercetin-3-O-rhamnosyl(1→2)arabinoside	Flavonoids	FALSE	up	up	up
Rfmb056	C29H36O13	Medioresinol-4'-O-(6'''-acetyl)glucoside	Lignans and Coumarins	FALSE	FALSE	down	FALSE
Lmsn002815	C27H30O15	Kaempferol-3-O-rutinoside	Flavonoids	down	down	FALSE	down
Lmjp002596	C26H28O16	Quercetin-3-O-sambubioside	Flavonoids	up	FALSE	FALSE	up
Lmmp000897	C30H26O14	Gallocatechin-(4α→8)-gallocatechin	Flavonoids	up	up	FALSE	up
Lmsp004166	C27H30O16	Quercetin-3-O-glucoside-7-O-rhamnoside	Flavonoids	up	FALSE	FALSE	up
pmb0665	C27H30O16	Orientin-7-O-glucoside	Flavonoids	up	FALSE	FALSE	up
mws4134	C20H32N6O12S2	Oxiglutatione	Amino acids and derivatives	FALSE	FALSE	up	FALSE
Cmxp003531	C27H32O16	Okanin-4'-O-gentiobioside	Flavonoids	down	FALSE	down	down
XLn05122	C29H28O15	1,4-O-dicaffeoyl-3-O-succinoyl-quinic acid*	Phenolic acids	up	up	up	up
XLn05515	C29H28O15	1,5-O-dicaffeoyl-3-O-succinoyl-quinic acid*	Phenolic acids	up	up	up	up
Lmhp003217	C28H32O16	2'-Hydoxy,5-methoxyGenistein-O-rhamnosyl-glucoside*	Flavonoids	FALSE	FALSE	FALSE	up
Lmmp002463	C28H32O16	Sexangularetin-3-O-glucoside-7-O-rhamnoside*	Flavonoids	FALSE	FALSE	FALSE	up
MWSHY0064	C28H32O16	Isorhamnetin-3-O-neohesperidoside*	Flavonoids	FALSE	FALSE	FALSE	up
Lmsp004721	C28H32O16	Tamarixetin-3-O-glucoside-7-O-rhamnoside*	Flavonoids	FALSE	FALSE	FALSE	up

Lmnp002584	C28H32O16	Hispidulin-8-C-(2"-O-glucosyl)glucoside*	Flavonoids	FALSE	down	FALSE	FALSE
Zmdp003457	C27H30O17	Myricetin-3-O-galactoside-3'-O-rhamnoside*	Flavonoids	FALSE	FALSE	FALSE	up
MWSHY0162	C27H30O17	Quercetin-3-O-sophoroside (Baimaside)*	Flavonoids	up	FALSE	FALSE	up
Lmsp003729	C27H30O17	Myricetin-3-O-rutinoside*	Flavonoids	FALSE	FALSE	FALSE	up
Zmcp002666	C27H30O17	Quercetin-3,7-Di-O-glucoside	Flavonoids	up	FALSE	FALSE	up
HJN038	C28H24O17	Myricetin-3-O-(6"-galloyl)glucoside	Flavonoids	up	FALSE	FALSE	up
Lmmp002560	C27H28O18	Quercetin-3-O-(2"-O-glucosyl)glucuronide	Flavonoids	up	FALSE	up	FALSE
Hmcp001578	C28H32O17	Isorhamnetin-3,7-O-diglucoside	Flavonoids	up	up	up	up
XLn05373	C31H32O16	1,5-O-dicaffeoyl-3-O-dimethylmalyl-quinic acid	Phenolic acids	FALSE	FALSE	up	FALSE
mws4163	C24H42O21	Nystose	Others	up	FALSE	FALSE	FALSE
mws1593	C24H42O21	D-Maltotetraose	Others	up	FALSE	FALSE	up
Lmqn000213	C24H42O21	Stachyose	Others	up	up	FALSE	up
Lmxp010913	C35H66NO9P	1-(9Z-Octadecenoyl)-2-(9-oxo-nonanoyl)-sn-glycero-3-phosphocholine	Lipids	down	FALSE	FALSE	FALSE
Lmyn005812	C33H56O14	Gingerglycolipid A	Lipids	FALSE	up	FALSE	FALSE
Lmhp008744	C33H56O14	1-α-Linolenoyl-glycerol-2,3-di-O-glucoside	Lipids	FALSE	up	FALSE	FALSE
pmp001276	C33H56O14	1-Linolenoyl-rac-glycerol-diglucoside	Lipids	FALSE	FALSE	down	down
HJN102	C34H30O15	3,4,5-Tricaffeoylquinic acid	Phenolic acids	up	up	FALSE	up
Lmgn002250	C31H34O17	Dicaffeoylquinic acid-O-glucoside	Phenolic acids	down	down	down	down
XLn04217	C31H34O17	1,5-O-dicaffeoyl-3-O-glucoside-quinic acid	Phenolic acids	up	up	FALSE	up
Lmyn006011	C33H58O14	Gingerglycolipid B	Lipids	FALSE	up	FALSE	FALSE
Lmmn004145	C38H60O10	3-O-(2-O-Acetyl-glucosyl)oleanolic acid	Terpenoids	FALSE	up	FALSE	FALSE
Lmgn002253	C31H36O18	Syringoylcaffeoylquinic acid-D-glucose	Phenolic acids	up	FALSE	FALSE	FALSE
pmb0709	C30H32O20	Quercetin-7-O-(6"-malonyl)glucosyl-5-O-glucoside	Flavonoids	FALSE	FALSE	down	FALSE
pmb0706	C30H32O20	Quercetin-3-O-(6"-O-malonyl)glucosyl-5-O-glucoside	Flavonoids	FALSE	FALSE	FALSE	up
XLn05670	C33H32O18	3,5-Di-O-caffeoyl-1-O-(2,4-disuccinoyl)-quinic acid	Phenolic acids	up	up	up	up
Lmgn004359	C33H38O18	SinapoylcaffeoylQuinic acid O-glucose	Phenolic acids	up	FALSE	FALSE	FALSE

Hmcp001658	C31H34O20	Isorhamnetin-3-O-(6"-malonyl)glucoside-7-O-glucoside	Flavonoids	up	FALSE	FALSE	up
pme2651	C21H28N7O17P3	NADP (Nicotinamide adenine dinucleotide phosphate)	Nucleotides and derivatives	up	FALSE	up	up
HJAP127	C33H40O20	Quercetin-3-O-(2"-O-Rhamnosyl)rutinoside	Flavonoids	up	FALSE	FALSE	up
Zmhp002640	C33H40O21	6-Hydroxykaempferol-3-O-Rutinoside-6-O-glucoside*	Flavonoids	up	FALSE	FALSE	FALSE
XLn05839	C38H34O19	3,5-Di-O-caffeoyl-1-O-(2-O-caffeoylmaloyl)-quinic acid	Phenolic acids	FALSE	up	FALSE	FALSE
XLn04478	C35H38O21	3,5-Di-O-caffeoyl-1-O-(2-O-glucosylmaloyl)-quinic acid*	Phenolic acids	up	up	FALSE	up
XLn04250	C35H38O21	3,5-Di-O-caffeoyl-1-O-(4-O-glucosylmaloyl)-quinic acid*	Phenolic acids	up	up	FALSE	up
Lmdn000248	C30H52O26	Verbascose	Others	up	up	FALSE	up
XLn05736	C42H38O23	3,4,5-Tri-O-caffeoyl-1-O-(2,4-dimaloyl)-quinic acid	Phenolic acids	FALSE	up	FALSE	FALSE

Table S3. Variation of differential metabolite expression content in group T1 vs T3.

Formula	Compounds	Class	T1	T3	VIP	p_value	FDR	Fold_Change	Type
C5H11NO2	5-Aminovaleric acid	Organic acids	6.76E+04	2.10E+04	1.12	0.18	0.37	0.31	down
C5H10O3	β-Hydroxyisovaleric acid	Organic acids	5.94E+05	1.54E+06	1.35	0.11	0.33	2.59	up
C8H8O	(S)-2-Phenyloxirane	Others	1.44E+07	6.66E+06	1.05	0.17	0.36	0.46	down
C3H7NO2S	L-Cysteine	Amino acids and derivatives	1.05E+05	2.37E+05	1.03	0.29	0.50	2.26	up
C8H10O	1-Phenylethanol	Phenolic acids	2.03E+05	4.99E+05	1.32	0.07	0.29	2.45	up
C4H9N3O2	3-Guanidinopropionic acid	Organic acids	8.17E+04	3.68E+04	1.52	0.00	0.04	0.45	down
C4H6O5	3-Dehydro-L-Threonic Acid*	Others	1.22E+07	2.80E+07	1.24	0.13	0.33	2.29	up
C4H6O5	D-Malic acid*	Organic acids	1.11E+07	2.58E+07	1.24	0.12	0.33	2.32	up
C8H8O2	Benzoic acid methyl ester	Phenolic acids	5.54E+04	9.00E+00	1.53	0.00	0.13	0.00	down
C8H11NO	L-Tyramine	Alkaloids	1.60E+07	7.34E+06	1.05	0.17	0.36	0.46	down
C5H4N4O2	Xanthine	Nucleotides and derivatives	9.36E+04	2.30E+05	1.24	0.10	0.32	2.45	up
C8H8O3	Vanillin	Phenolic acids	6.95E+04	1.39E+05	1.40	0.01	0.18	2.00	up

C10H12N2	Tryptamine	Alkaloids	1.95E+05	5.66E+05	1.31	0.11	0.33	2.89	up
C10H12O2	Ethyl phenylacetate	Phenolic acids	3.01E+05	8.29E+05	1.36	0.09	0.32	2.76	up
C8H8O4	Protocatechuic Acid Methyl Ester	Phenolic acids	3.09E+05	1.04E+05	1.26	0.14	0.34	0.34	down
C7H14N2O3	N-α-Acetyl-L-ornithine	Amino acids and derivatives	2.99E+06	1.13E+06	1.51	0.00	0.07	0.38	down
C10H9NO2	1-Methoxyindole-3-carbaldehyde	Alkaloids	3.29E+04	9.00E+00	1.53	0.02	0.24	0.00	down
C11H12O2	Ethyl cinnamate	Phenolic acids	1.67E+04	3.72E+04	1.37	0.03	0.28	2.23	up
C10H10O3	4-Methoxycinnamic acid	Phenolic acids	8.24E+03	3.59E+03	1.00	0.13	0.33	0.44	down
C9H8O4	Caffeic acid	Phenolic acids	3.38E+06	1.35E+06	1.13	0.23	0.44	0.40	down
C6H12O6	D-Fructose*	Others	8.39E+06	3.44E+06	1.32	0.06	0.29	0.41	down
C6H12O6	D-Glucose*	Others	7.04E+06	2.92E+06	1.31	0.06	0.29	0.41	down
C6H12O6	Inositol*	Others	8.26E+06	3.42E+06	1.32	0.06	0.29	0.41	down
C7H12N2O4	N-Acetyl-L-Glutamine	Amino acids and derivatives	3.80E+05	8.18E+04	1.45	0.07	0.29	0.22	down
C8H16N2O3	L-Glycyl-L-isoleucine	Amino acids and derivatives	2.41E+05	5.34E+05	1.20	0.06	0.29	2.22	up
C7H11NO5	N-Acetyl-L-glutamic acid	Amino acids and derivatives	1.36E+06	6.52E+05	1.42	0.07	0.29	0.48	down
C9H19NO4	Pantothenol	Others	2.21E+04	3.75E+03	1.14	0.11	0.33	0.17	down
C11H12O4	Ethyl caffeate	Phenolic acids	6.65E+04	2.59E+04	1.31	0.02	0.26	0.39	down
C12H18O3	Jasmonic acid	Organic acids	1.25E+05	5.72E+04	1.17	0.08	0.30	0.46	down
C12H24O3	12-Hydroxydodecanoic acid	Lipids	1.09E+04	9.00E+00	1.52	0.10	0.32	0.00	down
C12H18O4	3-(3,4,5-Trimethoxyphenyl)propan-1-ol	Phenolic acids	1.41E+06	5.81E+04	1.43	0.08	0.31	0.04	down
C10H20O6	Butyl Beta-D-Fructopyranoside	Others	2.69E+05	1.30E+05	1.22	0.10	0.32	0.48	down
C10H14N2O5	Thymidine	Nucleotides and derivatives	9.00E+00	3.36E+04	1.53	0.07	0.29	3736.33	up
C16H30O2	(7Z)-Hexadecenoic acid	Lipids	1.97E+04	9.81E+03	1.41	0.04	0.28	0.50	down
C15H10O5	Apigenin; 4',5,7-Trihydroxyflavone	Flavonoids	7.88E+04	1.76E+05	1.27	0.11	0.32	2.23	up
C15H10O5	3,5,7-Trihydroxyflavone	Flavonoids	1.23E+05	2.97E+05	1.46	0.03	0.27	2.42	up
C18H28O2	9,12-Octadecadien-6-Ynoic Acid	Lipids	9.00E+00	4.26E+05	1.53	0.02	0.26	47386.30	up
C17H16O4	Phenethyl caffeate	Phenolic acids	1.03E+05	2.24E+05	1.19	0.15	0.34	2.18	up

C15H10O6	2'-Hydroxygenistein*	Flavonoids	3.00E+04	7.40E+04	1.41	0.07	0.29	2.47	up
C15H10O6	Luteolin (5,7,3',4'-Tetrahydroxyflavone)*	Flavonoids	3.00E+04	7.40E+04	1.41	0.07	0.29	2.47	up
C17H23NO3	L-Hyoscyamine	Alkaloids	1.09E+04	2.24E+04	1.34	0.02	0.23	2.05	up
C13H12O8	2-O-Caffeoylmalic acid	Phenolic acids	9.00E+00	7.24E+04	1.53	0.14	0.34	8044.37	up
C18H32O3	9-Oxo-12Z-Octadecenoic acid	Lipids	3.31E+05	1.47E+05	1.15	0.18	0.37	0.44	down
C18H34O3	Ricinoleic acid	Lipids	5.96E+04	2.02E+05	1.19	0.23	0.44	3.39	up
C20H42O	1-Eicosanol	Lipids	6.16E+04	1.96E+05	1.18	0.23	0.44	3.18	up
C13H16O8	4-O-Glucosyl-4-hydroxybenzoic acid*	Phenolic acids	9.65E+05	2.66E+05	1.38	0.04	0.28	0.28	down
C17H34O4	1-Monomyristin	Lipids	3.54E+05	7.32E+05	1.46	0.04	0.28	2.07	up
C9H14N3O7P	2'-Deoxycytidine-5'-monophosphate	Nucleotides and derivatives	9.00E+00	1.08E+04	1.53	0.01	0.15	1204.77	up
C16H12O7	Isorhamnetin	Flavonoids	7.14E+05	1.52E+06	1.32	0.05	0.29	2.12	up
C11H19N3O6S	S-(Methyl)glutathione	Amino acids and derivatives	1.14E+05	4.96E+04	1.22	0.02	0.25	0.44	down
C15H16O8	Skimmin (7-Hydroxycoumarin-7-O-glucoside)	Lignans and Coumarins	2.06E+06	8.37E+05	1.40	0.09	0.31	0.41	down
C20H36O3	Ethyl 9-Hydroxy-10,12-octadecadienoic acid	Lipids	9.59E+04	2.43E+04	1.25	0.12	0.33	0.25	down
C20H39NO2	N-Oleoylethanolamine	Alkaloids	6.21E+05	1.60E+05	1.26	0.12	0.33	0.26	down
C20H22O4	(+)-trans-1,2-dihydrodehydroguaiaretic acid	Lignans and Coumarins	7.55E+03	1.77E+04	1.18	0.14	0.33	2.35	up
C14H18O9	1-O-Vanilloyl-D-Glucose*	Phenolic acids	1.30E+05	6.16E+04	1.45	0.00	0.13	0.47	down
C14H18O9	5-Glucosyloxy-2-Hydroxybenzoic acid methyl ester*	Phenolic acids	1.80E+05	8.07E+04	1.32	0.05	0.29	0.45	down
C10H13N4O7P	2'-Deoxyinosine-5'-monophosphate	Nucleotides and derivatives	2.18E+06	7.00E+05	1.10	0.03	0.27	0.32	down
C16H18O8	5-O-p-Coumaroylquinic acid*	Phenolic acids	2.67E+07	1.31E+07	1.30	0.08	0.30	0.49	down
C15H16O9	Daphnin*	Lignans and Coumarins	1.80E+06	8.61E+05	1.52	0.00	0.13	0.48	down
C15H16O9	Esculetin-7-O-glucoside*	Lignans and Coumarins	2.02E+06	9.82E+05	1.52	0.00	0.13	0.49	down
C15H18O9	1-O-Caffeoyl-β-D-glucose*	Phenolic acids	8.72E+05	1.49E+05	1.13	0.04	0.28	0.17	down
C12H22O11	D-Trehalose	Others	1.39E+06	5.27E+05	1.16	0.11	0.33	0.38	down
C18H16O7	4',5-Dihydroxy-3',6,7-trimethoxyflavone	Flavonoids	7.91E+03	3.72E+02	1.28	0.10	0.32	0.05	down
C21H38O4	1-Monolinolein	Lipids	3.24E+04	9.00E+00	1.53	0.05	0.29	0.00	down

C20H22O6	Pinoresinol	Lignans and Coumarins	7.30E+04	2.66E+04	1.37	0.09	0.32	0.36	down
C16H23N5O5	Isopentenyladenine-7-N-glucoside	Nucleotides and derivatives	4.26E+05	1.31E+05	1.46	0.05	0.29	0.31	down
C20H20O7	4',5,6,7,8-Pentamethoxyflavone	Flavonoids	2.76E+05	1.03E+04	1.36	0.30	0.51	0.04	down
C19H30O8	Roseoside	Others	3.83E+05	1.49E+05	1.49	0.00	0.13	0.39	down
C21H22O8	5,6,7,8,3',4'-Hexamethoxyflavone	Flavonoids	2.02E+05	1.33E+04	1.41	0.19	0.38	0.07	down
C20H40NO7P	LysoPE 15:1(2n isomer)	Lipids	8.38E+03	1.69E+04	1.10	0.21	0.41	2.01	up
C20H40NO7P	LysoPE 15:1	Lipids	1.15E+04	3.19E+04	1.33	0.10	0.32	2.76	up
C20H42NO7P	LysoPE 15:0	Lipids	9.06E+04	2.05E+05	1.24	0.09	0.31	2.26	up
C20H42NO7P	LysoPE 15:0(2n isomer)	Lipids	8.17E+04	1.87E+05	1.41	0.03	0.27	2.28	up
C21H22O11	Dihydrokaempferol-7-O-glucoside*	Flavonoids	1.47E+06	2.46E+05	1.37	0.17	0.36	0.17	down
C21H22O11	Eriodictyol-3'-O-glucoside*	Flavonoids	1.53E+06	2.43E+05	1.36	0.19	0.38	0.16	down
C23H20O10	Cinnamoylferuloyltartaric acid	Phenolic acids	9.00E+00	7.48E+04	1.53	0.03	0.26	8306.15	up
C30H48O3	(23S)-3β-hydroxydammara-21-oic acid 21,23-lactone	Terpenoids	5.55E+03	9.00E+00	1.53	0.01	0.17	0.00	down
C19H27NO12	Anthranilate-1-O-Sophoroside	Phenolic acids	1.26E+06	3.32E+05	1.43	0.00	0.14	0.26	down
C21H18O12	Vnilloylcaffeoyltartaric acid	Phenolic acids	6.32E+04	3.15E+04	1.20	0.15	0.34	0.50	down
C21H22O12	Taxifolin-3'-O-glucoside	Flavonoids	4.15E+05	1.66E+05	1.15	0.10	0.32	0.40	down
C22H22O12	Isotamarixin	Flavonoids	2.17E+05	6.62E+05	1.21	0.15	0.34	3.05	up
C23H46NO7P	LysoPE 18:1	Lipids	7.26E+05	1.51E+06	1.37	0.06	0.29	2.08	up
C23H46NO7P	LysoPC 15:1	Lipids	5.26E+04	1.11E+05	1.52	0.00	0.07	2.12	up
C23H46NO7P	LysoPE 18:1(2n isomer)	Lipids	4.38E+05	8.83E+05	1.33	0.07	0.29	2.02	up
C23H48NO7P	LysoPE 18:0(2n isomer)	Lipids	9.00E+03	2.03E+04	1.30	0.03	0.27	2.26	up
C23H48NO7P	LysoPC 15:0	Lipids	3.25E+05	6.91E+05	1.43	0.06	0.29	2.13	up
C24H48NO7P	LysoPC 16:1	Lipids	1.68E+05	5.81E+05	1.24	0.06	0.29	3.46	up
C25H46NO7P	LysoPE 20:3(2n isomer)	Lipids	1.07E+04	2.54E+04	1.44	0.04	0.28	2.38	up
C25H46NO7P	LysoPE 20:3	Lipids	1.28E+04	3.00E+04	1.31	0.10	0.32	2.35	up
C18H32O16	D-Panose	Others	5.86E+05	5.89E+04	1.39	0.08	0.31	0.10	down

C18H32O16	Maltotriose	Others	1.11E+06	2.72E+05	1.29	0.10	0.32	0.25	down
C25H48NO7P	LysoPE 20:2(2n isomer)	Lipids	1.71E+04	3.84E+04	1.24	0.08	0.30	2.25	up
C25H48NO7P	LysoPC 17:2	Lipids	5.62E+04	1.15E+05	1.35	0.04	0.28	2.05	up
C25H48NO7P	LysoPE 20:2	Lipids	1.84E+04	4.14E+04	1.20	0.11	0.33	2.25	up
C25H50NO7P	LysoPC 17:1	Lipids	1.30E+05	2.77E+05	1.45	0.01	0.17	2.12	up
C25H24O12	Isochlorogenic acid C	Phenolic acids	9.52E+05	4.29E+05	1.44	0.01	0.18	0.45	down
C25H24O12	3,5-Dicaffeoylquinic acid	Phenolic acids	1.30E+07	6.03E+06	1.49	0.01	0.14	0.46	down
C25H24O12	1,3-O-Dicaffeoylquinic Acid	Phenolic acids	1.22E+07	5.12E+06	1.51	0.00	0.13	0.42	down
C23H20O14	Quercetin-3-O-(2''-O-acetyl)glucuronide	Flavonoids	3.34E+04	8.63E+04	1.15	0.12	0.33	2.58	up
C26H52NO7P	LysoPC 18:1	Lipids	9.00E+00	4.33E+06	1.53	0.01	0.14	########	up
C24H26O13	Rosmarinic acid-3'-O-glucoside	Phenolic acids	1.14E+05	2.72E+05	1.35	0.06	0.29	2.38	up
C24H26O13	Centaurein	Flavonoids	1.00E+05	4.36E+04	1.51	0.00	0.13	0.44	down
C26H54NO7P	LysoPC 18:0(2n isomer)	Lipids	3.44E+05	6.89E+05	1.28	0.14	0.33	2.00	up
C26H54NO7P	LysoPC 18:0	Lipids	3.44E+05	6.89E+05	1.28	0.14	0.33	2.00	up
C24H22O14	Luteolin-7-O-(6''-malonyl)glucoside	Flavonoids	1.09E+05	4.26E+04	1.39	0.04	0.28	0.39	down
C28H50NO7P	LysoPC 20:4	Lipids	1.03E+04	9.00E+00	1.49	0.20	0.40	0.00	down
C28H54NO7P	LysoPC 20:2(2n isomer)	Lipids	6.28E+04	1.68E+05	1.41	0.06	0.29	2.68	up
C28H54NO7P	LysoPC 20:2	Lipids	6.64E+04	1.66E+05	1.48	0.04	0.28	2.50	up
C24H22O15	Quercetin-7-O-(6''-malonyl)glucoside	Flavonoids	1.06E+07	3.44E+06	1.44	0.01	0.15	0.33	down
C26H26O14	Sinapoylsinapoyltartaric acid	Phenolic acids	1.13E+04	9.00E+00	1.53	0.01	0.17	0.00	down
C25H24O15	Tamarixetin-3-O-(6''-malonyl)glucoside*	Flavonoids	3.48E+06	9.16E+05	1.52	0.02	0.23	0.26	down
C29H36O13	Medioresinol-4'-O-(6'''-acetyl)glucoside	Lignans and Coumarins	9.88E+04	1.58E+04	1.11	0.20	0.39	0.16	down
C27H30O15	Kaempferol-3-O-rutinoside	Flavonoids	6.37E+05	1.32E+06	1.30	0.09	0.31	2.07	up
C26H28O16	Quercetin-3-O-sambubioside	Flavonoids	1.38E+06	6.77E+05	1.46	0.02	0.26	0.49	down
C29H34O17	Syringetin-3-O-rutinoside	Flavonoids	2.71E+06	1.19E+06	1.39	0.06	0.29	0.44	down
C24H42O21	D-Maltotetraose	Others	1.62E+05	1.69E+04	1.36	0.00	0.07	0.10	down

C24H42O21	Stachyose	Others	4.79E+05	7.21E+04	1.45	0.00	0.14	0.15	down
C35H66NO9P	1-(9Z-Octadecenoyl)-2-(9-oxo-nonanoyl)-sn-glycero-3-phosphocholine	Lipids	9.63E+03	3.60E+04	1.10	0.10	0.32	3.74	up
C34H30O15	3,4,5-Tricaffeoylquinic acid	Phenolic acids	8.12E+06	1.22E+06	1.34	0.15	0.34	0.15	down
C31H34O17	1,5-O-dicaffeoyl-3-O-glucoside-quinic acid	Phenolic acids	9.56E+06	1.43E+06	1.33	0.16	0.35	0.15	down
C31H36O18	Syringoylcaffeoylquinic acid-D-glucose	Phenolic acids	2.52E+06	9.43E+05	1.48	0.01	0.18	0.37	down
C30H32O20	Quercetin-7-O-(6''-malonyl)glucosyl-5-O-glucoside	Flavonoids	4.79E+04	4.13E+03	1.21	0.00	0.13	0.09	down
C30H32O20	Quercetin-3-O-(6''-O-malonyl)glucosyl-5-O-glucoside	Flavonoids	6.29E+04	2.92E+04	1.23	0.12	0.33	0.46	down
C33H40O21	6-Hydroxykaempferol-3-O-Rutinoside-6-O-glucoside*	Flavonoids	3.41E+06	1.52E+06	1.48	0.00	0.13	0.44	down
C35H38O21	3,5-Di-O-caffeoyl-1-O-(2-O-glucosylmaloyl)-quinic acid*	Phenolic acids	1.97E+06	5.03E+05	1.23	0.03	0.26	0.25	down
C35H38O21	3,5-Di-O-caffeoyl-1-O-(4-O-glucosylmaloyl)-quinic acid*	Phenolic acids	1.97E+06	5.03E+05	1.23	0.03	0.26	0.25	down
C30H52O26	Verbascose	Others	8.78E+04	8.22E+03	1.36	0.03	0.26	0.09	down

Table S4. Variation of differential metabolite expression content in group T2 vs T3.

Formula	Compounds	Class	T2	T3	VIP	p_value	FDR	Fold_Change	Type
C3H7NO3	L-Serine	Amino acids and derivatives	8.39E+04	8.39E+04	1.55	0.02	0.39	2.89	up
C8H10O	1-Phenylethanol	Phenolic acids	1.16E+05	1.16E+05	1.59	0.05	0.40	4.29	up
C5H7NO3	1-Pyrroline-4-hydroxy-2-carboxylic acid	Organic acids	6.10E+04	6.10E+04	1.05	0.22	0.53	0.44	down
C6H14N2O	N-Acetylputrescine	Alkaloids	2.98E+05	2.98E+05	1.36	0.13	0.47	2.90	up
C5H8O4	Glutaric acid*	Organic acids	1.19E+06	1.19E+06	1.66	0.23	0.53	0.00	down
C6H12O3	2-Hydroxy-4-methylpentanoic acid	Organic acids	2.86E+06	2.86E+06	1.40	0.04	0.40	0.48	down
C8H8O2	4-Hydroxyacetophenone*	Phenolic acids	1.21E+05	1.21E+05	1.07	0.26	0.55	0.40	down
C8H8O2	Phenyl acetate*	Phenolic acids	1.13E+05	1.13E+05	1.23	0.22	0.53	0.38	down
C5H8O5	L-Citramalic acid	Organic acids	8.63E+05	8.63E+05	1.12	0.18	0.50	2.46	up
C10H12O2	Ethyl phenylacetate	Phenolic acids	1.47E+05	1.47E+05	1.59	0.06	0.40	5.65	up
C10H9NO2	1-Methoxyindole-3-carbaldehyde	Alkaloids	1.57E+04	1.57E+04	1.66	0.02	0.39	0.00	down

C10H8O3	7-Methoxycoumarin	Lignans and Coumarins	5.30E+05	5.30E+05	1.03	0.20	0.52	0.48	down
C11H12O2	Ethyl cinnamate	Phenolic acids	1.51E+04	1.51E+04	1.55	0.03	0.40	2.47	up
C9H8O4	Caffeic acid	Phenolic acids	3.85E+06	3.85E+06	1.07	0.24	0.54	0.35	down
C7H12N2O4	N-Acetyl-L-Glutamine	Amino acids and derivatives	9.00E+00	9.00E+00	1.66	0.02	0.39	9088.67	up
C10H9NO3	5-Hydroxyindole-3-acetic acid	Alkaloids	6.50E+04	6.50E+04	1.57	0.02	0.39	2.19	up
C12H24O3	12-Hydroxydodecanoic acid	Lipids	4.34E+04	4.34E+04	1.66	0.05	0.40	0.00	down
C12H22O4	Dodecanedioic aicd	Organic acids	4.89E+03	4.89E+03	1.52	0.01	0.39	0.32	down
C10H22N2O4	L-Lysine-Butanoic Acid	Amino acids and derivatives	9.00E+00	9.00E+00	1.66	0.04	0.40	6655.19	up
C9H13N3O5	Cytarabine	Nucleotides and derivatives	2.83E+06	2.83E+06	1.58	0.03	0.40	0.43	down
C9H12N2O6	Uridine	Nucleotides and derivatives	1.29E+06	1.29E+06	1.34	0.06	0.40	0.43	down
C10H13N5O3	2'-Deoxyadenosine	Nucleotides and derivatives	4.04E+05	4.04E+05	1.24	0.16	0.49	0.48	down
C9H17NO6S	S-Ribosyl-L-homocysteine	Amino acids and derivatives	1.45E+06	1.45E+06	1.58	0.04	0.40	0.36	down
C10H13N5O4	Vidarabine*	Others	2.78E+06	2.78E+06	1.64	0.02	0.39	0.34	down
C10H13N5O4	Adenosine*	Nucleotides and derivatives	2.78E+06	2.78E+06	1.57	0.05	0.40	0.39	down
C15H10O5	Apigenin; 4',5,7-Trihydroxyflavone	Flavonoids	5.47E+04	5.47E+04	1.51	0.07	0.41	3.22	up
C15H10O5	3,5,7-Trihydroxyflavone	Flavonoids	1.01E+05	1.01E+05	1.60	0.02	0.40	2.95	up
C15H14O5	Phloretin	Flavonoids	5.78E+05	5.78E+05	1.45	0.05	0.40	2.10	up
C14H17NO5	4-(Rhamnosyloxy)phenylacetonitrile	Alkaloids	3.40E+05	3.40E+05	1.58	0.02	0.39	0.36	down
C13H12O7	p-Coumaroylmalic acid	Phenolic acids	1.92E+05	1.92E+05	1.05	0.19	0.51	0.35	down
C10H13N5O5	Guanosine	Nucleotides and derivatives	7.61E+06	7.61E+06	1.50	0.01	0.39	0.37	down
C17H17NO3	p-Coumaroyltyramine	Alkaloids	9.00E+00	9.00E+00	1.66	0.01	0.39	30782.22	up
C17H16O4	Phenethyl caffeate	Phenolic acids	6.37E+04	6.37E+04	1.46	0.09	0.44	3.51	up
C15H10O6	Isoscutellarein*	Flavonoids	2.41E+04	2.41E+04	1.59	0.04	0.40	2.83	up
C15H10O6	2'-Hydroxygenistein*	Flavonoids	1.98E+04	1.98E+04	1.35	0.04	0.40	3.73	up
C15H10O6	Luteolin (5,7,3',4'-Tetrahydroxyflavone)*	Flavonoids	1.98E+04	1.98E+04	1.35	0.04	0.40	3.73	up
C18H30O3	17-Hydroxylinolenic acid	Lipids	1.59E+05	1.59E+05	1.02	0.30	0.57	2.38	up

C18H30O3	9-Oxo-10E,12Z-octadecadienoic acid	Lipids	8.22E+04	8.22E+04	1.32	0.22	0.53	0.41	down
C13H12O8	2-O-Caffeoylmalic acid	Phenolic acids	4.60E+05	4.60E+05	1.37	0.17	0.50	0.16	down
C18H34O3	Ricinoleic acid	Lipids	7.69E+04	7.69E+04	1.17	0.28	0.55	2.63	up
C20H42O	1-Eicosanol	Lipids	7.44E+04	7.44E+04	1.18	0.27	0.55	2.64	up
C17H34O4	1-Monomyristin	Lipids	2.68E+05	2.68E+05	1.49	0.01	0.39	2.73	up
C10H17N3O6S	Glutathione reduced form	Amino acids and derivatives	5.30E+05	5.30E+05	1.34	0.12	0.46	2.17	up
C18H32O4	13S-Hydroperoxy-9Z,11E-octadecadienoic acid	Lipids	6.09E+04	6.09E+04	1.37	0.14	0.47	2.58	up
C18H34O4	Hydroxy ricinoleic acid	Lipids	1.12E+05	1.12E+05	1.59	0.00	0.27	2.90	up
C16H12O7	Isorhamnetin	Flavonoids	4.32E+05	4.32E+05	1.56	0.03	0.40	3.51	up
C11H19N3O6S	S-(Methyl)glutathione	Amino acids and derivatives	1.02E+05	1.02E+05	1.21	0.06	0.40	0.49	down
C18H29NO4	(-)-Jasmonoyl-L-Isoleucine	Organic acids	7.80E+04	7.80E+04	1.13	0.14	0.47	0.38	down
C20H36O3	Ethyl 9-Hydroxy-10,12-octadecadienoic acid	Lipids	6.57E+04	6.57E+04	1.16	0.25	0.55	0.37	down
C20H39NO2	N-Oleoylethanolamine	Alkaloids	4.11E+05	4.11E+05	1.14	0.26	0.55	0.39	down
C15H18O8	(2E)-3-[4-(β-D-glucopyranoside)-phenylacrylic]-acid	Phenolic acids	5.72E+04	5.72E+04	1.15	0.13	0.47	2.29	up
C15H18O8	Phenylpropionic acid-O-β-D-glucopyranoside	Phenolic acids	5.72E+04	5.72E+04	1.15	0.13	0.47	2.29	up
C20H22O4	(+)-trans-1,2-dihydrodehydroguaiaretic acid	Lignans and Coumarins	3.76E+03	3.76E+03	1.44	0.08	0.41	4.71	up
C18H32O5	9,12,13-Trihydroxy-10,15-octadecadienoic acid	Lipids	1.57E+05	1.57E+05	1.59	0.03	0.40	0.21	down
C10H12N5O6P	Cyclic 3',5'-Adenylic acid	Nucleotides and derivatives	1.48E+05	1.48E+05	1.60	0.03	0.40	0.39	down
C14H18O9	5-Glucosyloxy-2-Hydroxybenzoic acid methyl ester*	Phenolic acids	1.71E+05	1.71E+05	1.47	0.02	0.39	0.47	down
C15H18O9	1-O-Caffeoyl-β-D-glucose*	Phenolic acids	3.52E+05	3.52E+05	1.14	0.35	0.59	0.42	down
C18H16O7	4',5-Dihydroxy-3',6,7-trimethoxyflavone	Flavonoids	1.04E+04	1.04E+04	1.42	0.10	0.46	0.04	down
C10H12N5O7P	Guanosine 3',5'-cyclic monophosphate	Nucleotides and derivatives	4.38E+05	4.38E+05	1.31	0.14	0.47	0.27	down
C21H38O4	1-Monolinolein	Lipids	1.99E+05	1.99E+05	1.66	0.11	0.46	0.00	down
C20H20O7	4',5,6,7,8-Pentamethoxyflavone	Flavonoids	5.67E+04	5.67E+04	1.53	0.11	0.46	0.18	down
C18H28O9	5'-Glucosyloxyjasmanic acid	Phenolic acids	4.80E+04	4.80E+04	1.12	0.12	0.46	2.28	up
C21H22O8	5,6,7,8,3',4'-Hexamethoxyflavone	Flavonoids	7.84E+04	7.84E+04	1.22	0.32	0.58	0.17	down

C12H23O14P	Trehalose 6-phosphate	Others	7.50E+04	7.50E+04	1.44	0.02	0.39	0.45	down
C19H40NO7P	LysoPE 14:0(2n isomer)	Lipids	1.71E+05	1.71E+05	1.45	0.10	0.46	0.33	down
C19H40NO7P	LysoPE 14:0	Lipids	1.45E+05	1.45E+05	1.40	0.10	0.45	0.42	down
C10H15N5O10P2	Adenosine 5'-diphosphate	Nucleotides and derivatives	3.24E+04	3.24E+04	1.18	0.26	0.55	3.51	up
C22H22O9	Formononetin-7-O-glycoside (Ononin)	Flavonoids	2.62E+04	2.62E+04	1.66	0.06	0.40	0.00	down
C20H40NO7P	LysoPE 15:1(2n isomer)	Lipids	4.06E+04	4.06E+04	1.40	0.04	0.40	0.42	down
C21H20O11	Kaempferol-4'-O-glucoside*	Flavonoids	9.33E+05	9.33E+05	1.62	0.03	0.40	3.32	up
C21H22O11	Dihydrokaempferol-7-O-glucoside*	Flavonoids	7.65E+05	7.65E+05	1.18	0.21	0.52	0.32	down
C21H22O11	Eriodictyol-3'-O-glucoside*	Flavonoids	7.53E+05	7.53E+05	1.26	0.18	0.50	0.32	down
C23H20O10	Cinnamoylferuloyltartaric acid	Phenolic acids	3.73E+04	3.73E+04	1.41	0.07	0.41	2.00	up
C30H48O3	(23S)-3β-hydroxydammara-21-oic acid 21,23-lactone	Terpenoids	6.24E+03	6.24E+03	1.63	0.26	0.55	0.00	down
C22H44NO7P	LysoPE 17:1(2n isomer)	Lipids	1.10E+05	1.10E+05	1.41	0.11	0.46	0.49	down
C22H46NO7P	LysoPC 14:0	Lipids	1.04E+06	1.04E+06	1.51	0.09	0.44	0.29	down
C29H41NO4	O-Acetyljervine	Alkaloids	1.05E+06	1.05E+06	1.47	0.12	0.46	0.32	down
C22H22O12	Isotamarixin	Flavonoids	2.75E+05	2.75E+05	1.31	0.19	0.51	2.40	up
C23H46NO7P	LysoPC 15:1	Lipids	2.71E+05	2.71E+05	1.61	0.04	0.40	0.41	down
C23H48NO7P	LysoPC 15:0(2n isomer)	Lipids	1.97E+05	1.97E+05	1.01	0.30	0.57	0.46	down
C23H48NO7P	LysoPC 15:0	Lipids	1.41E+06	1.41E+06	1.37	0.11	0.46	0.49	down
C30H46O5	2α,19α-Dihydroxy-3-oxours-12-en-28-oic acid	Terpenoids	8.27E+05	8.27E+05	1.16	0.23	0.53	2.29	up
C24H48NO7P	LysoPC 16:1(2n isomer)	Lipids	1.58E+06	1.58E+06	1.38	0.13	0.47	0.28	down
C22H22O13	Mearnsetin-3-O-glucoside	Flavonoids	1.24E+06	1.24E+06	1.12	0.32	0.58	0.34	down
C18H32O16	D-Panose	Others	3.05E+05	3.05E+05	1.45	0.02	0.39	0.19	down
C18H32O16	Maltotriose	Others	1.24E+06	1.24E+06	1.47	0.06	0.40	0.22	down
C25H48NO7P	LysoPC 17:2	Lipids	2.72E+05	2.72E+05	1.15	0.25	0.55	0.42	down
C25H52NO7P	LysoPC 17:0	Lipids	2.63E+05	2.63E+05	1.48	0.14	0.47	0.33	down
C27H46O9	1-α-Linolenoyl-glycerol-3-O-glucoside	Lipids	4.82E+05	4.82E+05	1.57	0.02	0.39	0.21	down

C27H48O9	1-Linoleoylglycerol-3-O-glucoside	Lipids	1.08E+05	1.08E+05	1.27	0.16	0.49	0.33	down
C26H50NO7P	LysoPC 18:2(2n isomer)	Lipids	7.92E+06	7.92E+06	1.49	0.05	0.40	0.45	down
C26H32O11	Matairesinol-4'-O-glucoside	Lignans and Coumarins	4.94E+05	4.94E+05	1.29	0.14	0.47	2.30	up
C14H22N2O16P2	Uridine-5'-Diphosphate-D-Xylose	Nucleotides and derivatives	6.12E+04	6.12E+04	1.41	0.11	0.46	2.48	up
C28H50NO7P	LysoPC 20:4	Lipids	5.99E+04	5.99E+04	1.61	0.36	0.59	0.00	down
C29H39N3O8	N1,N8-Bis(sinapoyl)spermidine	Alkaloids	4.00E+05	4.00E+05	1.26	0.05	0.40	0.48	down
C26H26O14	Sinapoylsinapoyltartaric acid	Phenolic acids	1.61E+04	1.61E+04	1.67	0.00	0.25	0.00	down
C29H36O13	Medioresinol-4'-O-(6'''-acetyl)glucoside	Lignans and Coumarins	4.30E+04	4.30E+04	1.43	0.11	0.46	0.37	down
C30H38O14	Syringaresinol-4'-O-(6''-acetyl)glucoside	Lignans and Coumarins	1.07E+06	1.07E+06	1.22	0.07	0.41	0.43	down
C24H42O21	Nystose	Others	6.38E+04	6.38E+04	1.04	0.00	0.37	0.14	down
C24H42O21	D-Maltotetraose	Others	9.17E+04	9.17E+04	1.31	0.10	0.46	0.18	down
C24H42O21	Stachyose	Others	3.31E+05	3.31E+05	1.50	0.03	0.40	0.22	down
C33H56O14	Gingerglycolipid A	Lipids	3.05E+06	3.05E+06	1.50	0.08	0.41	0.37	down
C33H56O14	1-α-Linolenoyl-glycerol-2,3-di-O-glucoside	Lipids	6.52E+05	6.52E+05	1.49	0.07	0.41	0.42	down
C33H56O14	1-Linolenoyl-rac-glycerol-diglucoside	Lipids	2.21E+06	2.21E+06	1.63	0.01	0.39	0.42	down
C33H56O14	2-α-Linolenoyl-glycerol-1,3-di-O-glucoside	Lipids	3.95E+05	3.95E+05	1.39	0.13	0.47	0.38	down
C34H30O15	3,4,5-Tricaffeoylquinic acid	Phenolic acids	4.03E+06	4.03E+06	1.44	0.01	0.39	0.30	down
C31H34O17	1,5-O-dicaffeoyl-3-O-glucoside-quinic acid	Phenolic acids	3.97E+06	3.97E+06	1.35	0.03	0.40	0.36	down
C33H58O14	Gingerglycolipid B	Lipids	3.12E+05	3.12E+05	1.38	0.12	0.46	0.36	down
C38H60O10	3-O-(2-O-Acetyl-glucosyl)oleanolic acid	Terpenoids	2.33E+06	2.33E+06	1.20	0.17	0.50	0.43	down
C33H60O14	Gingerglycolipid C	Lipids	2.83E+04	2.83E+04	1.23	0.25	0.55	0.38	down
C30H32O20	Quercetin-7-O-(6''-malonyl)glucosyl-5-O-glucoside	Flavonoids	2.95E+04	2.95E+04	1.27	0.02	0.39	0.14	down
C38H34O19	3,5-Di-O-caffeoyl-1-O-(2-O-caffeoylmaloyl)-quinic acid	Phenolic acids	7.55E+07	7.55E+07	1.13	0.08	0.41	0.49	down
C35H38O21	3,5-Di-O-caffeoyl-1-O-(2-O-glucosylmaloyl)-quinic acid*	Phenolic acids	1.32E+06	1.32E+06	1.20	0.05	0.40	0.38	down
C35H38O21	3,5-Di-O-caffeoyl-1-O-(4-O-glucosylmaloyl)-quinic acid*	Phenolic acids	1.32E+06	1.32E+06	1.20	0.05	0.40	0.38	down
C30H52O26	Verbascose	Others	6.97E+04	6.97E+04	1.33	0.18	0.50	0.12	down

Table S5. Variation of differential metabolite expression content in group T2 vs T4.

Formula	Compounds	Class	T2	T4	VIP	p_value	FDR	Fold_Change	Type
C3H7NO2	N-Methylglycine	Amino acids and derivatives	4.67E+03	4.67E+03	1.25	0.10	0.28	3.60	up
C3H7NO3	L-Serine	Amino acids and derivatives	8.39E+04	8.39E+04	1.40	0.00	0.15	3.04	up
C5H10O3	β-Hydroxyisovaleric acid	Organic acids	1.78E+06	1.78E+06	1.08	0.11	0.29	0.49	down
C8H9N	N-Benzylmethylene isomethylamine	Alkaloids	1.05E+07	1.05E+07	1.38	0.07	0.25	2.13	up
C8H8O	(S)-2-Phenyloxirane	Others	1.37E+07	1.37E+07	1.01	0.18	0.36	0.32	down
C6H6N2O	Nicotinamide	Others	1.09E+06	1.09E+06	1.36	0.05	0.24	0.41	down
C6H11NO2	L-Pipecolic Acid	Organic acids	2.38E+07	2.38E+07	1.46	0.01	0.15	2.05	up
C6H14N2O	N-Acetylputrescine	Alkaloids	2.98E+05	2.98E+05	1.29	0.01	0.15	2.25	up
C4H9N3O2	3-Guanidinopropionic acid	Organic acids	3.14E+04	3.14E+04	1.33	0.08	0.26	3.26	up
C8H8O2	4-Hydroxyacetophenone*	Phenolic acids	1.21E+05	1.21E+05	1.10	0.22	0.39	0.33	down
C8H8O2	Benzoic acid methyl ester	Phenolic acids	9.00E+00	9.00E+00	1.04	0.18	0.36	2773.30	up
C8H8O2	Phenyl acetate*	Phenolic acids	1.13E+05	1.13E+05	1.21	0.20	0.37	0.33	down
C9H12O	(Ethoxymethyl)benzene	Others	8.83E+04	8.83E+04	1.47	0.03	0.22	0.00	down
C8H11NO	L-Tyramine	Alkaloids	1.48E+07	1.48E+07	1.30	0.13	0.31	0.07	down
C5H8O5	2-Hydroxyglutaric Acid*	Organic acids	4.87E+05	4.87E+05	1.35	0.03	0.21	2.30	up
C5H8O5	2-Dehydro-3-deoxy-L-arabinonate*	Others	4.79E+05	4.79E+05	1.43	0.01	0.15	2.35	up
C5H8O5	L-Citramalic acid	Organic acids	8.63E+05	8.63E+05	1.20	0.05	0.25	2.48	up
C10H12O	2',4'-Dimethylacetophenone	Others	1.28E+05	1.28E+05	1.47	0.01	0.15	0.00	down
C10H12O	Mesitaldehyde	Others	1.28E+05	1.28E+05	1.47	0.01	0.15	0.00	down
C8H7NO2	4-Hydroxymandelonitrile	Alkaloids	2.42E+06	2.42E+06	1.29	0.13	0.31	2.77	up
C5H11NO2S	L-Methionine	Amino acids and derivatives	2.29E+06	2.29E+06	1.25	0.13	0.32	2.57	up
C10H12N2	Tryptamine	Alkaloids	7.44E+05	7.44E+05	1.41	0.06	0.25	0.25	down
C6H11NO4	N-Acetyl-L-threonine	Amino acids and derivatives	1.04E+05	1.04E+05	1.27	0.10	0.28	2.67	up

C6H10O5	3-Hydroxy-3-methylpentane-1,5-dioic acid	Amino acids and derivatives	1.72E+06	1.72E+06	1.36	0.02	0.20	2.84	up
C9H11NO2	L-Phenylalanine	Amino acids and derivatives	1.74E+07	1.74E+07	1.40	0.05	0.25	2.12	up
C8H15NO3	N-Acetyl-L-leucine	Amino acids and derivatives	1.50E+05	1.50E+05	1.39	0.07	0.25	2.90	up
C6H9NO5	N-Acetyl-L-Aspartic Acid	Amino acids and derivatives	5.18E+04	5.18E+04	1.35	0.04	0.23	3.80	up
C6H8O6	D-Glucurono-6,3-lactone	Others	3.09E+04	3.09E+04	1.47	0.02	0.21	0.00	down
C10H8O3	7-Methoxycoumarin	Lignans and Coumarins	5.30E+05	5.30E+05	1.13	0.14	0.32	0.35	down
C6H5N5O2	Isoxanthopterin	Nucleotides and derivatives	2.21E+05	2.21E+05	1.41	0.01	0.17	0.49	down
C7H12N2O4	N-Acetyl-L-Glutamine	Amino acids and derivatives	9.00E+00	9.00E+00	1.47	0.07	0.26	376840.74	up
C7H11NO5	N-Acetyl-L-glutamic acid	Amino acids and derivatives	3.62E+05	3.62E+05	1.27	0.09	0.28	3.00	up
C6H10O7	D-Glucoronic acid	Others	1.43E+05	1.43E+05	1.46	0.00	0.11	2.46	up
C4H9O7P	D-Erythrose-4-phosphate	Others	4.45E+04	4.45E+04	1.24	0.09	0.28	2.44	up
C11H12O4	Sinapinaldehyde	Phenolic acids	3.01E+05	3.01E+05	1.44	0.01	0.15	2.53	up
C12H24O3	12-Hydroxydodecanoic acid	Lipids	4.34E+04	4.34E+04	1.47	0.05	0.24	0.00	down
C15H24O	Santalol	Others	8.26E+05	8.26E+05	1.47	0.03	0.21	0.00	down
C11H13NO4	N-Acetyl-L-tyrosine	Amino acids and derivatives	5.67E+04	5.67E+04	1.46	0.01	0.15	9.88	up
C12H22O4	Dodecanedioic aicd	Organic acids	4.89E+03	4.89E+03	1.40	0.00	0.15	0.37	down
C15H24O2	Baimuxinal	Terpenoids	5.69E+04	5.69E+04	1.09	0.12	0.31	0.15	down
C10H14N2O5	Thymidine	Nucleotides and derivatives	5.85E+04	5.85E+04	1.17	0.18	0.36	0.46	down
C9H13N3O5	Cytidine	Nucleotides and derivatives	1.66E+05	1.66E+05	1.45	0.01	0.15	0.34	down
C9H13N3O5	Cytarabine	Nucleotides and derivatives	2.83E+06	2.83E+06	1.39	0.02	0.20	0.34	down
C9H12N2O6	Uridine	Nucleotides and derivatives	1.29E+06	1.29E+06	1.38	0.05	0.25	0.32	down
C10H13N5O3	2'-Deoxyadenosine	Nucleotides and derivatives	4.04E+05	4.04E+05	1.20	0.13	0.31	0.40	down
C15H12O4	Pinocembrin (Dihydrochrysin)	Flavonoids	3.12E+04	3.12E+04	1.39	0.01	0.16	0.42	down
C8H20NO6P	Choline Alfoscerate	Lipids	7.97E+05	7.97E+05	1.29	0.01	0.17	3.85	up
C14H18N2O3	L-Prolyl-L-Phenylalanine	Amino acids and derivatives	3.92E+05	3.92E+05	1.34	0.03	0.22	2.11	up
C16H12O4	Formononetin (7-Hydroxy-4'-methoxyisoflavone)	Flavonoids	1.52E+04	1.52E+04	1.33	0.05	0.24	0.38	down

C10H12N4O5	9-(Arabinosyl)hypoxanthine	Nucleotides and derivatives	3.12E+05	3.12E+05	1.42	0.00	0.15	0.31	down
C18H30O2	α-Linolenic Acid	Lipids	2.58E+07	2.58E+07	1.35	0.08	0.27	0.48	down
C18H30O2	γ-Linolenic Acid	Lipids	4.90E+05	4.90E+05	1.41	0.05	0.24	0.46	down
C18H30O2	Crepenynic acid	Lipids	5.02E+05	5.02E+05	1.39	0.06	0.25	0.46	down
C10H13N5O5	Guanosine	Nucleotides and derivatives	7.61E+06	7.61E+06	1.43	0.02	0.20	0.41	down
C16H12O5	Apigenin 7-methyl ether*	Flavonoids	4.39E+05	4.39E+05	1.45	0.01	0.16	0.43	down
C16H12O5	Acacetin*	Flavonoids	4.27E+05	4.27E+05	1.41	0.01	0.15	0.41	down
C16H12O5	Prunetin (5,4'-Dihydroxy-7-methoxyisoflavone)	Flavonoids	4.49E+05	4.49E+05	1.44	0.01	0.15	0.40	down
C16H12O5	5,7-Dihydroxy-8-Methoxyflavone*	Flavonoids	5.61E+05	5.61E+05	1.45	0.00	0.15	0.42	down
C15H17N3O3	Feruloylhistamine	Alkaloids	1.17E+04	1.17E+04	1.47	0.04	0.23	0.00	down
C16H32O4	10,16-Dihydroxypalmitic acid	Lipids	3.55E+04	3.55E+04	1.22	0.15	0.33	0.46	down
C17H23NO3	L-Hyoscyamine	Alkaloids	3.11E+04	3.11E+04	1.08	0.04	0.23	0.37	down
C18H30O3	17-Hydroxylinolenic acid	Lipids	1.59E+05	1.59E+05	1.05	0.21	0.38	2.75	up
C18H30O3	2R-hydroxy-9Z,12Z,15Z-octadecatrienoic acid	Lipids	1.03E+05	1.03E+05	1.11	0.26	0.43	3.57	up
C18H30O3	9-Oxo-10E,12Z-octadecadienoic acid	Lipids	8.22E+04	8.22E+04	1.20	0.18	0.36	0.32	down
C8H14N3O7P	5-Aminoimidazole ribonucleotide	Nucleotides and derivatives	1.33E+06	1.33E+06	1.33	0.01	0.15	4.08	up
C18H34O3	Ricinoleic acid	Lipids	7.69E+04	7.69E+04	1.22	0.20	0.37	3.25	up
C20H42O	1-Eicosanol	Lipids	7.44E+04	7.44E+04	1.25	0.16	0.34	3.09	up
C18H36O3	12-Hydroxyoctadecanoic acid	Lipids	2.54E+04	2.54E+04	1.47	0.02	0.21	0.00	down
C18H36O3	2R-Hydroxyoctadecanoic Acid	Lipids	1.34E+04	1.34E+04	1.33	0.05	0.25	2.71	up
C15H14O7	Epigallocatechin	Flavonoids	9.00E+00	9.00E+00	1.47	0.10	0.29	2647.89	up
C10H17N3O6S	Glutathione reduced form	Amino acids and derivatives	5.30E+05	5.30E+05	1.38	0.01	0.18	2.05	up
C18H32O4	13S-Hydroperoxy-9Z,11E-octadecadienoic acid	Lipids	6.09E+04	6.09E+04	1.22	0.20	0.37	3.72	up
C18H34O4	12,13-DHOME; (9Z)-12,13-Dihydroxyoctadec-9-enoic acid	Lipids	2.63E+04	2.63E+04	1.39	0.06	0.25	2.21	up
C13H16O9	Protocatechuic acid-4-O-glucoside*	Phenolic acids	6.38E+06	6.38E+06	1.26	0.05	0.24	0.48	down
C9H13N2O9P	Uridine 5'-monophosphate	Nucleotides and derivatives	1.31E+05	1.31E+05	1.41	0.05	0.25	0.27	down

C18H32O5	9,12,13-Trihydroxy-10,15-octadecadienoic acid	Lipids	1.57E+05	1.57E+05	1.39	0.02	0.21	0.17	down
C18H34O5	9,12,13-TriHOME; 9(S),12(S),13(S)-Trihydroxy-10(E)-octadecenoic acid	Lipids	2.75E+04	2.75E+04	1.29	0.17	0.35	5.44	up
C6H14O12P2	D-Glucose 1,6-bisphosphate	Others	6.49E+03	6.49E+03	1.21	0.10	0.29	3.06	up
C15H18O9	1-O-Caffeoyl-β-D-glucose*	Phenolic acids	3.52E+05	3.52E+05	1.24	0.02	0.19	2.95	up
C18H16O7	4',5-Dihydroxy-3',6,7-trimethoxyflavone	Flavonoids	1.04E+04	1.04E+04	1.33	0.14	0.32	0.20	down
C10H12N5O7P	Guanosine 3',5'-cyclic monophosphate	Nucleotides and derivatives	4.38E+05	4.38E+05	1.39	0.10	0.29	0.09	down
C14H18O10	1-O-(3,4-Dihydroxy-5-methoxy-benzoyl)-glucoside	Phenolic acids	2.56E+04	2.56E+04	1.46	0.02	0.20	5.73	up
C21H38O4	1-Monolinolein	Lipids	1.99E+05	1.99E+05	1.39	0.13	0.31	0.10	down
C16H16O10	Scopoletin-7-O-glucuronide	Lignans and Coumarins	2.75E+06	2.75E+06	1.28	0.04	0.23	2.30	up
C17H20O9	Chlorogenic acid methyl ester	Phenolic acids	4.64E+06	4.64E+06	1.25	0.03	0.21	2.02	up
C20H20O7	4',5,6,7,8-Pentamethoxyflavone	Flavonoids	5.67E+04	5.67E+04	1.21	0.17	0.35	0.36	down
C19H40NO7P	LysoPE 14:0(2n isomer)	Lipids	1.71E+05	1.71E+05	1.38	0.08	0.27	0.22	down
C19H40NO7P	LysoPE 14:0	Lipids	1.45E+05	1.45E+05	1.38	0.06	0.25	0.25	down
C20H30NO9+	Caffeoylcholine-4-O-glucoside	Alkaloids	1.40E+05	1.40E+05	1.02	0.15	0.33	0.44	down
C22H22O9	1-O-Feruloyl-3-O-caffeoylglycerol	Lipids	2.45E+06	2.45E+06	1.24	0.01	0.18	0.35	down
C22H22O9	Formononetin-7-O-glycoside (Ononin)	Flavonoids	2.62E+04	2.62E+04	1.47	0.06	0.25	0.00	down
C20H18O11	Quercetin-3-O-xyloside*	Flavonoids	8.64E+05	8.64E+05	1.44	0.00	0.15	2.13	up
C20H18O11	Quercetin-3-O-α-L-arabinofuranoside*	Flavonoids	6.22E+05	6.22E+05	1.44	0.01	0.17	2.73	up
C20H40NO7P	LysoPE 15:1(2n isomer)	Lipids	4.06E+04	4.06E+04	1.41	0.03	0.21	0.21	down
C20H40NO7P	LysoPE 15:1	Lipids	3.12E+04	3.12E+04	1.22	0.03	0.21	0.46	down
C20H42NO7P	LysoPE 15:0	Lipids	3.34E+05	3.34E+05	1.30	0.10	0.29	0.29	down
C20H42NO7P	LysoPE 15:0(2n isomer)	Lipids	3.17E+05	3.17E+05	1.34	0.08	0.27	0.28	down
C21H22O11	6-C-Glucosyl-2-Hydroxynaringenin	Flavonoids	2.19E+07	2.19E+07	1.42	0.01	0.15	0.44	down
C21H42NO7P	LysoPE 16:1	Lipids	3.21E+05	3.21E+05	1.25	0.08	0.27	0.30	down
C21H42NO7P	LysoPE 16:1(2n isomer)	Lipids	2.82E+05	2.82E+05	1.15	0.14	0.32	0.31	down
C21H44NO7P	LysoPE 16:0(2n isomer)	Lipids	2.77E+07	2.77E+07	1.35	0.04	0.24	0.43	down

C21H44NO7P	LysoPE 16:0	Lipids	3.02E+07	3.02E+07	1.36	0.05	0.25	0.40	down
C23H20O10	Cinnamoylferuloyltartaric acid	Phenolic acids	3.73E+04	3.73E+04	1.47	0.02	0.20	0.00	down
C19H27NO12	Anthranilate-1-O-Sophoroside	Phenolic acids	4.52E+05	4.52E+05	1.14	0.30	0.47	5.49	up
C21H18O12	Vnilloylcaffeoyltartaric acid	Phenolic acids	3.27E+04	3.27E+04	1.45	0.00	0.08	2.82	up
C22H44NO7P	LysoPE 17:1(2n isomer)	Lipids	1.10E+05	1.10E+05	1.26	0.06	0.25	0.39	down
C22H44NO7P	LysoPE 17:1	Lipids	1.21E+05	1.21E+05	1.37	0.05	0.25	0.37	down
C21H22O12	Taxifolin-3'-O-glucoside	Flavonoids	1.54E+05	1.54E+05	1.15	0.06	0.25	2.48	up
C22H46NO7P	LysoPC 14:0	Lipids	1.04E+06	1.04E+06	1.40	0.08	0.26	0.20	down
C29H41NO4	O-Acetyljervine	Alkaloids	1.05E+06	1.05E+06	1.38	0.09	0.28	0.21	down
C23H42NO7P	LysoPE 18:3	Lipids	1.49E+07	1.49E+07	1.34	0.06	0.25	0.49	down
C23H44NO7P	LysoPE 18:2	Lipids	1.99E+07	1.99E+07	1.24	0.08	0.27	0.47	down
C23H44NO7P	LysoPE 18:2(2n isomer)	Lipids	1.56E+07	1.56E+07	1.22	0.09	0.28	0.49	down
C23H46NO7P	LysoPE 18:1	Lipids	2.17E+06	2.17E+06	1.34	0.07	0.25	0.40	down
C23H46NO7P	LysoPC 15:1	Lipids	2.71E+05	2.71E+05	1.41	0.01	0.16	0.23	down
C23H46NO7P	LysoPE 18:1(2n isomer)	Lipids	1.32E+06	1.32E+06	1.36	0.07	0.25	0.35	down
C23H48NO7P	LysoPE 18:0(2n isomer)	Lipids	2.64E+04	2.64E+04	1.26	0.07	0.26	0.37	down
C23H48NO7P	LysoPC 15:0(2n isomer)	Lipids	1.97E+05	1.97E+05	1.14	0.22	0.38	0.31	down
C23H48NO7P	LysoPE 18:0	Lipids	1.40E+05	1.40E+05	1.32	0.09	0.28	0.42	down
C23H48NO7P	LysoPC 15:0	Lipids	1.41E+06	1.41E+06	1.36	0.06	0.25	0.29	down
C24H46NO7P	LysoPC 16:2(2n isomer)	Lipids	8.03E+04	8.03E+04	1.21	0.15	0.33	0.33	down
C24H48NO7P	LysoPC 16:1	Lipids	1.06E+06	1.06E+06	1.20	0.10	0.28	0.32	down
C24H48NO7P	LysoPC 16:1(2n isomer)	Lipids	1.58E+06	1.58E+06	1.31	0.12	0.30	0.22	down
C24H50NO7P	LysoPC 16:0(2n isomer)	Lipids	8.75E+07	8.75E+07	1.41	0.02	0.18	0.45	down
C24H50NO7P	LysoPC 16:0	Lipids	8.75E+07	8.75E+07	1.40	0.02	0.20	0.45	down
C25H46NO7P	LysoPE 20:3(2n isomer)	Lipids	4.44E+04	4.44E+04	1.37	0.06	0.25	0.29	down
C25H46NO7P	LysoPE 20:3	Lipids	4.56E+04	4.56E+04	1.30	0.08	0.27	0.33	down

C25H48NO7P	LysoPE 20:2(2n isomer)	Lipids	6.38E+04	6.38E+04	1.27	0.12	0.31	0.34	down
C25H48NO7P	LysoPC 17:2	Lipids	2.72E+05	2.72E+05	1.10	0.21	0.38	0.35	down
C25H48NO7P	LysoPE 20:2	Lipids	7.39E+04	7.39E+04	1.27	0.14	0.32	0.31	down
C23H22O13	Quercetin-3-O-(6''-O-acetyl)galactoside	Flavonoids	1.17E+05	1.17E+05	1.32	0.00	0.15	4.10	up
C25H50NO7P	LysoPC 17:1	Lipids	4.27E+05	4.27E+05	1.32	0.06	0.25	0.34	down
C25H52NO7P	LysoPC 17:0	Lipids	2.63E+05	2.63E+05	1.34	0.12	0.31	0.28	down
C25H52NO7P	LysoPC 17:0(2n isomer)	Lipids	1.50E+05	1.50E+05	1.38	0.06	0.25	0.27	down
C27H46O9	1-α-Linolenoyl-glycerol-3-O-glucoside	Lipids	4.82E+05	4.82E+05	1.45	0.02	0.20	0.17	down
C26H46NO7P	LysoPC 18:4	Lipids	3.03E+05	3.03E+05	1.22	0.16	0.34	0.32	down
C25H24O12	3,5-Dicaffeoylquinic acid	Phenolic acids	9.47E+06	9.47E+06	1.29	0.02	0.18	2.54	up
C25H24O12	1,3-O-Dicaffeoylquinic Acid	Phenolic acids	7.82E+06	7.82E+06	1.14	0.07	0.25	2.21	up
C27H48O9	1-Linoleoylglycerol-3-O-glucoside	Lipids	1.08E+05	1.08E+05	1.39	0.11	0.29	0.12	down
C26H48NO7P	LysoPC 18:3	Lipids	9.81E+07	9.81E+07	1.34	0.01	0.15	0.50	down
C26H48NO7P	LysoPC 18:3(2n isomer)	Lipids	4.35E+07	4.35E+07	1.38	0.00	0.15	0.42	down
C26H50NO7P	LysoPC 18:2(2n isomer)	Lipids	7.92E+06	7.92E+06	1.40	0.05	0.24	0.37	down
C26H32O11	Matairesinol-4'-O-glucoside	Lignans and Coumarins	4.94E+05	4.94E+05	1.41	0.06	0.25	0.21	down
C26H52NO7P	LysoPC 18:1	Lipids	7.77E+06	7.77E+06	1.36	0.07	0.25	0.41	down
C26H52NO7P	LysoPC 18:1(2n isomer)	Lipids	7.14E+06	7.14E+06	1.38	0.05	0.24	0.42	down
C24H26O13	Rosmarinic acid-3'-O-glucoside	Phenolic acids	1.69E+05	1.69E+05	1.41	0.02	0.20	0.21	down
C24H26O13	Centaurein	Flavonoids	8.19E+04	8.19E+04	1.19	0.06	0.25	2.03	up
C28H50NO7P	LysoPC 20:4	Lipids	5.99E+04	5.99E+04	1.43	0.36	0.52	0.00	down
C28H52NO7P	LysoPC 20:3	Lipids	1.12E+05	1.12E+05	1.33	0.08	0.27	0.46	down
C28H54NO7P	LysoPC 20:2(2n isomer)	Lipids	3.11E+05	3.11E+05	1.22	0.14	0.32	0.36	down
C28H54NO7P	LysoPC 20:2	Lipids	3.13E+05	3.13E+05	1.35	0.10	0.29	0.30	down
C26H26O14	Sinapoylsinapoyltartaric acid	Phenolic acids	1.61E+04	1.61E+04	1.32	0.02	0.20	0.50	down
C25H24O15	Tamarixetin-3-O-(6''-malonyl)glucoside*	Flavonoids	1.85E+06	1.85E+06	1.10	0.11	0.30	2.10	up

C26H28O16	Quercetin-3-O-sambubioside	Flavonoids	5.79E+05	5.79E+05	1.39	0.03	0.21	2.73	up
C27H32O16	Okanin-4'-O-gentiobioside	Flavonoids	8.02E+05	8.02E+05	1.34	0.01	0.17	0.44	down
C27H30O17	Myricetin-3-O-galactoside-3'-O-rhamnoside*	Flavonoids	5.12E+05	5.12E+05	1.42	0.02	0.20	2.34	up
C27H30O17	Myricetin-3-O-rutinoside*	Flavonoids	4.71E+05	4.71E+05	1.43	0.00	0.15	2.51	up
C33H56O14	Gingerglycolipid A	Lipids	3.05E+06	3.05E+06	1.15	0.10	0.29	0.49	down
C33H56O14	1-α-Linolenoyl-glycerol-2,3-di-O-glucoside	Lipids	6.52E+05	6.52E+05	1.26	0.07	0.26	0.44	down
C33H56O14	1-Linolenoyl-rac-glycerol-diglucoside	Lipids	2.21E+06	2.21E+06	1.43	0.01	0.15	0.37	down
C33H58O14	Gingerglycolipid B	Lipids	3.12E+05	3.12E+05	1.33	0.12	0.31	0.33	down
C33H60O14	Gingerglycolipid C	Lipids	2.83E+04	2.83E+04	1.30	0.18	0.36	0.21	down
C30H32O20	Quercetin-3-O-(6''-O-malonyl)glucosyl-5-O-glucoside	Flavonoids	3.22E+04	3.22E+04	1.40	0.07	0.25	6.69	up
C31H34O20	Isorhamnetin-3-O-(6''-malonyl)glucoside-7-O-glucoside	Flavonoids	5.76E+04	5.76E+04	1.38	0.06	0.25	4.59	up
C34H42O21	Isorhamnetin-3-O-sophoroside-7-O-rhamnoside	Flavonoids	2.31E+05	2.31E+05	1.10	0.12	0.30	2.30	up
